# Synthesis, In Vivo Anticonvulsant Activity Evaluation and In Silico Studies of Some Quinazolin-4(3H)-One Derivatives

**DOI:** 10.3390/molecules29091951

**Published:** 2024-04-24

**Authors:** Raluca Pele, Gabriel Marc, Cristina Mogoșan, Anamaria Apan, Ioana Ionuț, Brîndușa Tiperciuc, Cristina Moldovan, Cătălin Araniciu, Ilioara Oniga, Adrian Pîrnău, Laurian Vlase, Ovidiu Oniga

**Affiliations:** 1 Department of Pharmaceutical Chemistry, Faculty of Pharmacy, “Iuliu Hațieganu” University of Medicine and Pharmacy, 41 Victor Babeș Street, 400012 Cluj-Napoca, Romania; raluca.pele@umfcluj.ro (R.P.); marc.gabriel@umfcluj.ro (G.M.); ionut.ioana@umfcluj.ro (I.I.); btiperciuc@umfcluj.ro (B.T.); cmoldovan@umfcluj.ro (C.M.); ooniga@umfcluj.ro (O.O.); 2 Department of Pharmacology, Physiology and Pathophysiology, Faculty of Pharmacy, “Iuliu Hațieganu” University of Medicine and Pharmacy, 6A Louis Pasteur Street, 400349 Cluj-Napoca, Romania; anamaria.cristina@umfcluj.ro; 3 Department of Therapeutical Chemistry, Faculty of Pharmacy, “Iuliu Hațieganu” University of Medicine and Pharmacy, 12 Ion Creangă, 400010 Cluj-Napoca, Romania; araniciu.catalin@umfcluj.ro; 4 Department of Pharmacognosy, “Iuliu Hațieganu” University of Medicine and Pharmacy, 12 Ion Creangă, 400010 Cluj-Napoca, Romania; ioniga@umfcluj.ro; 5 National Institute for Research and Development of Isotopic and Molecular Technologies, 67-103 Donat Street, 400293 Cluj-Napoca, Romania; adrian.pirnau@itim-cj.ro; 6 Department of Pharmaceutical Technology and Biopharmaceutics, Faculty of Pharmacy, “Iuliu Hațieganu” University of Medicine and Pharmacy, 41 Victor Babeș Street, 400012 Cluj-Napoca, Romania; laurian.vlase@umfcluj.ro

**Keywords:** quinazolin-4(3H)-one, anticonvulsant activity, molecular docking, molecular dynamics

## Abstract

Two series, “**a**” and “**b**”, each consisting of nine chemical compounds, with 2,3-disubstituted quinazolin-4(3H)-one scaffold, were synthesized and evaluated for their anticonvulsant activity. They were investigated as dual potential positive allosteric modulators of the GABA_A_ receptor at the benzodiazepine binding site and inhibitors of carbonic anhydrase II. Quinazolin-4(3H)-one derivatives were evaluated in vivo (D_1–3_ = 50, 100, 150 mg/kg, administered intraperitoneally) using the pentylenetetrazole (PTZ)-induced seizure model in mice, with phenobarbital and diazepam, as reference anticonvulsant agents. The in silico studies suggested the compounds act as anticonvulsants by binding on the allosteric site of GABA_A_ receptor and not by inhibiting the carbonic anhydrase II, because the ligands-carbonic anhydrase II predicted complexes were unstable in the molecular dynamics simulations. The mechanism targeting GABA_A_ receptor was confirmed through the in vivo flumazenil antagonism assay. The pentylenetetrazole experimental anticonvulsant model indicated that the tested compounds, **1a**–**9a** and **1b**–**9b**, present a potential anticonvulsant activity. The evaluation, considering the percentage of protection against PTZ, latency until the onset of the first seizure, and reduction in the number of seizures, revealed more favorable results for the “**b**” series, particularly for compound **8b**.

## 1. Introduction

Epilepsy is a chronic neurological disorder, affecting 0.5–1% of the population and is characterized by unpredictable and unprovoked depolarization of neurons, manifested by seizures [1,2]. According to The International League Against Epilepsy (ILAE 2017), there are four types of epilepsy: focal, generalized, generalized and focal combined, and unknown. Seizures are also classified by their onset as: focal, generalized, and unknown [3,4,5]. This disorder has severe neurobiological, cognitive, and psychosocial consequences [1,2].

Antiepileptic drugs (AED) delay the onset of seizures, their frequency and duration, through three general mechanisms: (1) direct modulation of ion channels that are involved in neuronal membrane hyperpolarization (e.g., benzodiazepines, barbiturates), or depolarization (e.g., phenytoin, carbamazepine); (2) inhibition of excitatory neuronal transmission (e.g., lamotrigine); (3) enhancement of inhibitory neuronal transmission (e.g., tiagabine), or through multiple mechanisms of action (e.g., valproic acid) [1,2,6]. The AEDs currently available can only treat some subtypes of epilepsy and are often accompanied by numerous side effects, which reduce the patients’ adherence to therapy. In addition, there are patients with resistant forms of epilepsy who do not respond to existing anticonvulsant medication. For this reason, there is an urgent need to expand the therapeutic options to treat and to defeat this pathology.

γ-Aminobutyric acid (GABA) is one of the main inhibitory neurotransmitters in the cerebral cortex. It is released into the synapse, where it acts at one of two types of receptors: GABA_A_ and GABA_B_.

GABA_A_ receptors are ionotropic receptors, and this family of ligand-gated ion channels contains more heteropentameric complexes assembled from 19 subunits (*α*_1–6_, *β*_1–3_, *γ*_1–3_, *δ*, *ε*, *θ*, *π*, and *ρ*_1–3_). Typically, the pentameric GABA_A_ complex is composed of two *α* subunits, two *β* subunits, and a *γ* or *δ* subunit [7]. The GABA binding site is located between α- and β-subunits. The active site contains a central pore that allows chloride’s conductance through it. Once bound to GABA, the GABA_A_ receptors are activated. Upon opening, the GABA_A_ receptor is selectively permeable to chloride (Cl^−^) and bicarbonate (HCO_3_^−^) ions in postsynaptic cells. As a consequence, the inward chloride conductance increases, leading to hyperpolarization of the neural membrane and causing a rapid inhibitory effect and a central nervous system (CNS) depression [1,2,3].

The receptors also comprise specific allosteric sites through which the GABA-signaling can be modulated indirectly with allosteric modulators such as barbiturates, benzodiazepines, neurosteroids, and anesthetics. They bind on different sites on the receptor and exert their effects by causing conformational changes in the receptor, increasing the conductance of Cl^−^ ions, leading to inhibitory effects [8,9,10]. Regarding benzodiazepines, the binding site on the GABA_A_ receptor has been identified. Flumazenil is best known as a competitive antagonist at the benzodiazepine-binding site on the GABA_A_ receptor. It has long-standing clinical use as an emergency treatment for benzodiazepine overdose [11,12].

GABA_B_ receptors are G-protein coupled receptors, that increase potassium conductance, hyperpolarize the neurons, decrease calcium entry, and exert a slow inhibitory effect on the presynaptic release of other transmitters [1,2,3]. A variety of disorders are linked to the dysfunction of the GABA_B_ receptors, including memory alteration, anxiety, depression, alcohol addiction, and cancer. The GABA_B_ agonists (e.g., baclofen) are not used in epilepsy because they can aggravate absence (petit-mal) seizures [13].

While brief activation of GABA_A_ receptors leads to Cl^−^ influx-dependent hyperpolarization, prolonged receptor involvement leads to a strong HCO_3_^−^-efflux-dependent depolarization. This is a favorable factor for neuronal instability; as mentioned before, GABA_A_ receptors are permeable to both chloride and bicarbonate ions. The influence of this HCO_3_^−^-dependent depolarization can be mitigated by carbonic anhydrase (CA) inhibition via GABA_A_ receptors.

The presence of epileptic conditions can increase CA levels in the brain with alkalosis, which increases neural excitability, while acidosis has the opposite effect. There is clinical evidence that the enhancement of CO_2_ concentration results in better seizure control, while low CO_2_ level is linked with higher seizure risk. The inhibition of brain CA results in increased CO_2_ concentration and a positive outcome in epilepsy management [14,15,16].

Quinazolin-4(3H)-one and their derivatives are an important class of heterocyclic compounds with anticonvulsant and CNS depressant activities [6,17,18]. Methaqualone is a controversial quinazolin-4(3H)-one that was used in the 1960s and the 1970s as a sedative-hypnotic. It was considered safer than barbiturates and benzodiazepines, offering a faster induction of sleep with fewer hangover effects and side effects. However, it was withdrawn from the market due to its illicit use as a recreational drug. Studies conducted on this compound have indicated that it is a positive allosteric modulator (PAM) of the GABA_A_ receptor with good anticonvulsant properties. For example, methaqualone did not interact with benzodiazepines, barbiturates, or neurosteroid binding sites in the GABA_A_ receptor. The compound acts through the transmembrane *β*^(+)^/*α*^(−)^ subunit interface of the receptor, possibly targeting a site overlapping with that of the general anesthetic etomidate [7].

Structure–activity relationship (SAR) studies have shown that in order to exert their antiepileptic activity, methaqualone and its analogues with the quinazolin-4(3H)-one structure must meet certain structural requirements for binding to the GABA_A_ receptor: the presence of quinazolin-4(3H)-one moiety as hydrophobic domain, N1 as an electron donor atom, and the presence of the carbonyl group as a hydrogen bonding site. The substituents from positions 2 and 3 are responsible especially for the pharmacokinetics and the anticonvulsant potency [19,20].

**Figure 1:**
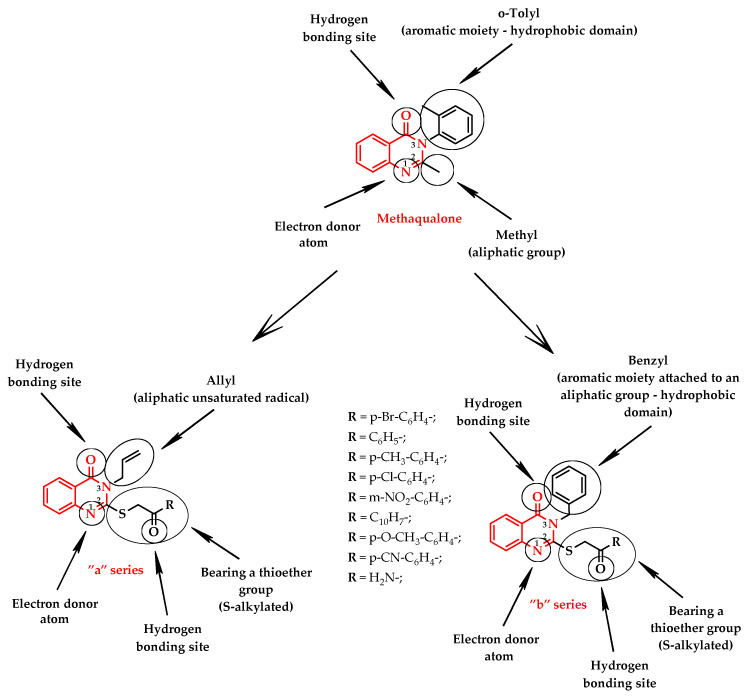
Structural design profile of the proposed anticonvulsant quinazolin−4−ones (“**a**” series and “**b**” series) by comparation with methaqualone structure.

Starting from these observations, our aim in the current study was the design and synthesis of new series of quinazolin-4(3H)-one derivatives with anticonvulsant potential, which target both the allosteric sites of the GABA_A_ receptor and the CA active center, by chemically modulating positions 2 and 3. For this purpose, in position 3 we introduced less rigid substituents (allyl and benzyl (“**a**” series and “**b**” series)), with the goal of achieving a deeper penetration into one of the hydrophobic pockets of the targets, aiming to obtain potent anticonvulsant agents (Figure 1). Meanwhile, the substituents in position 2 were various S-methyl-keto-aryl or S-acetamido fragments capable of coordinating the zinc ion from CA active center in a bidentate way, with the aim of obtaining an inhibitory effect of this enzyme [21,22,23].

In the present research, we describe the design, chemical synthesis, and in vivo anticonvulsant activity of two series of quinazolin-4(3H)-one derivatives as potential positive allosteric modulators (PAM) of the GABA_A_ receptor and inhibitors of carbonic anhydrase II.

## 2. Results and Discussion

### 2.1. Chemical Synthesis

**Scheme 1:**
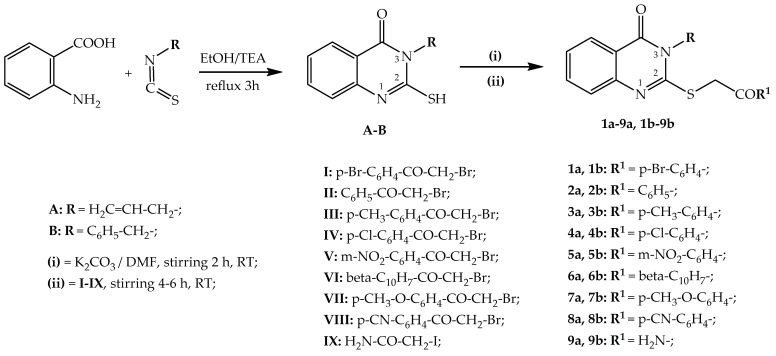
Chemical synthesis of the compounds **1a**–**9a** and **1b**–**9b**.

A total of 18 compounds, **1a**–**9a** and **1b**–**9b**, grouped in two series, “**a**” and “**b**”, were synthesized by the condensation of 3-allyl-2-mercaptoquinazolin-4(3H)-one **A** or 3-benzyl-2-mercaptoquinazolin-4(3H)-one **B** with α-bromoacetophenone derivatives **1**–**8**, β-(α-bromoacetyl)-naphthalene **6** and iodoacetamide **9**, in good yields. All stages of the chemical synthesis are shown in Scheme 1.

The spectral data were analyzed, and the results were in accordance with the proposed structures. The IR spectra for all the synthesized compounds, **1a**–**9a** and **1b**–**9b**, revealed the desired signals.

For compounds **1a**–**9a** and **1b**–**9b**, two strong C=O stretching signals were found between 1598.70–1698.96 cm^−1^: one from the quinazolin-4(3H)-one heterocycle and another one from the α-bromoacetophenone derivatives, β-(α-bromoacetyl)-naphthalene or iodoacetamide. The C=N stretching, from the quinazolin-4(3H)-one heterocycle, had a specific signal between 1539.40–1550.49 cm^−1^. Through all the identified signals, it has been proven that the condensation took place successfully.

In the MS of the synthesized compounds, **1a**–**9a** and **1b**–**9b,** the identified molecular mass peaks were in accordance with the proposed structures.

In the ^1^H-NMR spectra of the compounds **1a**–**9a** and **1b**–**9b**, all protons’ expected signals were identified, with the corresponding multiplicity. In the ^13^C-NMR spectra of compounds, the expected carbon atoms signals were identified and the signals were in the expected region of each spectrum.

The graphic depictions of the IR, MS, and NMR recorded spectra for compounds **1a**–**9a** and **1b**–**9b**, are provided in the Supplementary Materials (Figures S1–S70).

### 2.2. In Vivo Anticonvulsant Activity Evaluation

#### 2.2.1. Pentylenetetrazole (PTZ)-Induced Seizures

To assess the anticonvulsant properties of the synthesized compounds and their possible mechanisms of action, which might function similarly to conventional drugs such as diazepam and phenobarbital, the PTZ-induced seizures model was conducted. This model is specific for drugs that influence GABAergic transmission.

**Figure 2:**
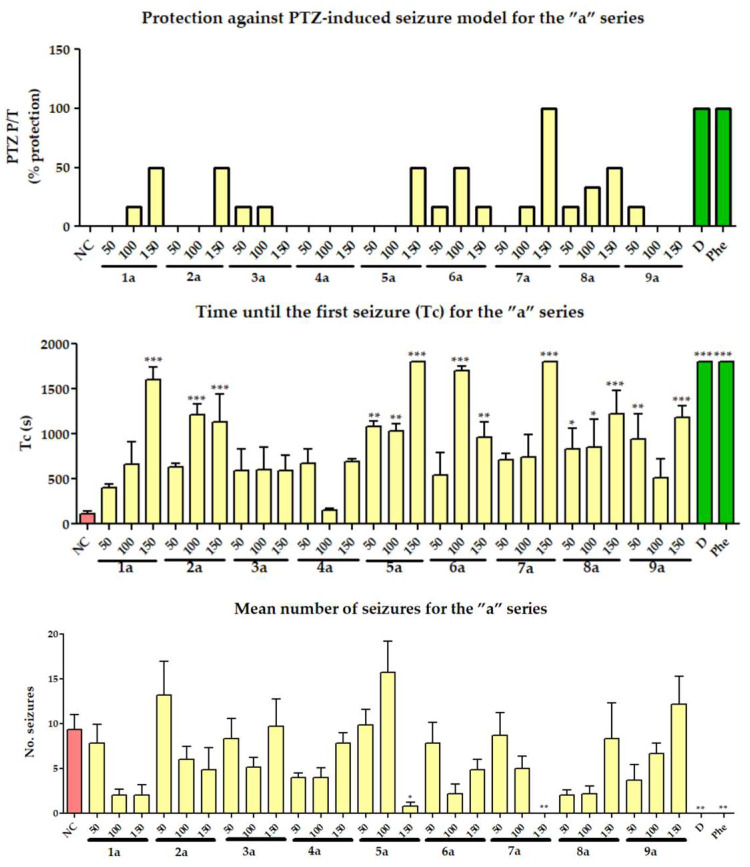
Protection against PTZ-induced seizure model, time until the first seizure and mean number of seizures, for the compounds of the “**a**” series, at all three doses, in the first 30 min after PTZ administration. **NC**—negative control (solvent); **D**—diazepam; **Phe**—phenobarbital; **PTZ P/T**—pentylenetetrazole; (**P**)—number of mice protected (no seizures)/(**T**)—number of mice tested; **Tc**—time (seconds) until the first seizure ± **SEM** (standard error of mean); **No. seizures**—mean number of seizures in the first 30 min/animal lot ± **SEM** (standard error of mean); *** *p* < 0.001; ** *p* < 0.01; * *p* < 0.05; statistically significant compared to negative control group.

**Figure 3:**
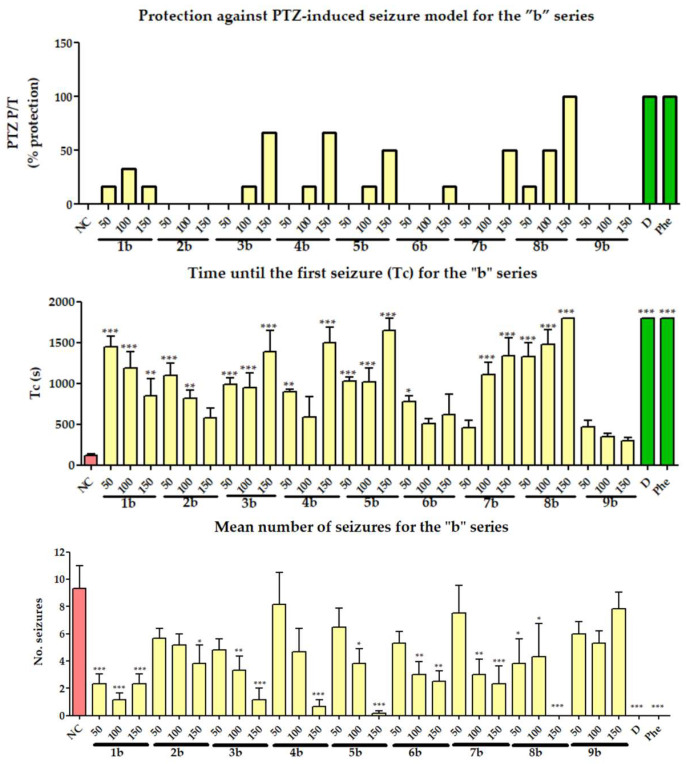
Protection against PTZ-induced seizure model, time until the first seizure and mean number of seizures, for the compounds of the “**b**” series, at all three doses, in the first 30 min after PTZ administration. **NC**—negative control (solvent).; **D**—diazepam.; **Phe**—phenobarbital; **PTZ P/T**—pentylenetetrazole; (**P**)—number of mice protected (no seizures)/(**T**)—number of mice tested; **Tc**—time (seconds) until the first seizure ± **SEM** (standard error of mean); **No. seizures**—mean number of seizures in the first 30 min/animal lot ± **SEM** (standard error of mean); *** *p* < 0.001; ** *p* < 0.01; * *p* < 0.05; statistically significant compared to negative control group.

From the analysis of the obtained results, it was observed that most of the compounds exhibit anticonvulsant properties. However, this activity was not consistently correlated with the administered dose for all compounds (Figure 2 and Figure 3). During the testing of the compounds, the highest effective dose for most of them was 150 mg/kg, with reduction in the number of seizures and a protection level ranging between 16.67% and 100% for compounds from both series. In each series, there are inactive compounds that were identified at all three tested doses: **4a** (R^1^ = p-Cl-C_6_H_4_-) and the compounds **2b** (R^1^ = C_6_H_5_-) and **9b** (R^1^ = H_2_N-).

Two of the tested compounds, **7a** (R^1^ = p-CH_3_-O-C_6_H_4_-) and **8b** (R^1^ = p-CN-C_6_H_4_-), provided 100% protection against the PTZ-induced seizures. The reference substances, as expected, ensured 100% protection in the experimental model. Regarding the latency of the first convulsion, the results indicated that it increases with the dose, reaching maximum protection and varying between 153 and 1800 s (Figure 2 and Figure 3).

The obtained results for compounds **1a**–**9a** and **1b**–**9b** are also provided in the Supplementary Materials (Tables S1 and S2).

From the analysis of the obtained results, a series of conclusions can be drawn regarding the relationship between the chemical structure of the compounds obtained and the anticonvulsant potential. The SAR study revealed that compounds substituted at position 3 with an allyl radical (“**a**” series) generally exhibited higher activity compared to the substituted benzyl compounds (“**b**” series), particularly when the substituent at position 2 of the quinazolin-4-one presents an electron-donating substituent (EDG) and a bulkier nucleus (e.g., **1a** > **1b** (R^1^ = p-Br-C_6_H_4_-), **2a** > **2b** (R^1^ = C_6_H_5_-), **7a** > **7b** (R^1^ = p-CH_3_-O-C_6_H_4_-)). However, the presence of an electron-withdrawing substituent (EWG) leads to a change in the activity ratio, with compounds from the “**b**” series being more active than those from the “**a**” series (e.g., **8a** < **8b** (R^1^ = p-CN-C_6_H_4_-)). The effect is similar for m-nitro-substituted (**5a**, **5b**) and for the beta-naphthyl-substituted (**6a**, **6b**). The lowest anticonvulsant activity is found in compounds with an unsubstituted amide group in position 3 (**9a**, **9b**). The way in which the substituents influence the anticonvulsant potential (degree of protection, number of seizures, time until the first seizure) argues for the importance of the existence of some substituents that increase the electron density on the nitrogen of the quinazolin-4-one structure in the basic pharmacophore.

#### 2.2.2. Flumazenil Antagonism Assay

**Figure 4:**
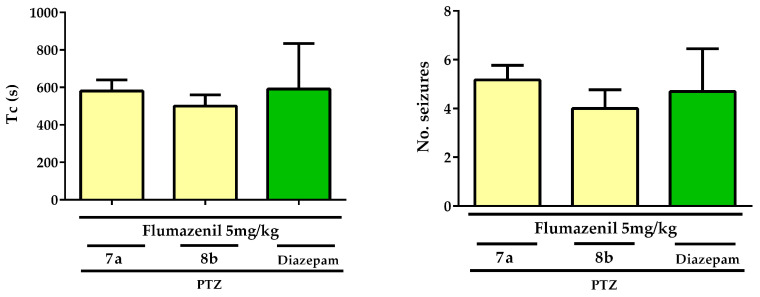
Results obtained in flumazenil antagonism assay for the compounds **7a** and **8b**. **Tc** (**s**)—time (seconds) until the first seizure (mean) ± **SEM** (standard error of mean; **No. seizures**—mean number of seizures in the first 30 min/animal lot ± **SEM** (standard error of mean).

For this assay, the most active compounds in the PTZ-induced seizure test were chosen. From the “**a**” series, the chosen compound was **7a**, and from the “**b**” series, there was **8b,** due to their protection against PTZ-induced seizures, latency until the first seizure, and number of seizures (Figure 4).

Flumazenil is used in research studies, due to the antagonism on the GABA_A_ receptor of benzodiazepine binding site at the extracellular α^(+)^/γ^(−)^ subunits interface [24]. The results of this study were similar to diazepam and showed that none of the tested substances exhibited protective effects against PTZ-induced seizures after the administration of flumazenil. This suggests an anticonvulsant mechanism of action as positive allosteric modulators of the GABA_A_ receptor.

The obtained results are also provided in the Supplementary Materials (Table S3).

### 2.3. In Silico Studies

#### 2.3.1. LogP Prediction

It was found that the compounds substituted in position 3 of quinazolinone with benzyl (“**b**” series) have a higher lipophilicity when compared to the ones in the “**a**” series, substituted in position 3 of quinazolinone with allyl. This would give the “**b**” series compounds a better penetrability through the biological membranes to reach their target.

The highest lipophilicity was identified in compound **7b** (R^1^ = p-CH_3_-O-C_6_H_4_-), but the compound **8b** (R^1^ = p-CN-C_6_H_4_-) exhibited the most potent anticonvulsant activity, also possessing a Log*p* value > 3.

The compounds with the lowest lipophilicity were identified to be **9a** and **9b** (R^1^ = H_2_N-) exhibiting the lowest anticonvulsant activity in the in vivo assays, which can be correlated with a lower penetrability through membranes and a diminished interaction with the target (Tables S1a and S2 in Supplementary Materials).

#### 2.3.2. Molecular Docking

The newly synthesized compounds were targeted to the benzodiazepine binding site in the GABA_A_ receptor and the active site of carbonic anhydrase II. The results of the molecular docking to the GABA_A_ receptor and to the carbonic anhydrase II are presented in Table 1.

**Figure 5:**
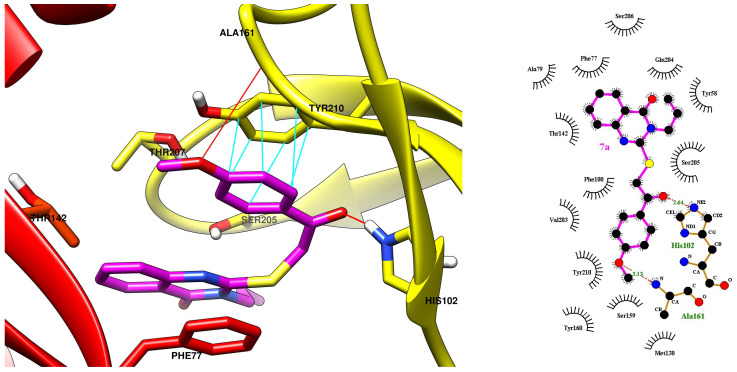
The best binding pose of compound **7a** in the benzodiazepine binding site of the GABA_A_ receptor (**left**). The γ2 subunit is depicted in red ribbons, while the α1 subunit is depicted in yellow ribbons. The oxygen atom from the ketone of compound **7a** is involved in the hydrogen bond as an acceptor with the sidechain of His102 (red line), while the oxygen atom from the ether of **7a** is involved in a hydrogen bond as acceptor with the nitrogen atom of Ala161 (red line). The benzene ring of **7a** is involved in a π-π stacking interaction with Tyr210 sidechain (cyan lines). The 2D representation of the interactions of **7a** in the benzodiazepine binding site of GABA_A_ receptor is depicted on the (**right**).

**Figure 6:**
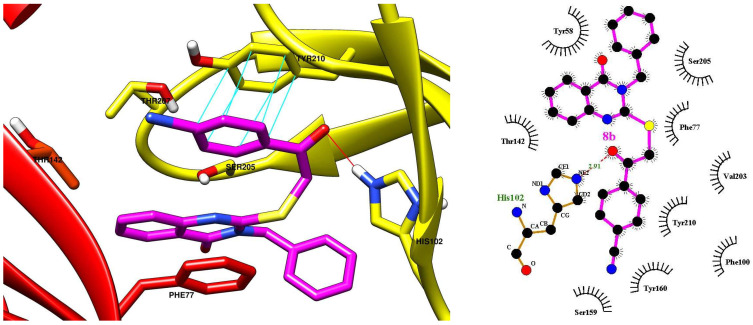
The best binding pose of compound **8b** in the benzodiazepine binding site of the GABA_A_ receptor (**left**). The γ2 subunit is depicted in red ribbons, while the α1 subunit is depicted in yellow ribbons. The oxygen atom from the ketone of compound **8b** is involved in the hydrogen bond as an acceptor with the sidechain of His102 (red line). The benzene ring of **8b** is involved in a π-π stacking interaction with Tyr210 sidechain (cyan lines). The 2D representation of the interactions of **8b** in the benzodiazepine binding site of the GABA_A_ receptor is depicted on the (**right**).

**Figure 7:**
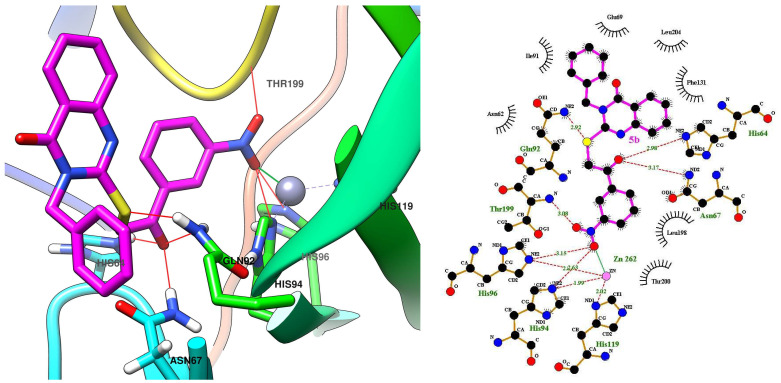
The best binding pose of compound **5b** in the active site of carbonic anhydrase II (**left**). One of the oxygen atoms from the nitro group of **5b** is involved in an electrostatic interaction with the positively charged zinc atom from the active site of carbonic anhydrase II (green line) or with the sidechain of His94 or His96 in hydrogen bonds (red lines), while the other oxygen from the nitro group is predicted to be involved in a hydrogen bond with the nitrogen atom of Thr199 (red line). The ketone of compound **5b** is predicted to interact with hydrogen bonds as acceptor with His64, Asn67, or Gln92 sidechains (red lines). A supplementary hydrogen bond is supposed to occur between Gln92 sidechain with the sulfur atom from the thioether. The 2D representation of the interactions of **5b** in the active site of carbonic anhydrase II is depicted on the (**right**).

Figure 5 illustrates the binding pose of compound **7a** in the benzodiazepine binding site of the GABA_A_ receptor. Figure 6 presents the binding pose of compound **8b** in the benzodiazepine site of the GABA_A_ receptor. Figure 7 describes the binding pose of compound **5b** in the active site of carbonic anhydrase II.

All the compounds were illustrated in their best binding pose in the benzodiazepine binding site of the GABA_A_ receptor in Figures S71–S79 in Supplementary Materials.

The affinity of the compounds for the benzodiazepine site in the GABA_A_ receptor does not follow a trend related to the way the molecules are substituted. On average, viewed globally over the entire series, the compounds from the “**b**” series had a higher binding affinity than those from the “**a**” series, but a major influence is also the second substituent from the thioether moiety. For example, the allylic derivative **7a** had a higher binding affinity for the GABA_A_ receptor than the benzylic one (**7b**).

From the data sets generated by the two software, the energies whose values were more promising than a threshold represented by the value of the first quartile of each series of energies generated by both software were extracted (Q1_AutoDock_ = 10.69 kcal/mol; Q1_AutoDock vina_ = 10.11 kcal/mol). The compounds matching the respective conditions were: **5a**, **7a**, **5b,** and **8b**. This protocol was applied to eliminate potential false positive results and to reduce the number of mice needed to confirm the GABAergic mechanism of these compounds by using the flumazenil antidote assay.

In humans, more than sixteen isoforms of CA were described, which have different catalytic activities and various subcellular and tissue distribution. It has been reported that Cas II, VII, and XIV are implicated in epilepsy. The CAs belong to the family of metalloenzymes with a zinc ion in the active center, essential for catalytic processes. They catalyze a reaction that takes place in all living organisms, the reversible hydration of carbon dioxide into bicarbonate and protons (CO_2_ + H_2_O ⇌ HCO_3_^−^ +H^+^). The enzyme is active in its basic form when a hydroxyl group is bound to Zn^2+^ (E-Zn^2+^-OH^−^). The resulting complex is responsible for the nucleophile attack on the CO_2_ molecule, located in the hydrophobic region beside the zinc ion, leading to the formation of HCO_3_^−^. Then, the bicarbonate ion is released in solution and replaced by a water molecule (E-Zn^2+^-H_2_O), generating the inactive form. To activate the enzyme, a new transfer of protons from the water complex is needed. All the reactions are shown in the Equation (1) [14,15].

E-Zn^2+^-OH^−^ + CO_2_ ⇌ E-Zn(OH^−^)CO_2_^−^ ⇌ E-Zn^2+^-HCO_3_^−^ ⇌ E-Zn^2+^-H_2_O + HCO_3_^−^ E-Zn^2+^-H_2_O ⇌ E-Zn^2++^-OH^−^ + H^+^, (1)

Using the top binding pose of each compound generated by AutoDock Tools 1.5.6, their complexes with carbonic anhydrase II were constructed, solvated, neutralized, and minimized according to the presented protocol. After 100 ns of simulation, the evolution of the systems was visually analyzed using VMD by processing the resulted trajectory files. Visual inspection of the evolution of the position of the ligand in the active site of carbonic anhydrase showed that the predicted complexes have a high degree of instability.

Regarding the carbonic anhydrase binding energies, it is observed that these are small, lower than the GABA_A_ binding energies (Table 1). The affinity of the compounds for the carbonic anhydrase II is strongly dependent to the way that the compounds are substituted. For any type of substitution on the thioether moiety, the compound from “**b**” series have a higher binding affinity than its analog from “**a**” series.

From the datasets generated by the two software, the energies whose values were more promising than a threshold represented by the value of the first quartile of each series of energies generated by both software were extracted (Q1_AutoDock_ = −8.01 kcal/mol; Q1_AutoDock vina_ = −7.24 kcal/mol). The compounds matching the respective conditions were: **3b**, **4b**, **5b, 7b,** and **8b**. The predicted carbonic anhydrase II-compound complexes were subjected to a further molecular dynamics study, in order to evaluate the stability of the predicted poses, to investigate their mechanism as potential carbonic anhydrase II inhibitors.

**Figure 8:**
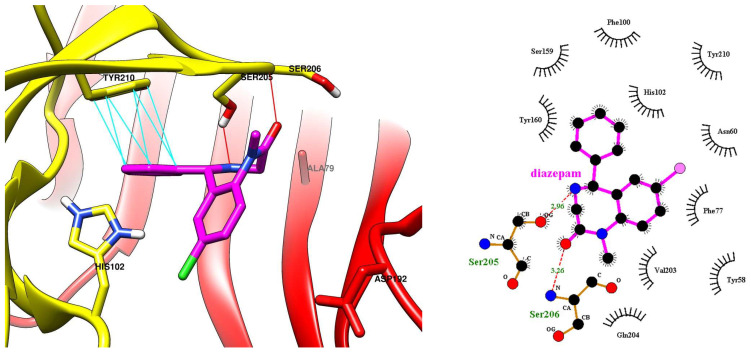
The binding site of diazepam in the benzodiazepine binding site (α _+_ γ^−^ interface) of the GABA_A_ receptor (**left**). The γ2 subunit is depicted in red ribbons, while the α1 subunit is depicted in yellow ribbons [24,25]. Diazepam is interacting as a hydrogen bond acceptor both via the N4 atom from the sidechain (red line) of Ser205 and with the nitrogen atom from Ser206. The benzene ring of diazepam is involved in a π-π stacking interaction with Tyr210 sidechain (cyan lines). The 2D representation of the interactions of diazepam in the benzodiazepine binding site of GABA_A_ receptor is depicted on the (**right**).

According to the literature, diazepam is a positive allosteric modulator on the GABA_A_ receptor for the anticonvulsant activity. Its interaction with the GABA_A_ receptor is depicted in Figure 8 [25,26].

Also, the anticonvulsant activity of methaqualone is suggested through the positive allosteric modulator on the GABA_A_ receptor too. It interacts through the hydrogen bond between the N1 atom of the quinazolin-4-one heterocycle and the Ile218 of the GABA_A_ receptor benzodiazepine binding site. The carbonyl group from the heterocycle forms a hydrogen bound with Lys215 subunit. The amino acids are acting as a gate for the ligand entrance to the GABA_A_ receptor [27].

The compounds from the “**b**” series could have a higher binding affinity for the GABA_A_ receptor due to being positive allosteric modulators too, by their resemblance to methaqualone; in position 2, it also has a hydrophobic moiety.

#### 2.3.3. Molecular Dynamics Simulation

Due to the fact that molecular docking can generate false positive results, it is necessary to employ supplementary techniques to filter the results. The stability of the ligand–carbonic anhydrase II predicted complexes was evaluated to find if the most promising compounds could act as potential carbonic anhydrase II inhibitors.

The compounds chosen for the molecular dynamics simulation were those with the highest affinity in the molecular docking study performed on the carbonic anhydrase II (**3b**, **4b**, **5b**, **7b,** and **8b**). Since the most promising compounds in the in vivo assay were **5a**, **7a**, **5b,** and **8b**, in the molecular dynamics simulation, compounds **5a** and **7a** were also evaluated, to assess the possibility that they could have a supplementary mechanism, the inhibition of the carbonic anhydrase II, not identified in the molecular dockings.

The aim of the present simulations of the ligands–carbonic anhydrase complexes was to evaluate their stability in time. High instability was identified for the studied complexes. It was concluded that the results of the molecular docking study against carbonic anhydrase were false-positives and the anticonvulsant mechanism of the compounds is independent of this enzyme. Thus, more complex calculations derived from the molecular dynamics simulations (such as the calculation of the binding free energy) were considered unnecessary and were not performed.

The spatial movement of the ligands was computed and depicted in charts as the root mean square deviation (RMSD) of the coordinates of the ligands in Figure S80 in the Supplementary Materials.

In Figure S80, it can be seen the ligands have wide, quite free movements in the active site of the enzyme. In the first period of the simulation, all ligands moved from their initial position, and during the evolution of the simulation, two behaviors of movement were identified according to the substitution of the compounds. Compounds N3-benzyl derived and substituted with an electron withdrawing group **4b** (R^1^ = p-Cl-C_6_H_4_-), **5b** (R^1^ = m-NO_2_-C_6_H_4_-), and **8b** (R^1^ = p-CN-C_6_H_4_-) have the lowest RMSD from the current series in the first half of the molecular dynamics simulation. After approximately 60 ns, all three ligands presented before showed an unstable position compared to their position during the plateau stage of the simulation, showing that the complexes with the carbonic anhydrase II are not stable over time.

Since the ligands leave the active site of the enzyme, we did not perform other analysis on the evolution of the complexes over time (root mean square deviation of the backbone of the protein, root mean square fluctuation, types of ligand–protein interactions, hydrogen bonds, etc.), because they would not make sense in this context.

As a result of the molecular dynamics simulation performed against carbonic anhydrase II, which showed that the interaction between the present compounds and the enzyme is weak and not stable in time, we repeated the molecular docking of the ligands against more isoforms of carbonic anhydrase (I, VI, IX, and XII). The stability of the complexes of the ligands from the first quartile of affinity on the enzymes was evaluated in a molecular dynamics study. It was identified that none of the compounds would have strong and persistent interactions with the studied isoforms of carbonic anhydrase (data not presented). Therefore, we consider that these compounds do not act as carbonic anhydrase inhibitors, even if they show some structural similarities with compounds previously reported in the literature that possess this activity.

The tested compounds, **1a**–**9a** and **1b**–**9b**, demonstrated an affinity for the benzodiazepine binding site within the structure of the GABA_A_ receptor, acting as positive allosteric modulators at this level. However, the instability over time of the ligand-active site of carbonic anhydrase II complexes, disproves the hypothesis of a mechanism of action involving the inhibition of CA.

## 3. Materials and Methods

### 3.1. Chemistry

The reagents used for synthesis, purification, and structural analysis were purchased from local suppliers, being produced by Merck KGaA (Darmstadt, Germany).

The purity of the starting materials and the final products was preliminarily checked through thin-layer chromatography (TLC).

The structure and purity of tested compounds was confirmed by recording and analysis of the IR, LC-MS, ^1^H-NMR, and ^13^C-NMR spectra.

Melting points were measured by using MPM-H1 (Schorpp Gerätetechnik, Überlingen, Germany), a melting point device, based on the glass capillary method.

The IR spectra were recorded by using an FT/IR 6100 spectrometer (Jasco, Cremella, Italy), under vacuum, in KBr pellets.

To record the MS spectra, an Agilent Ion Trap SL mass spectrometer instrument (Agilent Technologies, Santa Clara, CA, USA) was used in positive ionization mode for the final compounds **1a**–**9a** and **1b**–**9b**.

For the ^1^H-NMR and ^13^C-NMR spectra, in dimethylsulfoxide-*d*_6_ (DMSO-*d*_6_), an Avance NMR spectrometer (Bruker, Karlsruhe, Germany) was used. The calibration of the spectrometer was performed using tetramethylsilane. To identify the multiplicity of the signals in the ^1^H-NMR spectra, the following abbreviations for peak patterns were utilized: *s*-singlet, *d*-doublet, *dd*-double doublet, *t*-triplet, *dt*-doublet of triplets, *td*-triplet of doublets, and *m*-multiplet, respectively. In order to describe the location of the atom in a specific region of the molecule, for the signals given by the hydrogen or carbon atoms, some abbreviations were used: *Q*—uinazolin-4(3H)-one, *Bz*—benzyl, and *Ar*—substituted phenyl.

#### 3.1.1. Synthesis of Intermediate Compounds **A** and **B**

The intermediate compounds **A** and **B** were previously reported by our group [28,29].

#### 3.1.2. Synthesis of Compounds **1a**–**9a**

A total of 3 mmol (654 mg) of 3-allyl-2-mercaptoquinazolin-4(3H)-one **A** and 3.5 mmol (483 mg) of potassium carbonate were added in 5 mL of dimethylformamide (DMF), in a glass flask. The mixture was stirred for 2 h at room temperature, and 3 mmol of α-bromoacetophenone derivatives **1**, **2**, **3**, **4**, **5**, **7**, **8**, β-(α-bromoacetyl)-naphthalene **6**, or iodoacetamide **9** were added. The mixture was stirred for another 4–6 h at room temperature. The resulting precipitates were obtained by pouring on water and neutralization with a dropwise of 5% hydrochloric acid solution. The final precipitates were filtered under a vacuum, dried and crystallized from dioxane. Compounds **1a**, **3a**, and **4a** were reported in the literature by using a resembling protocol [30]. Using a different protocol, the compounds **2a**, **3a**, and **4a** were reported in the literature [31].

*3-allyl-2-((2-(4-bromophenyl)-2-oxoethyl)thio)quinazolin-4(3H)-one* (**1a**): white solid; mp = 147 °C; yield = 73.42%; FT IR (KBr) ν_max_cm^−1^: 1678.25, 1607.38 (str C=O), 1547.11 (C=N), 695.69 (C-Br); MS: *m*/*z* = 415.4 [M+H^+^]; ^1^H-NMR (DMSO-*d*_6_, 500 MHz) δ: 8.066–8.043 (m, 3H, 1Q and 2Ar), 7.831 (d, 2H, Ar, *J* = 8.50 Hz), 7.681 (dt, 1H, Q, *J* = 7.50 and 1.50 Hz), 7.411 (dt, 1H, Q, *J* = 7.50 and 1.50 Hz), 7.011 (d, 1H, Q, *J* = 8.00 Hz), 5.999–5.943 (m, 1H, -CH=), 5.260 (dd, 1H, =CH_2_, *J* = 10.50 and 1.00 Hz), 5.176 (dd, 1H, =CH_2_, *J* = 17.25 and 1.00 Hz), 4.851 (s, 2H, -CH_2_-), 4.768 (d, 2H, -CH_2_-, *J* = 5.00 Hz); ^13^C-NMR (DMSO-*d*_6_, 125 MHz) δ: 192.934 (C=O), 160.136 (C=O), 155.985 (C=N), 146.452 (Q), 135.442 (Ar), 134.714 (Q), 131.830 (Ar), 131.236 (-CH=), 130.263 (Ar), 127.498 (Ar-Br), 126.462 (Q), 125.979 (Q), 125.342 (Q), 118.518 (=CH_2_), 117.580 (Q), 46.054 (-CH_2_-), 38.740 (-CH_2_-).

*3-allyl-2-((2-oxo-2-phenylethyl)thio)quinazolin-4(3H)-one* (**2a**): white solid; mp = 119 °C; yield = 62.47%; FT IR (KBr) ν_max_cm^−1^: 1694.16, 1675.84 (str C=O), 1548.56 (C=N); MS: *m*/*z* = 337.20 [M+H^+^]; ^1^H-NMR (DMSO-*d*_6_, 500 MHz) δ: 8.129–8.113 (m, 2H, Ar), 8.042 (dd, 1H, Q, *J* = 7.75 and 1.50 Hz), 7.725 (dt, 1H, Q, *J* = 7.25 and 1.25 Hz), 7.659 (dt, 2H, Ar, *J* = 7.50 and 1.25 Hz), 7.611 (t, 2H, Ar, *J* = 7.75 Hz), 7.397 (t, 1H, Q, *J* = 7.75 Hz), 6.993 (d, 1H, Q, *J* = 8.5 Hz), 6.000–5.935 (m, 1 H, -CH=), 5.272 (dd, 1H, -CH_2_-, *J* = 1.50 Hz), 5.251 (dd, 2H, -CH_2_-, *J* = 1.50 Hz), 4.874 (s, 2H, -CH_2_-), 4.773–4.763 (m, 2H, -CH_2_-); ^13^C-NMR (DMSO-*d*_6_, 125 MHz) δ: 193.557 (C=O), 160.157 (C=O), 156.055 (C=N), 146.487 (Q), 136.422 (Ar), 134.630 (Ar), 133.419 (Q), 131.257 (-CH=), 128.744 (Ar), 128.247 (Ar), 126.441 (Q), 125.916 (Q), 125.342 (Ar), 118.532 (=CH_2_), 117.601 (Q), 46.040 (-CH_2_-), 38.866 (-CH_2_-).

*3-allyl-2-((2-oxo-2-(p-tolyl)ethyl)thio)quinazolin-4(3H)-one* (**3a**): white solid; mp = 120 °C; yield = 67.45%; FT IR (KBr) ν_max_cm^−1^: 1684.03, 1680.18 (str C=O), 1544.70 (C=N); MS: *m*/*z* = 351.3 [M+H^+^]; ^1^H-NMR (DMSO-*d*_6_, 500 MHz) δ: 8.046 (dd, 1H, Q, *J* = 8.00 and 1.00 Hz), 8.017 (m, 1H, Ar, *J* = 8.00 Hz), 7.675 (td, 1H, Q, *J* = 7.75 and 1.25 Hz), 7.419–7.387 (m, 3H, 1Q and 2Ar), 7.051 (d, 1H, Ar, *J* = 9.00 Hz), 6.009–5.934 (m, 1H, -CH=), 5.259 (dd, 1H, =CH_2_, *J* = 1.50 Hz), 5.182 (dd, 1H, =CH_2_, *J* = 1.50 Hz), 4.852 (s, 2H, -CH_2_-), 4.774–4.764 (m, 2H, -CH_2_-), 2.432 (s, 1 H, -CH_3_); ^13^C-NMR (DMSO-*d*_6_, 125 MHz) δ: 192.906 (C=O), 160.164 (C=O), 156.062 (C=N), 146.501 (Q), 143.869 (Ar-CH_3_), 134.637 (Q), 133.832 (Ar), 131.264 (-CH=), 129.276 (Ar), 128.373 (Ar), 126.427 (Q), 125.895 (Q), 125.419 (Q), 118.525 (=CH_2_), 117.580 (Q), 46.005 (-CH_2_-), 38.880 (-CH_2_-), 21.193 (-CH_3_-).

*3-allyl-2-((2-(4-chlorophenyl)-2-oxoethyl)thio)quinazolin-4(3H)-one* (**4a**): white solid; mp = 137 °C; yield = 72.55%; FT IR (KBr) ν_max_cm^−1^: 1681.62, 1649.80 (str C=O), 1549.52 (C=N), 777.17 (C-Cl); MS: *m*/*z* = 371.2 [M+H^+^]; ^1^H-NMR (DMSO-*d*_6_, 500 MHz) δ: 8.133 (d, 2H, 2Ar, *J* = 8.50 Hz), 8.043 (dd, 1H, Q, *J* = 8.00 and 1.00 Hz), 7.690-7.666 (m, 3H, 1 Q and 2 Ar), 7.405 (t, 1H, Q, *J* = 7.50 Hz), 6.999 (d, 1H, Q, *J* = 7.50 Hz), 6.004–5.938 (m, 1H, -CH=), 5.255 (dd, 1H, -CH_2_-, *J* = 1.00 Hz), 5.171 (dd, 1H, -CH_2_-, *J* = 1.00 Hz), 4.851 (s, 2H, -CH_2_-), 4.763 (m, 2H, -CH_2_-); ^13^C-NMR (DMSO-*d*_6_, 125 MHz) δ: 192.731 (C=O), 160.143 (C=O), 156.006 (C=N), 146.459 (Q), 138.326 (Ar-Cl), 135.127 (Ar), 134.721 (Q), 131.243 (-CH=), 130.186 (Ar), 128.884 (Ar), 126.469 (Q), 125.979 (Q), 125.342 (Q), 118.525 (=CH_2_), 117.587 (Q), 46.061 (-CH_2_-), 38.754 (-CH_2_-).

*3-allyl-2-((2-(3-nitrophenyl)-2-oxoethyl)thio)quinazolin-4(3H)-one* (**5a**): white solid; mp = 148 °C; yield = 69.72%; FT IR (KBr) ν_max_cm^−1^: 1689.34, 1675.84 (str C=O), 1550.49 (C=N), 1530.24 (C-NO_2_); MS: *m*/*z* = 382.1 [M+H^+^]; ^1^H-NMR (DMSO-*d*_6_, 500 MHz) δ: 8.807 (t, 1H, Ar, *J* = 9.00 Hz), 8.563-8.542 (m, 2H, Ar), 8.039 (dd, 1H, Q, *J* = 7.75 and 1.50 Hz), 7.926 (t, 1H, Ar, *J* = 8.00 Hz), 7.653 (td, 1H, Q, *J* = 7.50 and 1.25 Hz), 7.400 (t, 1H, Q, *J* = 7.75 Hz), 6.948 (d, 1H, Q, *J* = 7.50 Hz), 6.008–5.932 (m, 1H, -CH=), 5.257 (dd, 1H, =CH_2_, *J* = 1.00 Hz), 5.168 (dd, 1H, =CH_2_, *J* = 1.00 Hz), 4.929 (s, 1H, -CH_2_-), 4.770–4.760 (m, 1H, -CH_2_-); ^13^C-NMR (DMSO-*d*_6_, 125 MHz) δ: 192.696 (C=O), 160.087 (C=O), 155.908 (C=N), 146.382 (Ar-NO_2_), 146.006 (Q), 137.724 (Ar), 134.707 (Ar), 134.441 (Q), 131.208 (-CH=), 130.627 (Ar), 127.561 (Ar), 126.476 (Q), 126.007 (Q), 125.230 (Q), 122.570 (Ar), 118.518 (=CH_2_), 117.559 (Q), 46.103 (-CH_2_-), 38.684 (-CH_2_-).

*3-allyl-2-((2-(naphthalen-2-yl)-2-oxoethyl)thio)quinazolin-4(3H)-one* (**6a**): white solid; mp = 155 °C; yield = 74.29%; FT IR (KBr) ν_max_cm^−1^: 1679.69, 1646.91 (str C=O), 1550.49 (C=N); MS: *m*/*z* = 387.3 [M+H^+^]; ^1^H-NMR (DMSO-*d*_6_, 500 MHz) δ: 8.915 (s, 1H, Ar), 8.195 (d, 1H, Ar, *J* = 8.00 Hz), 8.107–8.037 (m, 4H, 1Q and 3Ar), 7.642-7.592 (m, 3H, 1Q and 2Ar), 7.401–7.370 (m, 1H, Q), 6.980 (d, 1H, Q, *J* = 8.00 Hz), 6.028–5.953 (m, 1H, -CH=), 5.268 (dd, 1H, =CH_2_, *J* = 1.00 Hz), 5.192 (dd, 1H, =CH_2_, *J* = 1.00 Hz), 5.028 (s, 2H, -CH_2_-), 4.800–4.789 (m, 2H, -CH_2_-); ^13^C-NMR (DMSO-*d*_6_, 125 MHz) δ: 193.417 (C=O), 160.192 (C=O), 156.125 (C=N), 146.515 (Q), 135.180 (Ar), 134.658 (Q), 133.671 (Ar), 132.159 (Ar), 131.292 (-CH=), 130.263 (Ar), 129.686 (Ar), 128.807 (Ar), 128.366 (Ar), 127.736 (Q), 126.469 (Q), 125.944 (Q), 125.377 (Ar), 123.823 (Ar), 121.231 (Ar), 118.546 (=CH_2_), 117.580 (Q), 46.068 (-CH_2_-), 38.950 (-CH_2_-).

*3-allyl-2-((2-(4-methoxyphenyl)-2-oxoethyl)thio)quinazolin-4(3H)-one* (**7a**): white solid; mp = 123 °C; yield = 72.12%; FT IR (KBr) ν_max_cm^−1^: 1679.69, 1647.39 (str C=O), 1545.67 (C=N), 1169.13 (C-O-C); MS: *m*/*z* = 367.2 [M+H^+^]; ^1^H-NMR (DMSO-*d*_6_, 500 MHz) δ: 8.120-8.090 (m, 2H, Ar), 8.058-8.039 (m, 1H, Q), 7.687 (td, 1H, Q, *J* = 7.75 and 1.25 Hz), 7.407 (td, 1H, Q, *J* = 7.50 and 0.75 Hz), 7.135–7.082 (m, 3H, 1Q and 2Ar), 6.011–5.935 (m, 1H, -CH=), 5.258 (dd, 2H, -CH_2_-, *J* = 1.50 Hz), 5.183 (dd, 2H, -CH_2_-, *J* = 1.50 Hz), 4.839 (s, 1H, -CH_2_-), 4.780-4.767 (m, 2H, -CH_2_-); ^13^C-NMR (DMSO-*d*_6_, 125 MHz) δ: 191.695 (C=O), 163.341 (Ar-O-CH_3_), 160.178 (C=O), 156.118 (C=N), 146.515 (Q), 134.651 (Q), 131.278 (-CH=), 130.668 (Ar), 129.129 (Q), 126.427 (Q), 125.895 (Q), 125.475 (Ar), 118.532 (=CH_2_), 117.506 (Q), 113.947 (Ar), 55.573 (-CH_3_), 45.977 (-CH_2_-), 38.698 (-CH_2_-).

*4-(2-((3-allyl-4-oxo-3,4-dihydroquinazolin-2-yl)thio)acetyl)benzonitrile* (**8a**): white solid; mp = 178 °C; yield = 70.34%; FT IR (KBr) ν_max_cm^−1^: 2234.13 (C≡N), 1693.19, 1681.14 (str C=O), 1539.40 (C=N); MS: *m*/*z* = 362.1 [M+H^+^]; ^1^H-NMR (DMSO-*d*_6_, 500 MHz) δ: 8.263 (d, 2H, Ar, *J* = 8.00 Hz), 8.100 (d, 2H, Ar, *J* = 8.50 Hz), 8.039 (d, 1H, Q, *J* = 8.00 Hz), 7.677 (t, 1H, Q, *J* = 7.50 Hz), 7.403 (t, 1H, Q, *J* = 7.50 Hz), 6.923 (d, 1H, Q, *J* = 8.50 Hz), 6.004–5.292 (m, 1H, -CH=), 5.258 (d, 1H, =CH_2_, *J* = 10.50 Hz), 5.169 (d, 1H, =CH_2_, *J* = 17.50 Hz), 4.878 (s, 2H, -CH_2_-), 4.759 (d, 2H, -CH_2_-, *J* = 5.00 Hz); ^13^C-NMR (DMSO-*d*_6_, 125 MHz) δ: 193.354 (C=O), 160.080 (C=O), 155.894 (C=N), 146.382 (Q), 139.754 (Ar), 134.714 (Q), 132.803 (Ar), 131.194 (-CH=), 128.828 (Ar), 126.455 (Q), 125.986 (Q), 125.223 (Q), 118.497 (=CH_2_), 118.119 (Ar-C≡N), 117.580 (Ar), 115.305 (Ar), 46.096 (-CH_2_-), 38.782 (-CH_2_-).

*2-((3-allyl-4-oxo-3,4-dihydroquinazolin-2-yl)thio)acetamide* (**9a**): white solid; mp = 189 °C; yield = 68.23%; FT IR (KBr) ν_max_cm^−1^: 1679.69, 1646.91 (str C=O), 1550.49 (C=N); MS: *m*/*z* = 276.1 [M+H^+^]; ^1^H-NMR (DMSO-*d*_6_, 500 MHz) δ: 8.086 (dd, 1H, Q, *J* = 8.00 and 1.50 Hz), 7.805 (dt, 1H, Q, *J* = 7.50 and 1.25 Hz), 7.686 (s, 1H, -NH_2_), 7.540 (d, 1H, Q, *J* = 8.00 Hz), 7.463 (td, 1H, Q, *J* = 7.50 and 1.50 Hz), 7.232 (s, 1H, -NH_2_), 5.985–5.910 (m, 1H, -CH=), 5.232 (dd, 1H, =CH_2_, *J* = 1.50 Hz), 5.157 (dd, 1H, =CH_2_, *J* = 1.50 Hz), 4.742 (d, 2H, -CH_2_-, *J* = 5.00 Hz), 3.996 (s, 2H, -CH_2_-); ^13^C-NMR (DMSO-*d*_6_, 125 MHz) δ: 168.521 (C=O), 160.267 (C=O), 156.314 (C=N), 146.697 (Q), 134.672 (Q), 131.362 (Q), 126.427 (Q), 125.923 (Q), 125.846 (Q), 118.644 (=CH_2_), 117.489 (Q), 47.788 (-CH_2_-), 35.716 (-CH_2_-).

#### 3.1.3. Synthesis of Compounds **1b**–**9b**

A total of 3 mmol (804 mg) of 3-benzyl-2-mercaptoquinazolin-4(3H)-one **B** and 3.5 (483 mg) mmol of potassium carbonate was added in 5 mL of dimethylformamide (DMF), in a glass flask. The mixture was stirred for 2 h at room temperature, and 3 mmol of α-bromoacetophenone derivatives 1, 2, 3, 4, 5, 7, 8, β-(α-bromoacetyl)-naphthalene **6,** or iodoacetamide **9**, were added. The mixture was stirred at room temperature for 4–6 h. The resulting precipitates were obtained by pouring on water and neutralized dropwise with a 5% hydrochloric acid solution. The final precipitates were filtered under a vacuum, dried and crystallized from dioxane. The compound **2b** was reported in the literature [32].

*3-benzyl-2-((2-(4-bromophenyl)-2-oxoethyl)thio)quinazolin-4(3H)-one* (**1b**): white solid; mp = 176 °C; yield = 63.67%; FT IR (KBr) ν_max_cm^−1^: 1683.55, 1672.95 (str C=O), 1548.56 (C=N), 696.18 (C-Br); MS: *m*/*z* = 465.2 [M+H^+^]; ^1^H-NMR (DMSO-*d*_6_, 500 MHz) δ: 8.079 (dd, 1H, Q, *J* = 8.00 and 1.50 Hz), 8.041 (d, 2H, Ar, *J* = 8.50 Hz), 7.822 (d, 2H, Ar, *J* = 8.50 Hz), 7.708 (td, 1H, Q, *J* = 7.75 and 1.50 Hz), 7.432 (td, 1H, Q, *J* = 7.25 and 1.00 Hz), 7.382–7.289 (m, 5H, Bz), 7.032 (d, 1H, Q, *J* = 8.50 Hz), 5.385 (s, 2H, -CH_2_-), 4.831 (s, 2H, -CH_2_-); ^13^C-NMR (DMSO-*d*_6_, 125 MHz) δ: 192.892 (C=O), 160.667 (C=O), 156.216 (C=N), 146.473 (Q), 135.505 (Ar), 135.407 (Bz), 134.854 (Q), 131.816 (Ar), 130.249 (Ar), 128.548 (Bz), 127.491 (Bz), 127.442 (Ar-Br), 126.805 (Bz), 126.581 (Q), 126.077 (Q), 125.384 (Q), 47.153 (-CH_2_-), 38.894 (-CH_2_-).

*3-benzyl-2-((2-oxo-2-phenylethyl)thio)quinazolin-4(3H)-one* (**2b**): white solid; mp = 164 °C; yield = 65.72%; FT IR (KBr) ν_max_cm^−1^: 1673.91, 1605.45 (str C=O), 1547.59 (C=N); MS: *m*/*z* = 387.3 [M+H^+^]; ^1^H-NMR (DMSO-*d*_6_, 500 MHz) δ: 8.111 (dd, 2H, Ar, *J* = 8.00 and 1.00 Hz), 8.077 (dd, 1H, Q, *J* = 8.00 and 1.00 Hz), 7.739-7.663 (m, 2H, Ar and Q), 7.602 (t, 2H, Ar, *J* = 7.75 Hz), 7.435-7.288 (m, 6H, 5Bz and 1Q), 7.012 (d, 1H, Q, *J* = 8.00 Hz), 5.390 (s, 2H, -CH_2_-), 4.859 (s, 2H, -CH_2_-); ^13^C-NMR (DMSO-*d*_6_, 125 MHz) δ: 193.473 (C=O), 160.681 (C=O), 156.279 (C=N), 146.501 (Q), 136.387 (Ar), 135.526 (Bz), 134.756 (Q), 133.398 (Ar), 128.716 (Ar), 128.545 (Bz), 128.226 (Ar), 127.428 (Bz), 126.826 (Bz), 126.553 (Q), 126.007 (Q), 125.377 (Q), 118.525 (Q), 47.139 (-CH_2_-), 39.006 (-CH_2_-).

*3-benzyl-2-((2-oxo-2-(p-tolyl)ethyl)thio)quinazolin-4(3H)-one* (**3b**): white solid; mp = 153 °C; yield = 60.21%; FT IR (KBr) ν_max_cm^−1^: 1682.59, 1668.61 (str C=O), 1542.29 (C=N); MS: *m*/*z* = 401.3 [M+H^+^]; ^1^H-NMR (DMSO-*d*_6_, 500 MHz) δ: 8.074 (dd, 1H, Q, *J* = 8.00 and 1.50 Hz), 8.003 (d, 2H, Ar, *J* = 4.25 Hz), 7.684 (td, 1H, Q, *J* = 8.00 and 1.50 Hz), 7.444–7.282 (m, 9H, 1Q, 2Ar and 5Bz), 7.064 (d, 1H, Q, *J* = 8.00 Hz), 5.385 (s, 2H, -CH_2_-), 4.831 (s, 2H, -CH_2_-), 2.422 (s, 3H, -CH_3_); ^13^C-NMR (DMSO-*d*_6_, 125 MHz) δ: 192.829 (C=O), 160.688 (C=O), 156.286 (C=N), 146.515 (Q), 143.841 (Ar-CH_3_), 135.540 (Bz), 134.749 (Q), 133.797 (Ar), 129.248 (Ar), 128.520 (Bz), 128.352 (Ar), 127.414 (Bz), 126.826 (Bz), 126.532 (Q), 125.972 (Q), 125.454 (Q), 118.525 (Q), 47.111 (-CH_2_-), 39.041 (-CH_2_-), 21.179 (-CH_3_).

*3-benzyl-2-((2-(4-chlorophenyl)-2-oxoethyl)thio)quinazolin-4(3H)-one* (**4b**): white solid; mp = 178 °C; yield = 70.84%; FT IR (KBr) ν_max_cm^−1^: 1684.03, 1672.46 (str C=O), 1547.11 (C=N), 775.24 (C-Cl); MS: *m*/*z* = 421.2 [M+H^+^]; ^1^H-NMR (DMSO-*d*_6_, 500 MHz) δ: 8.122 (d, 2H, Ar, *J* = 8.50 Hz), 8.076 (d, 1H, Q, *J* = 7.50 Hz), 7.714–7.665 (d, 3H, 1Q and 2Ar), 7.441-7.411 (m, 1H, Q), 7.380–7.311 (m, 5H, Bz), 7.023 (d, 1H, Q, *J* = 8.00 Hz), 5.384 (s, 2H, -CH_2_-), 4.835 (s, 2H, -CH_2_-); ^13^C-NMR (DMSO-*d*_6_, 125 MHz) δ: 192.661 (C=O), 160.660 (C=O), 156.209 (C=N), 146.466 (Q), 138.298 (Ar-Cl), 135.498 (Ar), 135.078 (Bz), 134.826 (Q), 130.151 (Ar), 128.849 (Ar), 128.534 (Bz), 127.435 (Bz), 126.805 (Bz), 126.567 (Q), 126.049 (Q), 125.363 (Q), 118.518 (Q), 47.146 (-CH_2_-), 38.894 (-CH_2_-).

*3-benzyl-2-((2-(3-nitrophenyl)-2-oxoethyl)thio)quinazolin-4(3H)-one* (**5b**): white solid; mp = 186 °C; yield = 62.21%; FT IR (KBr) ν_max_cm^−1^: 1698.96, 1673.43 (str C=O), 1542.29 (C=N), 1530.24 (C-NO_2_); MS: *m*/*z* = 432.2 [M+H^+^]; ^1^H-NMR (DMSO-*d*_6_, 500 MHz) δ: 8.793 (d, 1H, Ar, *J* = 2.00 Hz), 8.559–8.526 (m, 2H, Ar), 8.076 (dd, 1H, Q, *J* = 8.00 and 1.00 Hz), 7.915 (t, 1H, Ar, *J* = 8.00 Hz), 7.698-7.663 (m, 1H, Q), 7.443-7.411 (m, 1H, Q), 7.378–7.349 (m, 2H, Bz), 7.316-7.287 (m, 3H, Bz), 6.978 (d, 1H, Ar, *J* = 8.00 Hz), 5.385 (s, 2H, -CH_2_-), 4.911 (s, 2H, -CH_2_-); ^13^C-NMR (DMSO-*d*_6_, 125 MHz) δ: 192.682 (C=O), 160.632 (C=O), 156.146 (C=N), 147.978 (Q), 146.410 (Ar-NO_2_), 137.689 (Ar), 135.456 (Ar), 134.868 (Bz), 134.420 (Q), 130.613 (Ar), 128.548 (Bz), 127.554 (Ar), 127.449 (Bz), 126.770 (Bz), 126.602 (Q), 126.126 (Q), 125.286 (Q), 122.554 (Ar), 118.525 (Q), 47.188 (-CH_2_-), 38.817 (-CH_2_-).

*3-benzyl-2-((2-(naphthalen-2-yl)-2-oxoethyl)thio)quinazolin-4(3H)-one* (**6b**): white solid; mp = 152 °C; yield = 62.50%; FT IR (KBr) ν_max_cm^−1^: 1685.96, 1679.69 (str C=O), 1546.15 (C=N); MS: *m*/*z* = 437.2 [M+H^+^]; ^1^H-NMR (DMSO-*d*_6_, 500 MHz) δ: 8.898 (s, 1H, Ar), 8.177 (d, 1H, Ar, *J* = 8.00 Hz), 8.095-8.041 (m, 4H, 1Q and 3Ar), 7.730–7.598 (m, 3H, 1Q and 2Ar), 7.393-7.299 (m, 6H, 5Bz and 1Q), 6.995 (d, 1H, Q, *J* = 8.00 Hz), 5.406 (s, 2H, -CH_2_-), 5.006 (s, 2H, -CH_2_-); ^13^C-NMR (DMSO-*d*_6_, 125 MHz) δ: 193.326 (C=O), 160.681 (C=O), 156.321 (C=N), 146.508 (Q), 135.540 (Ar), 135.456 (Bz), 133.615 (Ar), 132.110 (Ar), 130.221 (Ar), 129.584 (Ar), 128.751 (Ar), 128.534 (Bz), 128.317 (Ar), 127.694 (Bz), 127.428 (Ar), 127.015 (Ar), 126.805 (Bz), 126.546 (Q), 125.993 (Q), 125.391 (Q), 123.788 (Ar), 118.532 (Q), 47.153 (-CH_2_-), 39.089 (-CH_2_-).

*3-benzyl-2-((2-(4-methoxyphenyl)-2-oxoethyl)thio)quinazolin-4(3H)-one* (**7b**): white solid; mp = 157 °C; yield = 63.30%; FT IR (KBr) ν_max_cm^−1^: 1672.95, 1598.70 (str C=O), 1549.04 (C=N), 1178.29 (C-O-C); MS: *m*/*z* = 417.3 [M+H^+^]; ^1^H-NMR (DMSO-*d*_6_, 500 MHz) δ: 8.111–8.087, (m, 3H, 1Q and 2Ar), 7.714–7.700 (m, 1H, Q), 7.441-7.435 (m, 1H, Q), 7.370–7.288 (m, 5H, Bz), 7.127–7.099 (m, 3H, 1Q and 2Ar), 5.394 (s, 2H, -CH_2_-), 4.825 (s, 2H, -CH_2_-), 3.891 (s, 3H, -CH_3_); ^13^C-NMR (DMSO-*d*_6_, 125 MHz) δ: 191.639 (C=O), 163.327 (Ar-O-CH_3_), 160.716 (C=O), 156.370 (C=N), 146.543 (Q), 135.568 (Bz), 134.805 (Q), 130.655 (Ar), 129.115 (Ar), 128.541 (Bz), 127.421 (Bz), 126.819 (Bz), 126.546 (Q), 126.007 (Q), 125.524 (Q), 118.539 (Q), 113.940 (Ar), 55.573 (-CH_3_), 47.097 (-CH_2_-), 38.852 (-CH_2_-).

*4-(2-((3-benzyl-4-oxo-3,4-dihydroquinazolin-2-yl)thio)acetyl)benzonitrile* (**8b**): white solid; mp = 175 °C; yield = 72.87%; FT IR (KBr) ν_max_cm^−1^: 2232.68 (C≡N), 1688.85, 1679.21 (str C=O), 1547.11 (C=N); MS: *m*/*z* = 412.2 [M+H^+^]; ^1^H-NMR (DMSO-*d*_6_, 500 MHz) δ: 8.246 (d, 2H, Ar, *J* = 8.50 Hz), 8.097-8.064 (m, 3H, 1Q and 2Ar), 7.692 (dt, 1H, Q, *J* = 7.75 and 1.75 Hz), 7.426 (t, 1H, Q, *J* = 7.50 and 1.00 Hz), 7.380–7.351 (m, 2H, Bz), 7.317-7.288 (m, 3H, Bz), 6.949 (d, 1H, Q, *J* = 8.00 Hz), 5.378 (s, 2H, -CH_2_-), 4.857 (s, 2H, -CH_2_-); ^13^C-NMR (DMSO-*d*_6_, 125 MHz) δ: 193.326 (C=O), 160.632 (C=O), 156.132 (C=N), 146.410 (Q), 139.726 (Ar), 135.463 (Bz), 134.868 (Q), 132.796 (Ar), 128.821 (Ar), 128.548 (Bz), 127.456 (Bz), 126.798 (Bz), 126.595 (Q), 126.105 (Q), 125.279 (Q), 118.511 (Ar), 118.119 (Q), 115.298 (Ar), 47.188 (-CH_2_-), 38.936 (-CH_2_-).

*2-((3-benzyl-4-oxo-3,4-dihydroquinazolin-2-yl)thio)acetamide* (**9b**): white solid; mp = 208 °C; yield = 76.10%; FT IR (KBr) ν_max_cm^−1^: 1672.46, 1639.68 (str C=O), 1549.52 (C=N); MS: *m*/*z* = 326.3 [M+H^+^]; ^1^H-NMR (DMSO-*d*_6_, 500 MHz) δ: 8.122 (dd, 1H, Q, *J* = 8.00 and 2.50 Hz), 7.839-7.805 (m, 1H, Q), 7.693 (s, 1H, -NH_2_), 7.565 (d, 1H, Q, *J* = 7.50 Hz), 7.480 (td, 1H, Q, *J* = 7.50 and 1.00 Hz), 7.361-7.332 (m, 2H, Bz), 7.301–7.267 (m, 3H, Bz), 7.240 (s, 1H, -NH_2_), 5.363 (s, 2H, -CH_2_-), 3.988 (s, 2H, -CH_2_-); ^13^C-NMR (DMSO-*d*_6_, 125 MHz) δ: 168.479 (C=O), 160.835 (C=O), 156.559 (C=N), 146.739 (Q), 135.588 (Bz), 134.812 (Q), 128.541 (Bz), 127.379 (Bz), 126.770 (Bz), 126.567 (Q), 126.028 (Q), 125.909 (Q), 118.658 (Q), 46.887 (-CH_2_-), 35.905 (-CH_2_-).

### 3.2. In Vivo Anticonvulsant Activity Evaluation

#### 3.2.1. Animals and Ethics

Young (6–10 weeks) female white mice, *Mus musculus* (CD1), with a weight of 25–35 g, were used. All mice were obtained from the Center for Experimental Medicine and Practical Skills of the University of Medicine and Pharmacy “Iuliu Haţieganu” Cluj-Napoca. The drugs used in the experiments were purchased from local suppliers, being produced by Terapia S.A. (Cluj-Napoca, Romania) and Hermes Arzneimittel (Großhesselohe, Germany).

The mice were kept under optimal conditions: constant temperature (22 ± 2 °C), stable air humidity (45 ± 10%), and a day–night cycle of 12/12 h. Each lot (*n* = 6) was placed separately in a type-IV polycarbonate cage. The animals had access to standard feeding and water ad libitum [33,34,35].

At the end of the experiment the mice were euthanized by anesthesia with a mixture of xylazine:ketamine = 1:2. An induced death, free of suffering by cervical dislocation, was carried out. The principles of ethics in scientific research in working with laboratory animals were applied in order to avoid any suffering of the animals used [36].

The study has been authorized by notice no. AVZ98/18.02.2022 of the Ethics Commission of the University of Medicine and Pharmacy “Iuliu Hațieganu” Cluj-Napoca and the authorization no. 300/01.04.2022 of the Sanitary Veterinary and Food Safety Directorate Cluj.

#### 3.2.2. Pentylenetetrazole (PTZ)-Induced Seizures

In this screening, 18 compounds (**1a**–**9a** and **1b**–**9b**) were tested for their anticonvulsant effect by evaluating their ability to prolong the latency of the first tonic-clonic seizure episode (Tc) and reduce the number of seizure episodes. The mice were weighed and divided into 57 groups (each group contained *n* = 6 mice): 1 negative control group, 2 positive control groups, and 54 groups for testing quinazolin-4-one derivatives, **1a**–**9a** and **1b**–**9b**, in three doses for each compound.

The substances were administered intraperitoneally (i.p.) to all groups of mice, as dispersions in water and a few drops of propylene glycol. The used doses for the compounds **1a**–**9a** and **1b**–**9b** were: D_1_ = 50 mg/kg, D_2_ = 100 mg/kg, and D_3_ = 150 mg/kg [37].

The negative control group received only the vehicle, water, and propylene glycol. Two positive control groups receiving 2 mg/kg of diazepam and 15 mg/kg of phenobarbital were used as reference anticonvulsants, in doses according to literature [20,37], chosen due to their mechanism of action, which targets both the GABA_A_ receptor [38,39,40,41] and carbonic anhydrase II [42,43,44,45].

Seizures were induced with 70 mg/kg of pentylenetetrazole (PTZ), given intraperitoneally 30 min after the administration of compounds [20,37].

The behavior of the mice, placed individually in a cage, was observed for 30 min, after the administration of pentylenetetrazole (PTZ). The latency of the first tonic-clonic seizure and episodes of tonic-clonic seizures in each animal (one episode of tonic-clonic spasm during at least 5 s) were counted. Mice that did not convulse within 30 min of observation were considered as protected [46].

Statistical analysis was performed using SPSS 15.0 software (Windows version 15.0, SPSS Inc., Chicago, IL, USA). Treated mice were compared by two-way analysis of variance (ANOVA) with a negative control group, followed by post hoc Dunnett’s multiple comparison. Results were expressed as mean ± standard error of mean (SEM). Differences were considered significant when *p* < 0.05. The evaluated parameters, at all three doses included the latency of the first seizure episode (Tc) and the number of seizure episodes for the mice used in this study during the first 30 min after the PTZ administration. The protection of mice against PTZ-induced seizures (%) was added.

#### 3.2.3. The Flumazenil Antagonism Assay

To highlight a potential GABAergic mechanism, the flumazenil antagonism test was used. The effect of flumazenil, a GABA_A_ receptor benzodiazepine binding site antagonist, was then evaluated for the most active compounds from each series, at the highest dose of 150 mg/kg. From “**a**” series, the chosen compound was **7a,** and from “**b**” series it was **8b**. The purpose of this method was to cancel the protective effect of diazepam; in clinical practice, flumazenil is used as an antidote for the benzodiazepine’s acute intoxications.

In the first part of the experiment, 18 mice were weighed and divided into 3 groups (*n* = 6 mice). The solutions were administrated intraperitoneally. The optimal dose of flumazenil that inhibited the diazepam activity in at least 50% to 100% of mice in a group was identified. The groups were treated as follows:

- **Group A**: 5 mg/kg flumazenil, 5 min later + 2 mg/kg diazepam (previously used dose), 30 min later + 70 mg/kg pentylenetetrazole (previously used dose);
- **Group B**: 7 mg/kg flumazenil, 5 min later + 2 mg/kg diazepam, 30 min later + 70 mg/kg pentylenetetrazole;
- **Group C**: 10 mg/kg flumazenil, 5 min later + 2 mg/kg diazepam, 30 min later + 70 mg/kg pentylenetetrazole.

The groups were monitored for 30 min. Mice from each group were placed individually in a cage and observed for the development of seizure episodes. 

The optimal dose chosen that inhibited the activity of diazepam was 5 mg/kg flumazenil.

The effect of flumazenil antagonism was evaluated in 12 mice, *Mus musculus* (CD1), 25–35 g, divided into 2 groups (n = 6 mice). The studied groups were as follows:

- **Group 1** (**7a**): 5 mg/kg flumazenil, 5 min later + 150 mg/kg compound **7a**, 30 min later + 70 mg/kg pentylenetetrazole;
- **Group 2** (**8b**): 5 mg/kg flumazenil, 5 min later + 150 mg/kg compound **8b**, 30 min later + 70 mg/kg pentylenetetrazole.

For 30 min, the mice were placed individually in a cage and observed for the development of seizures (episode of tonic-clonic spasm of at least 5 s). The number of unprotected mice in the PTZ-induced seizure model was recorded. Mice that did not convulse within 30 min of observation qualified as protected [46,47,48].

The obtained results were expressed as mean ± standard error of mean (SEM). The evaluated parameters included the protection against PTZ (%), the latency of the first seizure episode (Tc), and the number of seizure episodes for the mice used in this study during the first 30 min after PTZ administration. 

### 3.3. In Silico Studies

#### 3.3.1. LogP Prediction

The lipophilicity of substituent species plays a vital role in the significant differences observed in anticonvulsant activity. In general, an increased lipophilicity leads to a promising anticonvulsant effect [49].

In order to estimate the penetration of CNS barriers for the tested compounds, logP prediction was determined as a measure of lipophilicity, using the model implemented by Antonie et al. [50].

#### 3.3.2. Molecular Docking

The objective pursued by the in silico molecular docking study was to bring information and clarifications regarding the mechanism by which these compounds could act as anticonvulsant agents. For previously reported quinazoline-related compounds, two mechanisms associated with anticonvulsant activity were earlier identified in the literature: positive allosteric modulators of the GABA_A_ receptor [38,39,40,41] or acting as carbonic anhydrase II inhibitors [43,51]. Considering these two possible biological targets, the present in silico study intended to evaluate the quinazolin-4-one derivatives compounds with a dual mechanism of action, as positive allosteric modulators of the GABA_A_ receptor and potential inhibitors of carbonic anhydrase II.

The files containing the 3D structure of the compounds and targeted macromolecules in the molecular docking were prepared using the standard previously reported procedure using AutoDock Tools 1.5.6 and OpenBabel 2.3.2 [52,53,54,55].

The main molecular docking study employed the using of AutoDock, with a specialized force field due to the zinc ion [55,56,57]. The search space was set as a cube, with sides x = y = z = 55, and the grid space was set to 0.375. For all ligands, 200 conformations were generated and grouped in clusters with relative root mean square deviation of 2 Å.

To confirm the results of the main docking study, but primarily to avoid the false-positive results frequently encountered in molecular docking studies, a secondary molecular docking study was made using AutoDock vina 1.2.1, according to our previously reported settings, but the search space was set as a cube with sides x = y = z = 20 [58,59]. To remove the false positive results, the use of more than one docking software and clustering of the predicted conformations are common practices in the field of in silico research of bioactive compounds. For the same reason, to improve the molecular docking studies, another additional method used in the modern drug design research is represented by the molecular dynamics studies [60,61,62,63,64,65].

The 3D structures of the macromolecules targeted in the molecular docking study were taken from the Protein Data Bank, with codes 6X3Z (GABA_A_ receptor, subtype α_1_β_2_γ_2_) and 3F8E (carbonic anhydrase II) [66,67,68]. The center of the search space was set to x = 81.211, y = 120.374, z = 119.414 for the GABA_A_ receptor and x = -5.095, y = 6.414, z = 9.427 for the carbonic anhydrase II.

The visualization of the molecular interactions between the ligands and the macromolecules was performed using Chimera 1.10.2 [69]. For the resulted complexes 2D interaction diagrams were generated using LigPlot^+^ 2.2 [70,71].

#### 3.3.3. Molecular Dynamics Simulation

Molecular dynamics simulation techniques recently became an important tool in developing new bioactive agents, as a complementary tool to the molecular docking techniques [72,73,74,75].

The literature described the effect of methaqualone and its quinazolin-4-one analogues as positive allosteric modulators of the GABA_A_ receptor [7,74,76]. Consequently, this study focused on carbonic anhydrase II as the second potential target for our compounds.

The stability of the predicted compound–carbonic anhydrase II complexes was evaluated in an in silico molecular dynamics study. Compounds that formed complexes with carbonic anhydrase II, as evaluated in the molecular dynamics study, were those with higher affinity in the first quartile, according to the enzyme rankings from the molecular docking study: **3b**, **4b**, **5b**, **7b,** and **8b**. Supplementary to the current list were added compounds **5a** and **7a**, being the ones with high affinity to the benzodiazepine binding site from the GABA_A_ receptor. This was made with the aim of identifying if there would be two possible simultaneous mechanisms for these compounds.

The molecular dynamics simulations involving the compounds–carbonic anhydrase II complexes were performed with GROMACS 2023 [77] using CHARMM36 force field [78] according to previous reports in literature [79,80,81]. For each of the aforementioned compounds, the top binding conformation from the molecular docking study was taken in order to construct the systems for simulation. Further, the obtained systems were solvated using the TIP3P water model [82] (approximately 9600 molecules) in a dodecahedron box, with a minimum distance of 1 nm to each side [79,80,82]. The parametrization of the ligands was made using CgenFF server [78]. The systems were neutralized with sodium and chloride ions (150 mM final concentration), resulting in systems comprised of approximately 33,000 atoms each.

The constructed systems were energy-minimized until convergence using the steepest descent method to remove the close contacts (50,000 steps and maximum force < 10.0 kJ/mol) [60,83,84].

The equilibration of the systems was carried out in the canonical ensemble (NVT for 500 ps at 300 K using Berendsen thermostat), while for production, the isothermal–isobaric ensemble was assessed (NPT for 500 ps at 1 bar using Parrinello–Rahman barostat) [85]. Particle Mesh Ewald (PME) method was used for the long-range electrostatic forces [85,86].

The molecular dynamics simulations were run for 100 ns performed on a Debian 11 machine equipped with a NVIDIA RTX 3060 GPU. Visualization of the evolution in time of the compounds–carbonic anhydrase II complexes during the molecular dynamics simulation was made using VMD 1.9.4 [87].

## 4. Conclusions

A total of 18 chemically synthesized compounds, **1a**–**9a** and **1b**–**9b**, grouped in two series, “**a**” and “**b**”, were obtained by the condensation of 3-allyl-2-mercaptoquinazolin-4(3H)-one or 3-benzyl-2-mercaptoquinazolin-4(3H)-one with some α-bromoacetophenone derivatives, β-(α-bromoacetyl)-naphthalene, and iodoacetamide.

The anticonvulsant activity of the “**a**” and “**b**” series, as potentially positive allosteric modulators of the GABA_A_ receptor, was evaluated in vivo through the PTZ-induced seizure model in mice. The anticonvulsant potential was influenced by the nature of substituents in positions 2 and 3 of the quinazolin-4-one nucleus, which was enhanced by an increased lipophilicity. The best anticonvulsant activity was demonstrated by the compounds substituted in position 3 with the benzyl moiety (“**b**” series) and with an electron-withdrawing substituent (EWG) in position 2. Among these compounds, compound **8b** (R^1^ = p-CN-C_6_H_4_-) could be considered the lead compound of series, based on its promising results at the highest dose of 150 mg/kg. Furthermore, some compounds, **9a** and **9b**, substituted in position 2 with an unsubstituted amide and with low lipophilicity values, have showed lack of anticonvulsant action.

After blocking the benzodiazepine binding site on GABA_A_ receptors with flumazenil, the substances with the highest anticonvulsant effect (**7a**, **8b**) did not exhibit a protective capacity against PTZ-induced seizures, suggesting a potential anticonvulsant mechanism of action as positive allosteric modulators of GABA_A_ receptor at the benzodiazepine binding site.

The in silico studies showed that compounds from the “**b**” series had a higher binding affinity for the GABA_A_ receptor than compounds from the “**a**” series, while the ligand-carbonic anhydrase II predicted complexes were instable for both series.

Thus, the tested compounds suggested interactions at the GABA_A_ receptor level, most likely as positive allosteric modulators, but affinity tests for GABA_A_ receptors are needed to confirm the mechanism of action more accurately. The instability of complexes on carbonic anhydrase II and reduced binding energies argues for the absence of this mechanism of action.

The compounds from the “**a**” and “**b**” series do not exhibit a dual mechanism at both types of targets.

## Figures and Tables

**Table 1:** The results of the molecular docking study targeting the GABA_A_ receptor and carbonic anhydrase II.

Comp.	GABA_A_ Receptor				Carbonic Anhydrase II			
	AutoDock			AutoDock Vina	AutoDock			AutoDock Vina
	Global ΔG_min_ (kcal/mol)	The Most Populated Cluster		Global ΔG_min_ (kcal/mol)	Global ΔG_min_ (kcal/mol)	The Most Populated Cluster		Global ΔG_min_ (kcal/mol)
		Cluster ΔG_min_ (kcal/mol)	No. Poses			Cluster ΔG_min_ (kcal/mol)	No. Poses	
**“a” series**								
**1a**	−10.07	−9.59	96	−8.65	−7.50	−6.19	30	−6.49
**2a**	−9.67	−9.67	47	−9.17	−6.69	−6.42	70	−6.27
**3a**	−9.91	−9.31	70	−9.40	−7.22	−6.50	31	−6.50
**4a**	−9.95	−9.35	87	−9.64	−7.07	−6.51	35	−6.62
**5a**	−10.88	−10.58	68	−10.16	−7.99	−7.75	20	−7.02
**6a**	−10.28	−10.10	35	−9.99	−7.28	−6.78	67	−6.04
**7a**	−11.43	−11.43	96	−11.19	−7.90	−5.85	43	−6.25
**8a**	−9.97	−9.97	22	−9.72	−7.48	−6.84	55	−6.30
**9a**	−8.18	−7.48	63	−8.09	−6.07	−5.56	115	−6.09
**“b” series**								
**1b**	−10.29	−9.55	39	-9.40	−7.86	−7.49	28	−7.20
**2b**	−10.40	−10.17	77	−10.11	−7.53	−7.27	54	−7.15
**3b**	−10.66	−10.54	90	−10.10	−8.01	−6.74	25	−7.33
**4b**	−10.70	−10.50	69	−9.98	−8.09	−6.65	24	−7.45
**5b**	−11.41	−11.08	59	−10.22	−8.62	−7.81	22	−8.10
**6b**	−9.99	−9.85	42	−9.55	−7.69	−7.31	30	−7.15
**7b**	−10.21	−9.12	53	−10.01	−8.24	−6.50	34	−7.25
**8b**	−11.27	−10.84	51	−10.55	−8.70	−7.39	43	−7.26
**9b**	−9.12	−8.66	79	−7.93	−6.07	−5.56	115	−6.09

Compounds with affinity higher than the 1st quartile from each series were highlighted. **Comp.** = compounds (“**a**” series and “**b**” series). **Global ΔG_min_** = global minimum binding free energy, measured in kcal/mol. **Cluster ΔG_min_** = minimum binding free energy within a cluster of conformations or poses generated during the docking process.

## Data Availability

Data are contained within the article and Supplementary Materials.

## Supplementary Materials

The following supporting information can be downloaded at: https://www.mdpi.com/article/10.3390/molecules29091951/s1, Figures S1–S9: The IR spectrum for the compounds **1a**–**9a**; Figures S10–S18: The IR spectrum for the compounds **1b**–**9b**; Figures S11–S25: The MS spectrum for the compounds **1a**–**9a**; Figures S26–S34: The MS spectrum for the compounds **1b**–**9b**; Figures S35–S43: The ^1^H-NMR spectrum for the compounds **1a**–**9a**; Figures S44–S52: The ^1^H-NMR spectrum for the compounds **1b**–**9b**; Figures S53–S61: The ^13^C-NMR spectrum for the compounds **1a**–**9a**; Figures S62–S70: The ^13^C-NMR spectrum for the compounds **1b**–**9b**. Figures S71–S79: The best binding pose of compounds **1a**–**9a** and **1b**–**9b** in the benzodiazepine binding site of GABA_A_ receptor. Figures S80: RMSD (A) of the ligands during the 100 ns simulation in complex with carbonic anhydrase II. Table S1. Protection against PTZ-induced seizure model, time until the first seizure, mean number of seizures, and LogP, for the compounds of the “**a**” series, in the first 30 min after PTZ administration. Table S2. Protection against PTZ-induced seizure model, time until the first seizure, mean number of seizures, and LogP, for the compounds of the “**b**” series, in the first 30 min after PTZ administration. Table S3. Results obtained in flumazenil antagonism assay for the compounds **7a** and **8b**.
