# Supplementary material for: Synthesis, In Vivo Anticonvulsant Activity Evaluation and In Silico Studies of Some Quinazolin-4(3H)-One Derivatives
Source: Molecules. 2024 Apr 24;29(9):1951. doi: 10.3390/molecules29091951 (PMC11085150; doi:10.3390/molecules29091951)
Supplement: Supplementary file 1 [file molecules-29-01951-s001.zip › molecules-2963454-supplementary.pdf]

Article

Supplementary Materials

1. Figures

1.1. The IR spectra

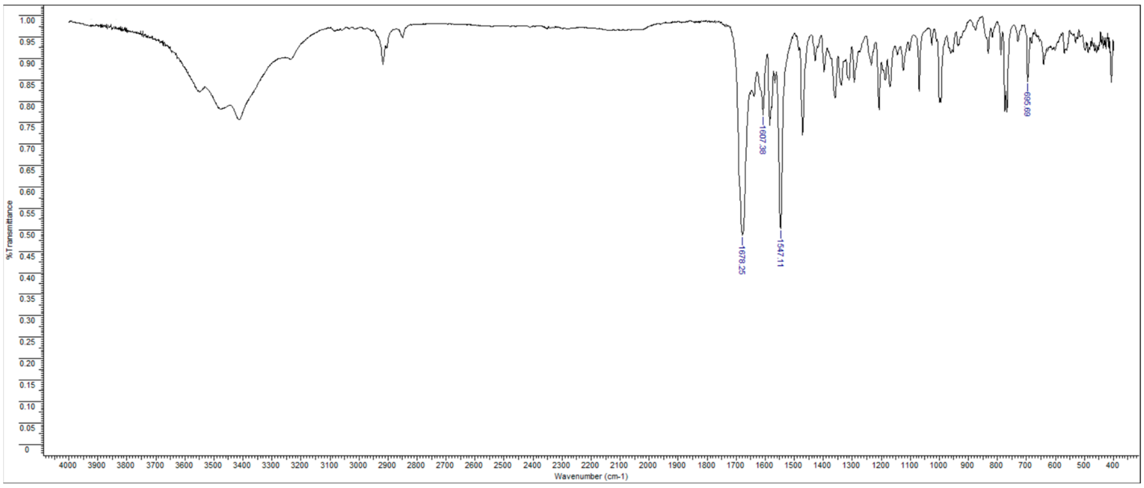

Figure S1. The IR spectrum for the compound **1a.**

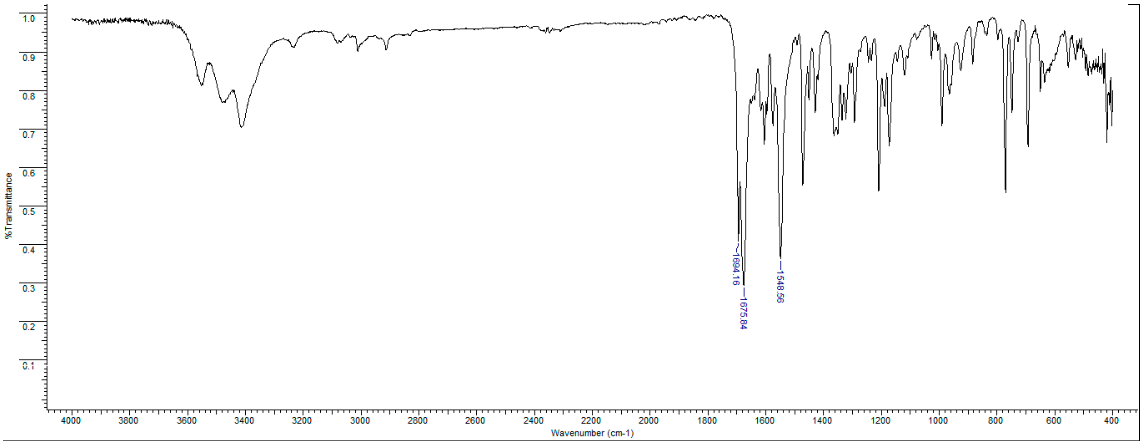

Figure S2. The IR spectrum for the compound **2a.**

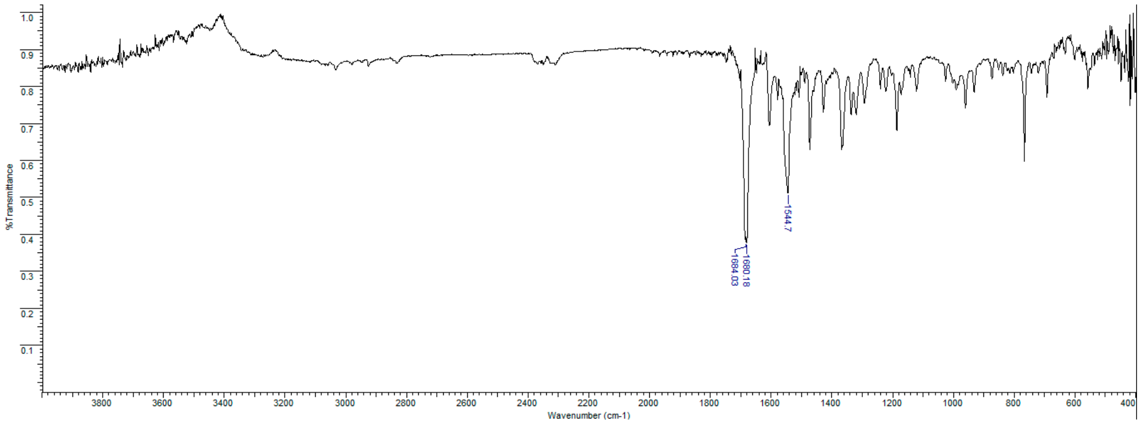

Figure S3. The IR spectrum for the compound **3a.**

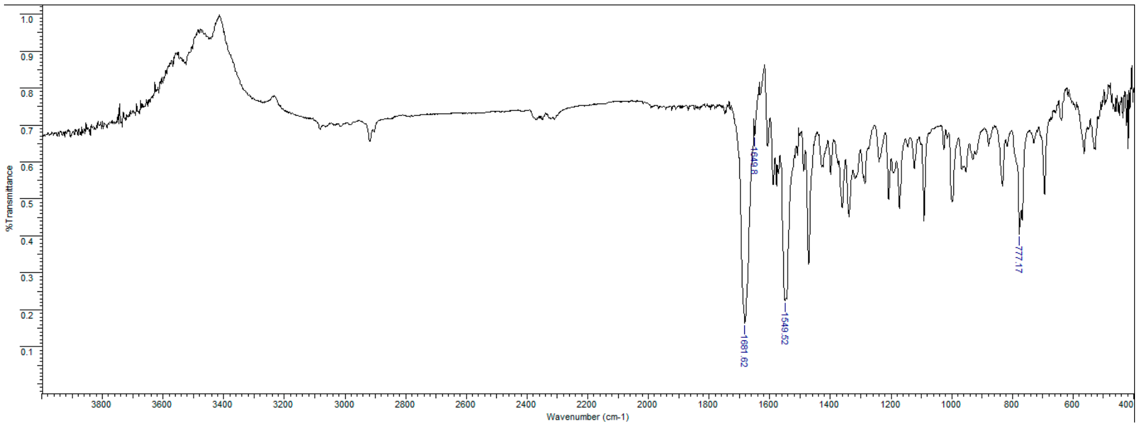

Figure S4. The IR spectrum for the compound **4a.**

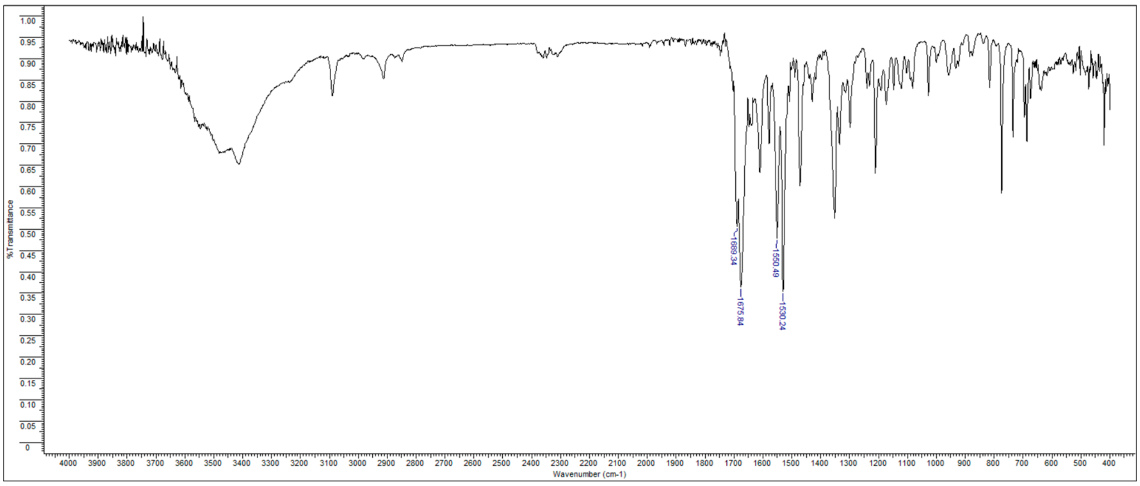

Figure S5. The IR spectrum for the compound **5a.**

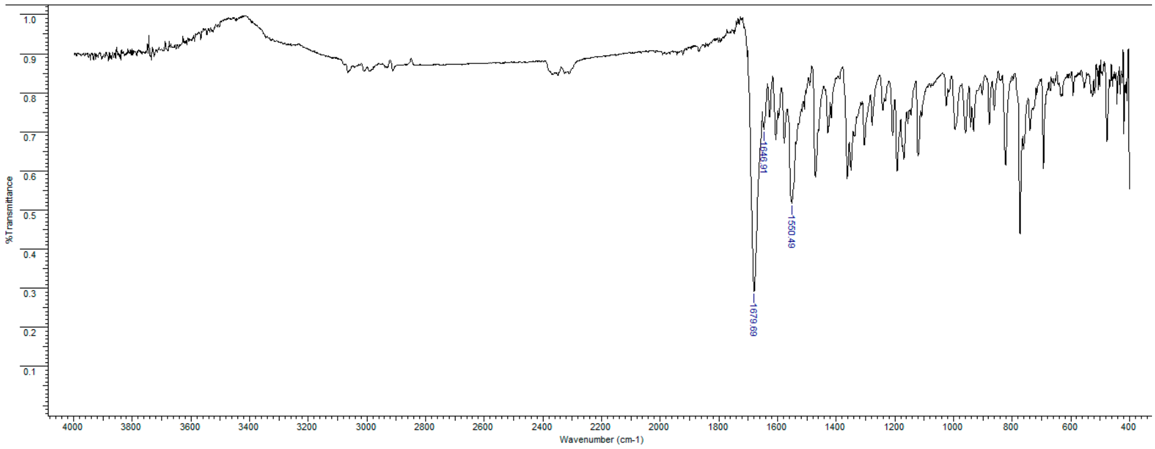

Figure S6. The IR spectrum for the compound **6a.**

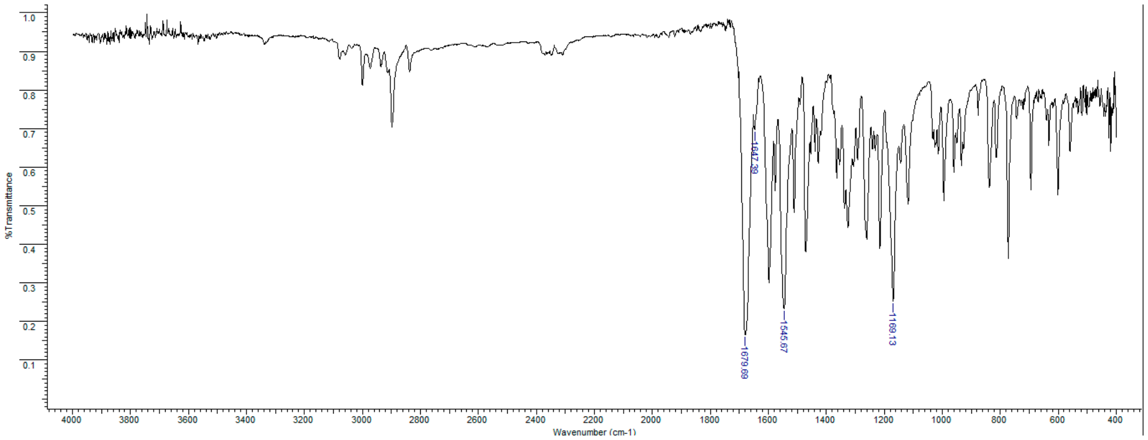

Figure S7. The IR spectrum for the compound **7a.**

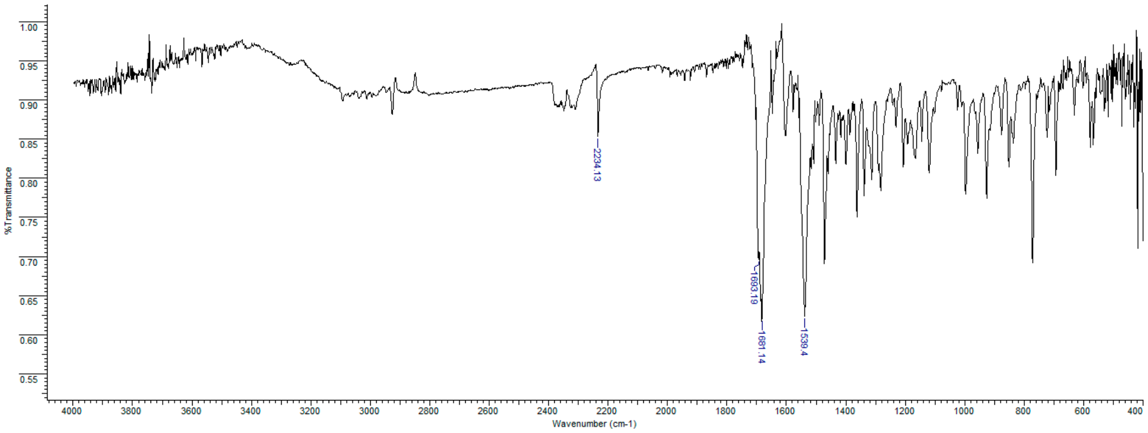

Figure S8. The IR spectrum for the compound **8a.**

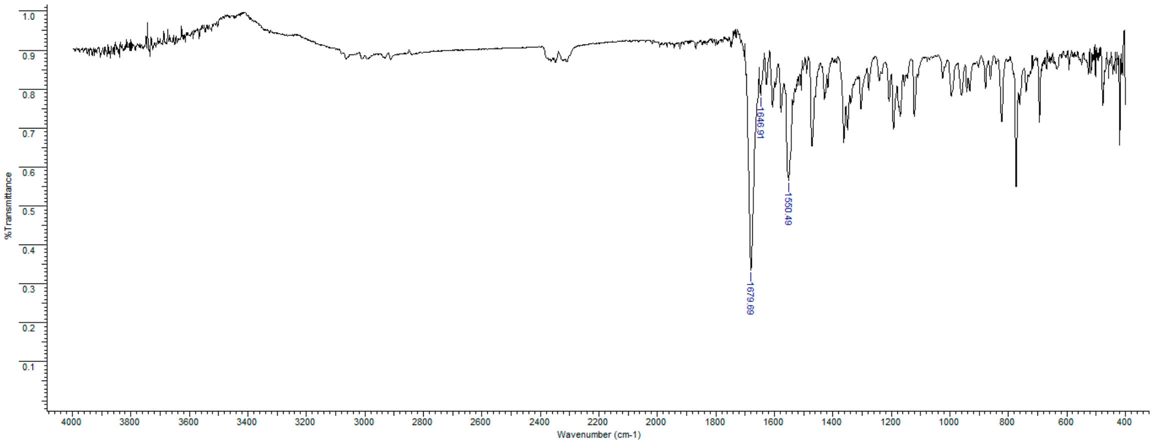

Figure S9. The IR spectrum for the compound **9a.**

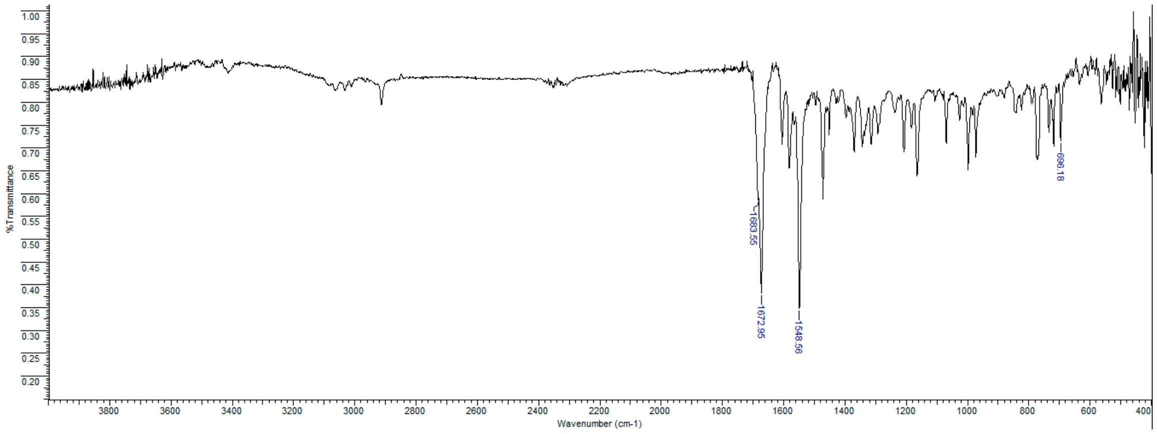

Figure S10. The IR spectrum for the compound **1b.**

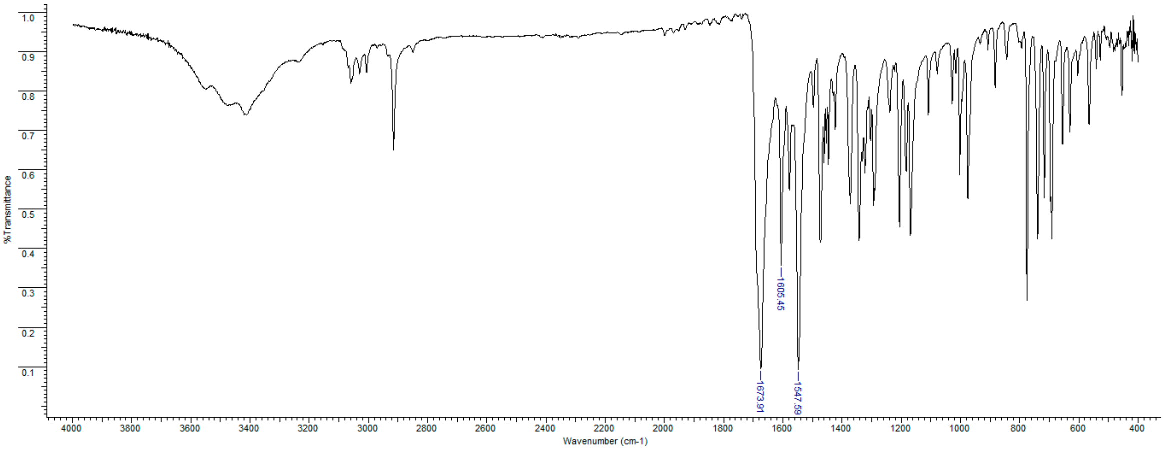

Figure S11. The IR spectrum for the compound **2b.**

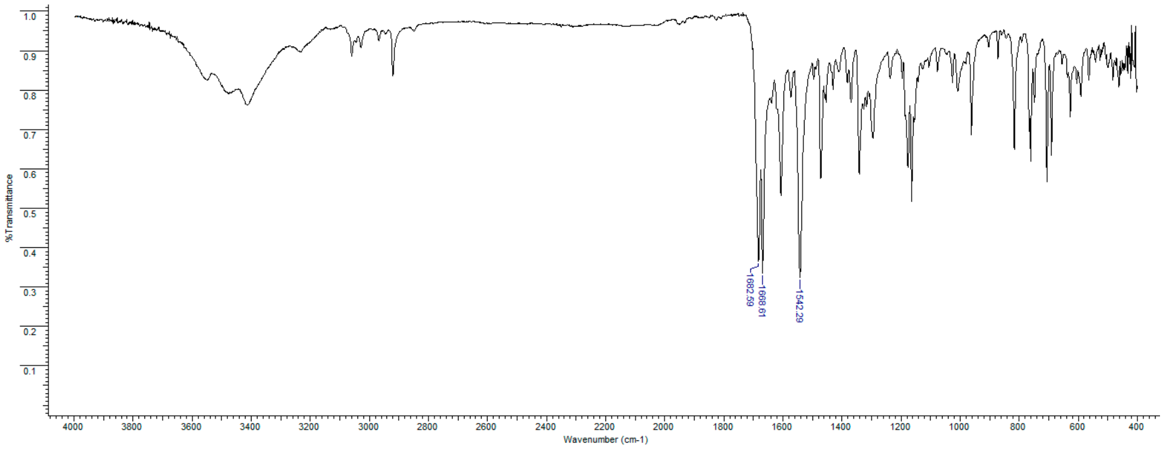

Figure S12. The IR spectrum for the compound **3b.**

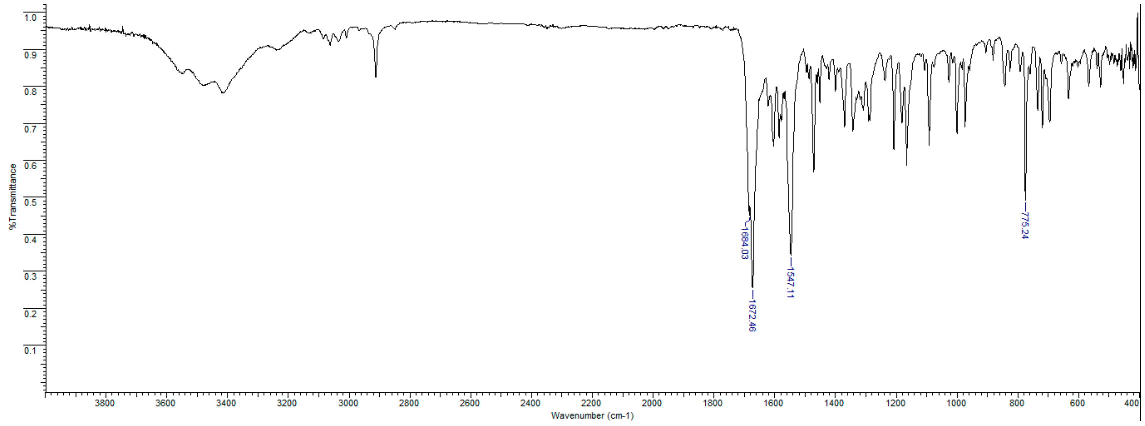

Figure S13. The IR spectrum for the compound **4b.**

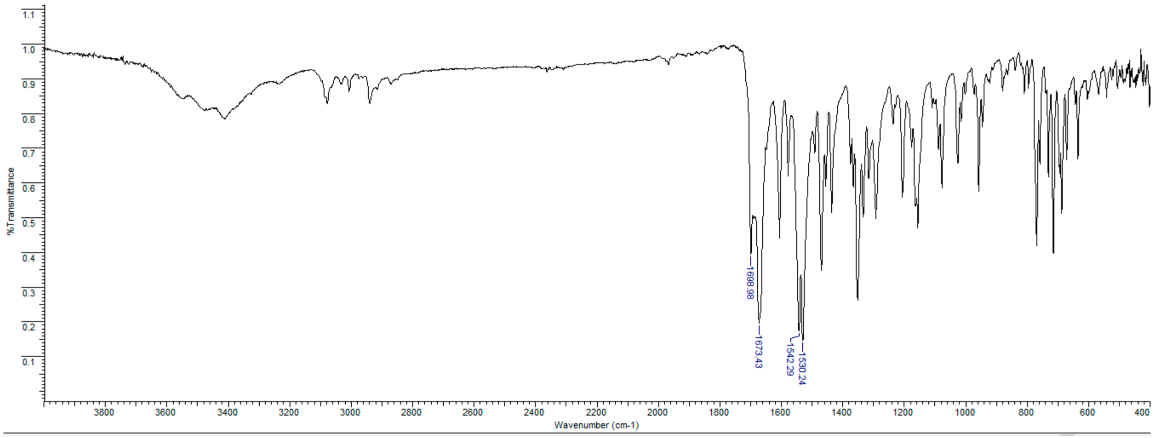

Figure S14. The IR spectrum for the compound **5b.**

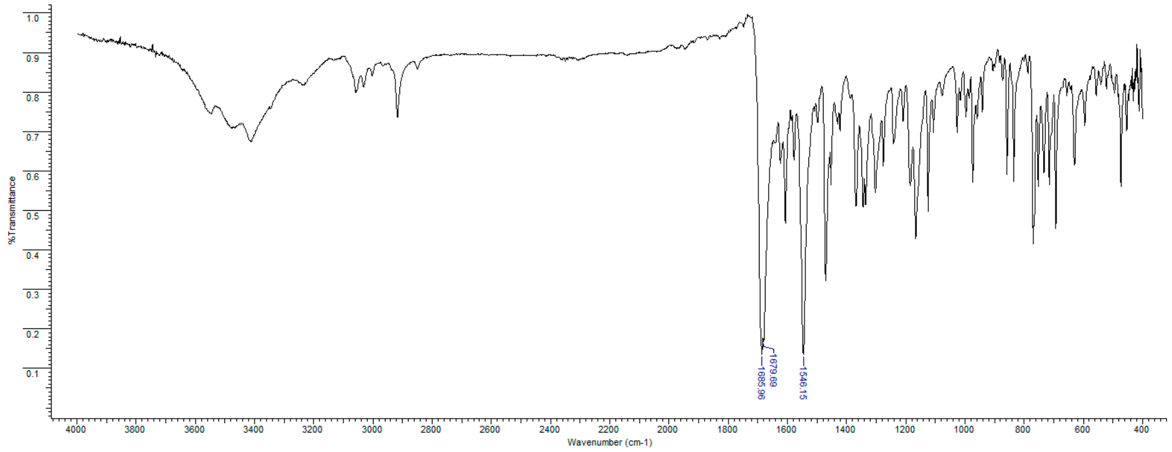

Figure S15. The IR spectrum for the compound **6b.**

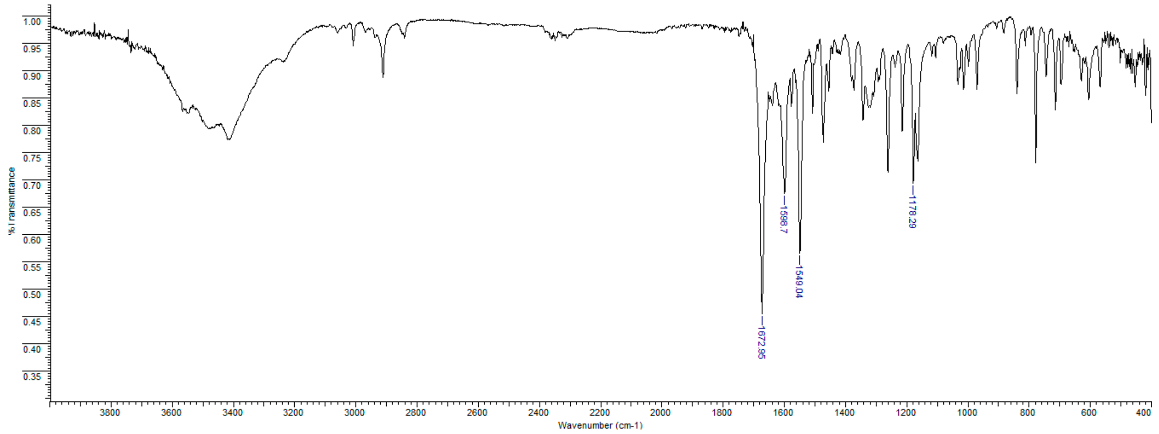

Figure S16. The IR spectrum for the compound **7b.**

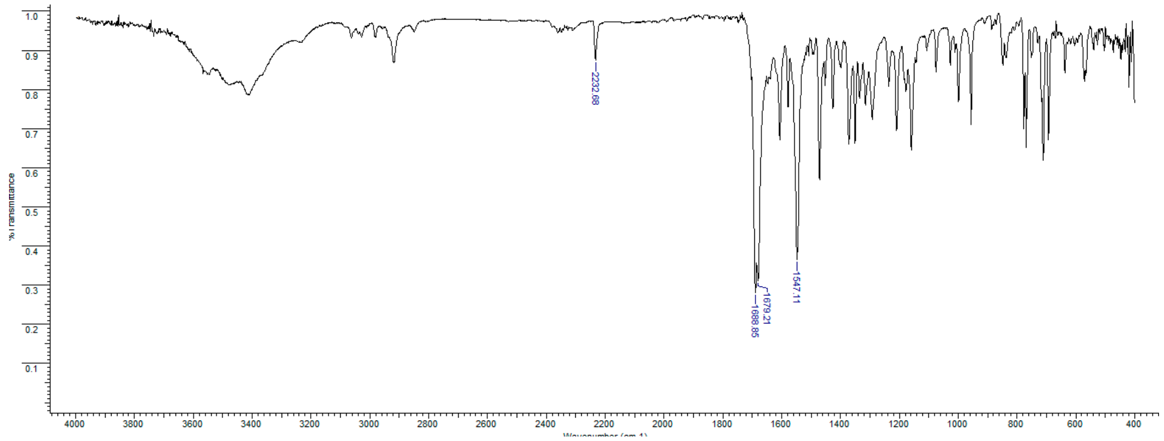

Figure S17. The IR spectrum for the compound **8b.**

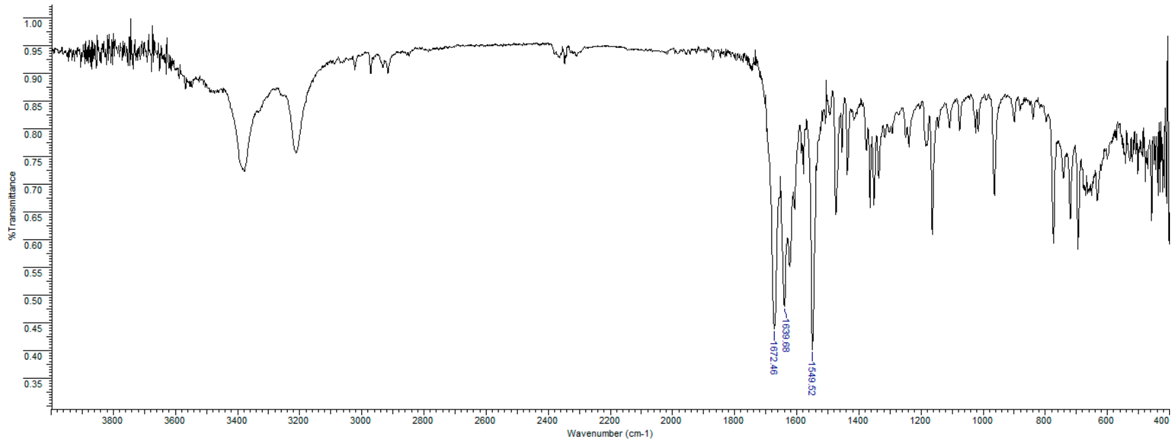

Figure S18. The IR spectrum for the compound **9b.**

1.1. The MS spectra

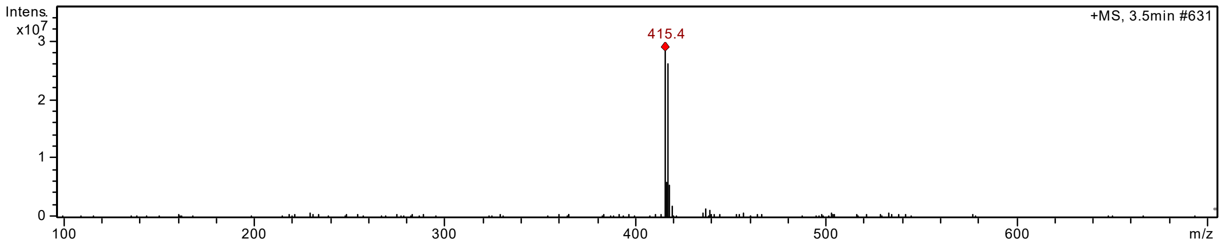

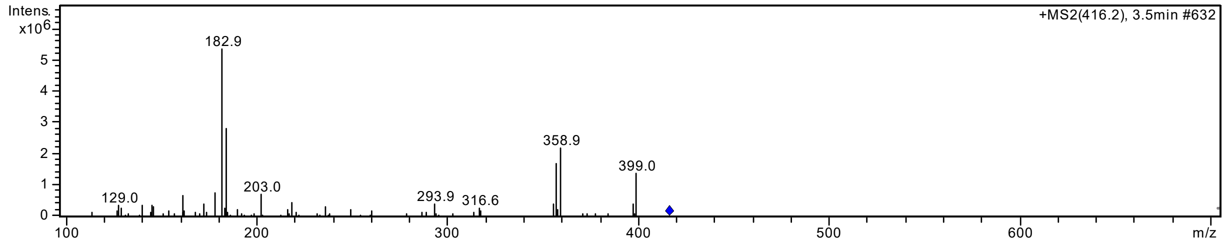

Figure S19. The MS spectrum for the compound **1a.**

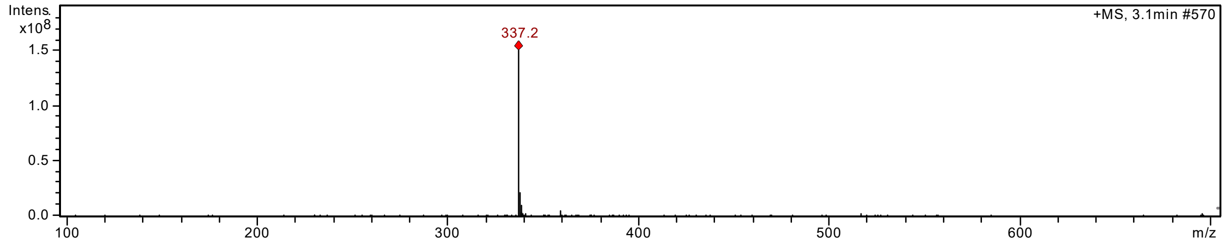

Figure S20. The MS spectrum for the compound **2a.**

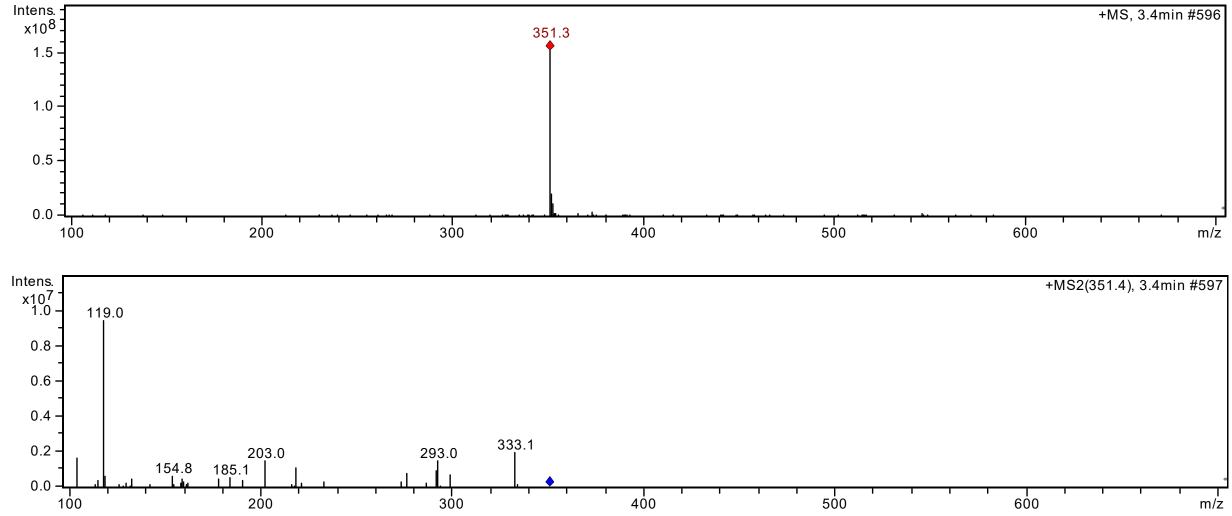

Figure S21. The MS spectrum for the compound **3a.**

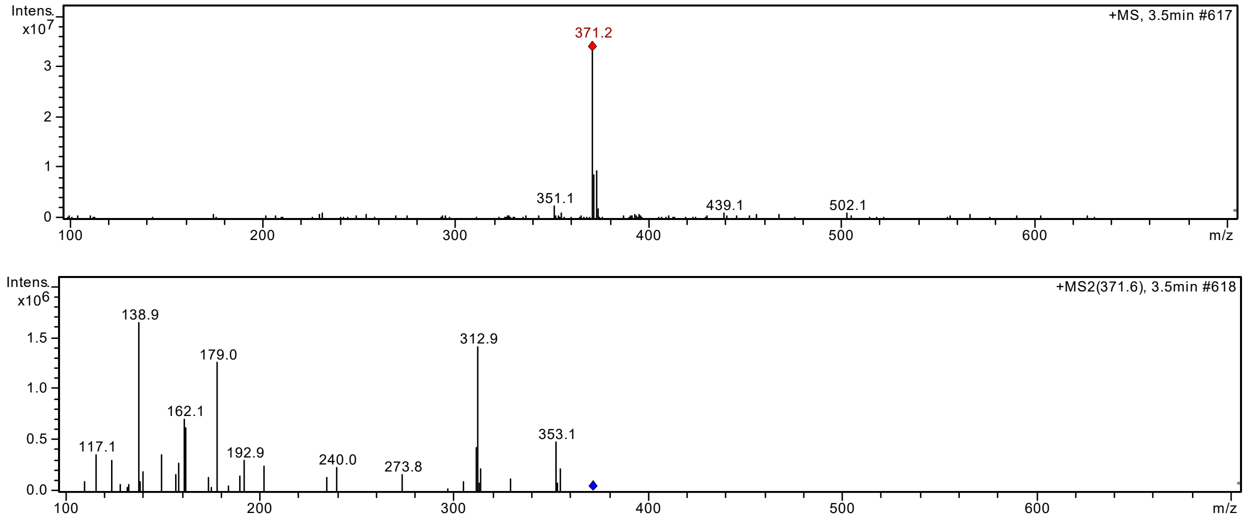

Figure S22. The MS spectrum for the compound **4a.**

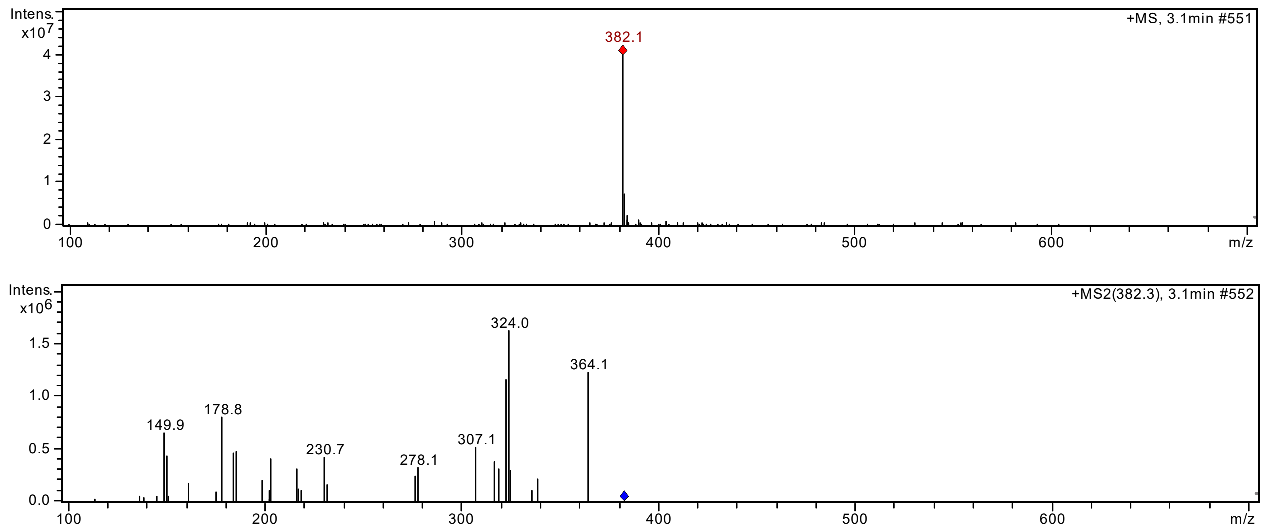

Figure S21. The MS spectrum for the compound **5a.**

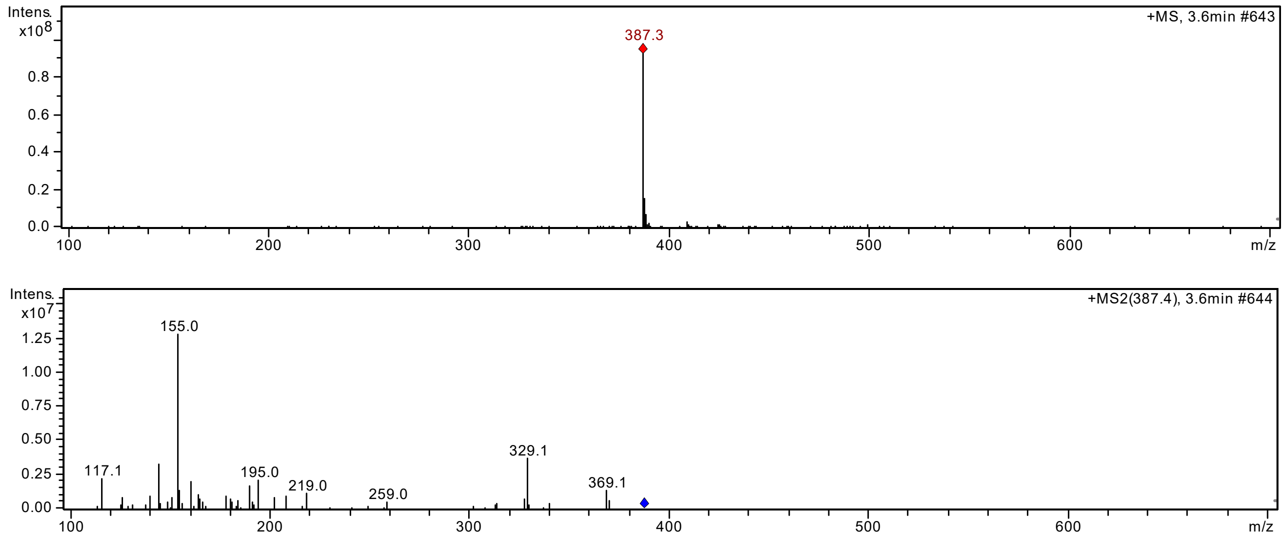

Figure S22. The MS spectrum for the compound **6a.**

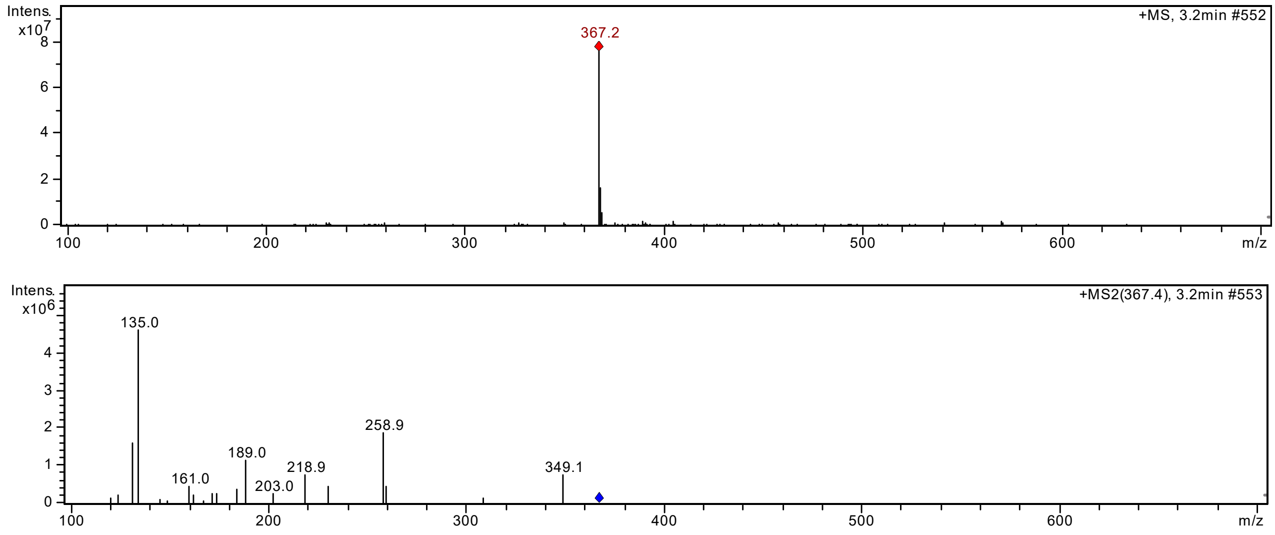

Figure S23. The MS spectrum for the compound **7a.**

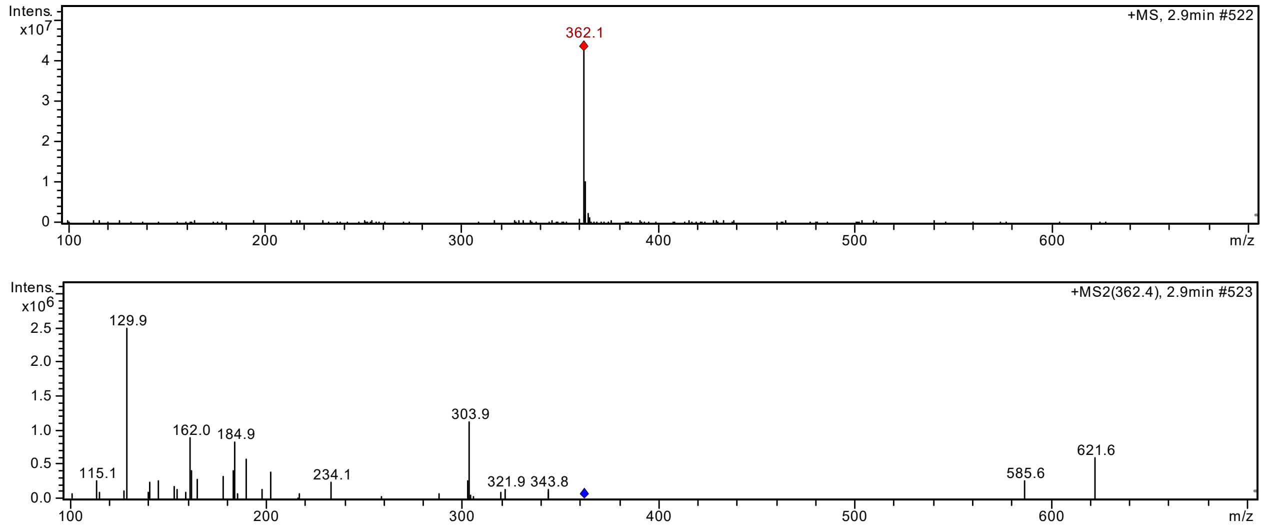

Figure S24. The MS spectrum for the compound **8a.**

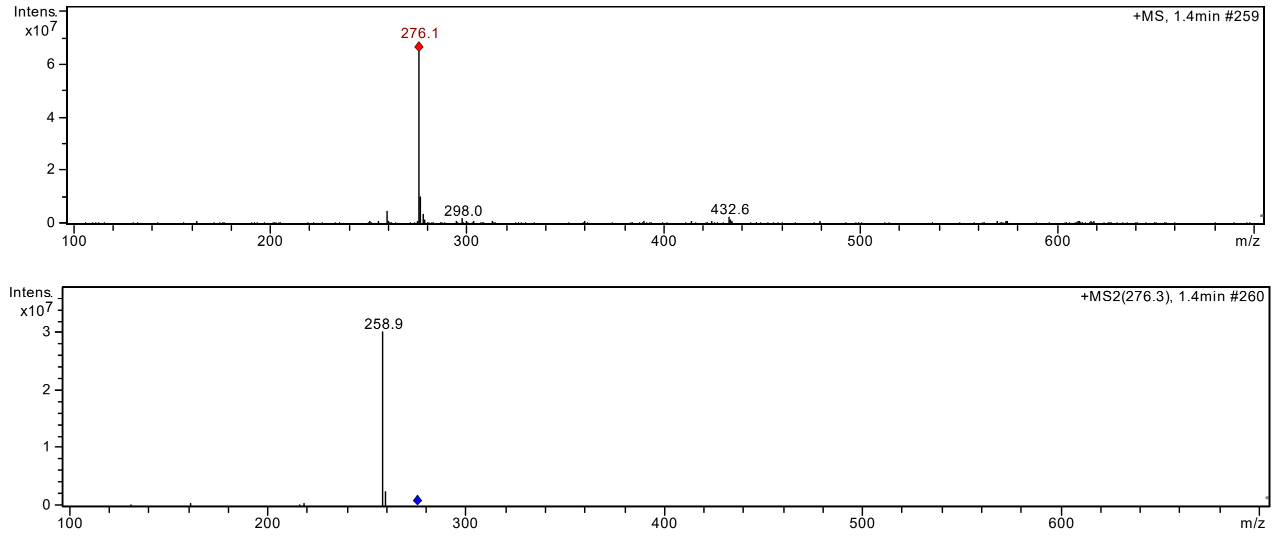

Figure S25. The MS spectrum for the compound **9a.**

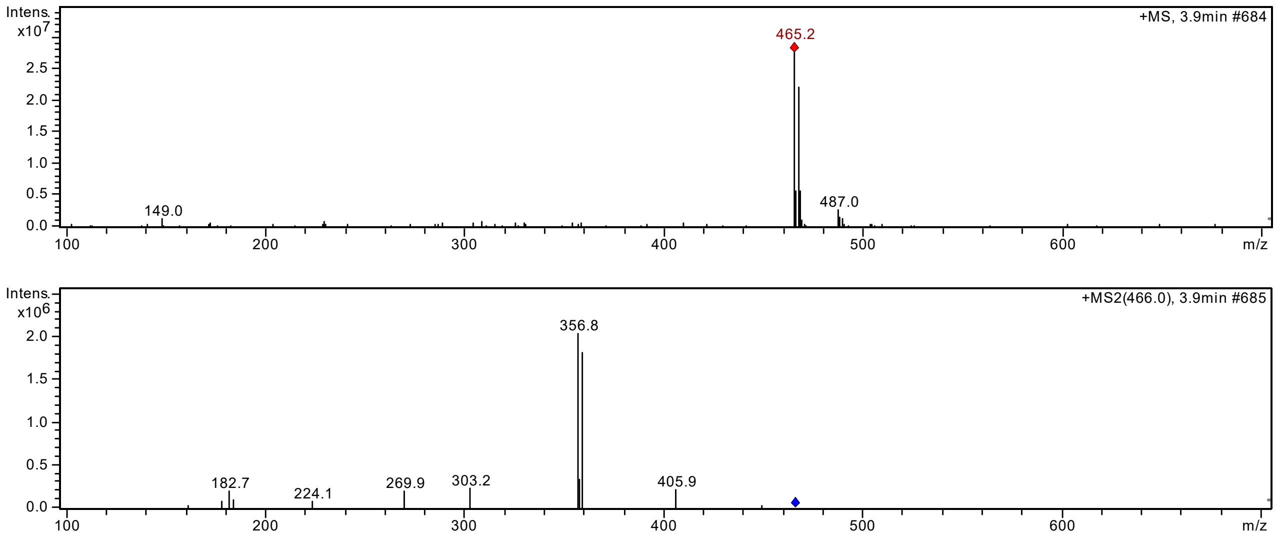

Figure S26. The MS spectrum for the compound **1b.**

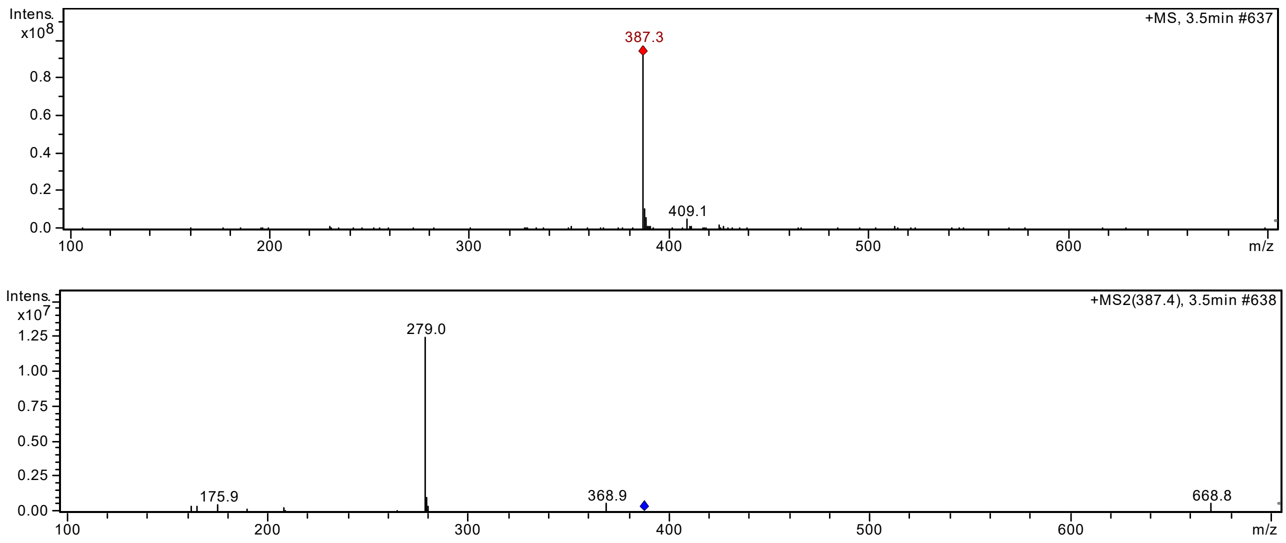

Figure S27. The MS spectrum for the compound **2b.**

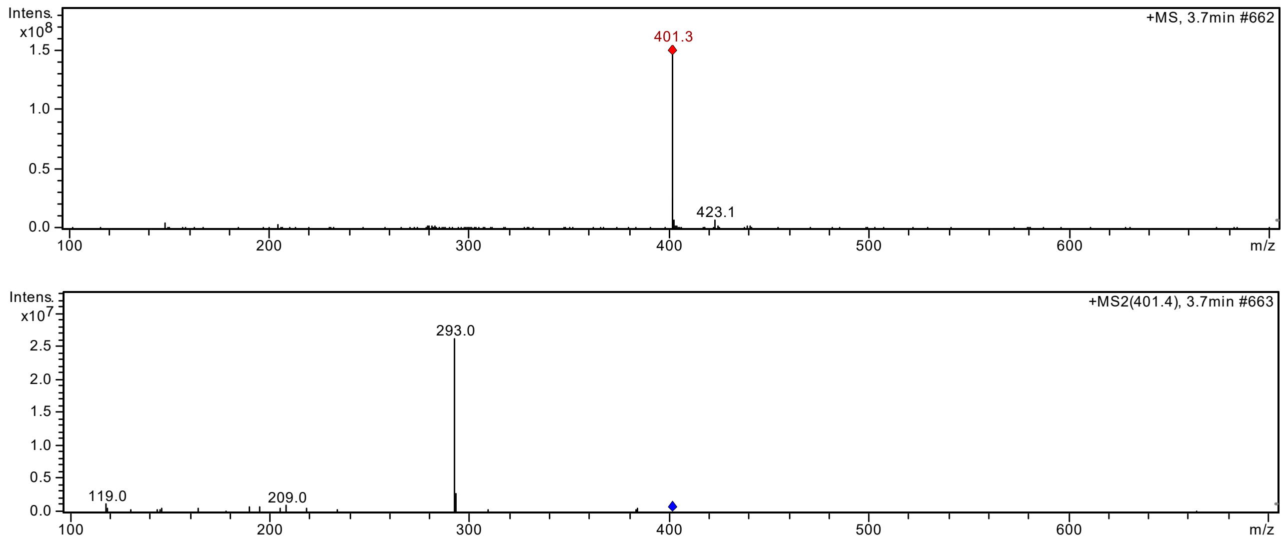

Figure S28. The MS spectrum for the compound **3b.**

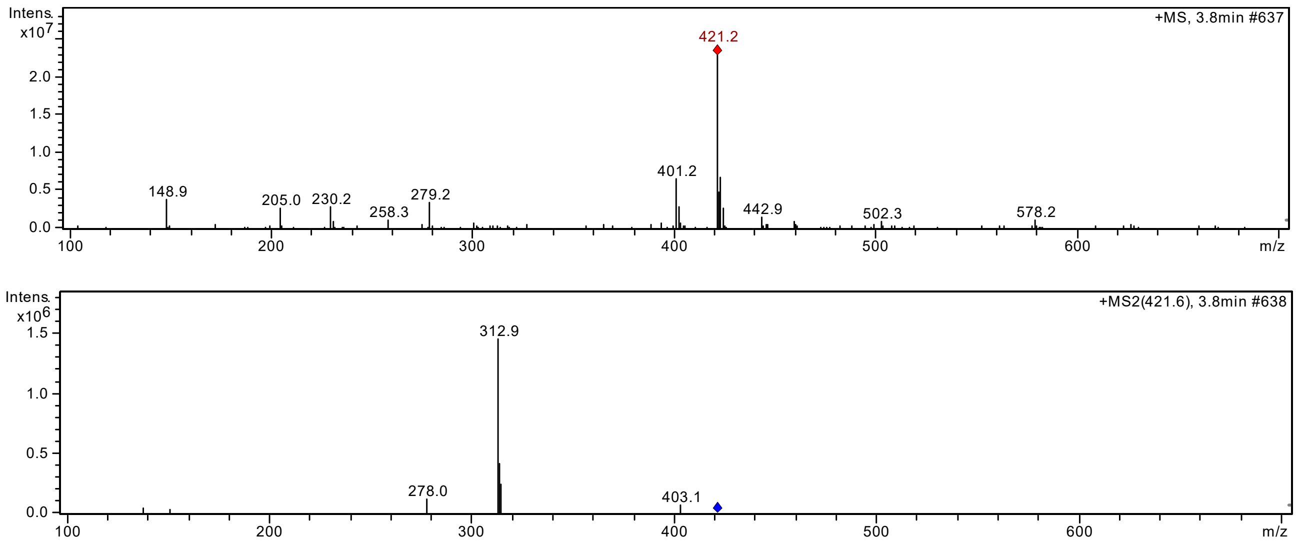

Figure S29. The MS spectrum for the compound **4b.**

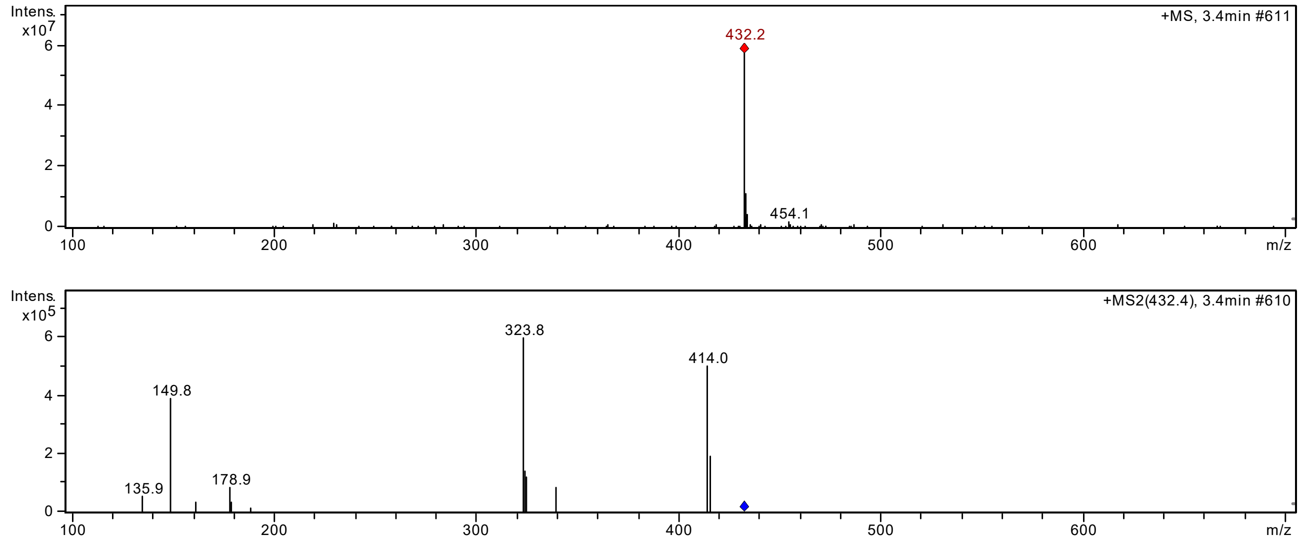

Figure S30. The MS spectrum for the compound **5b.**

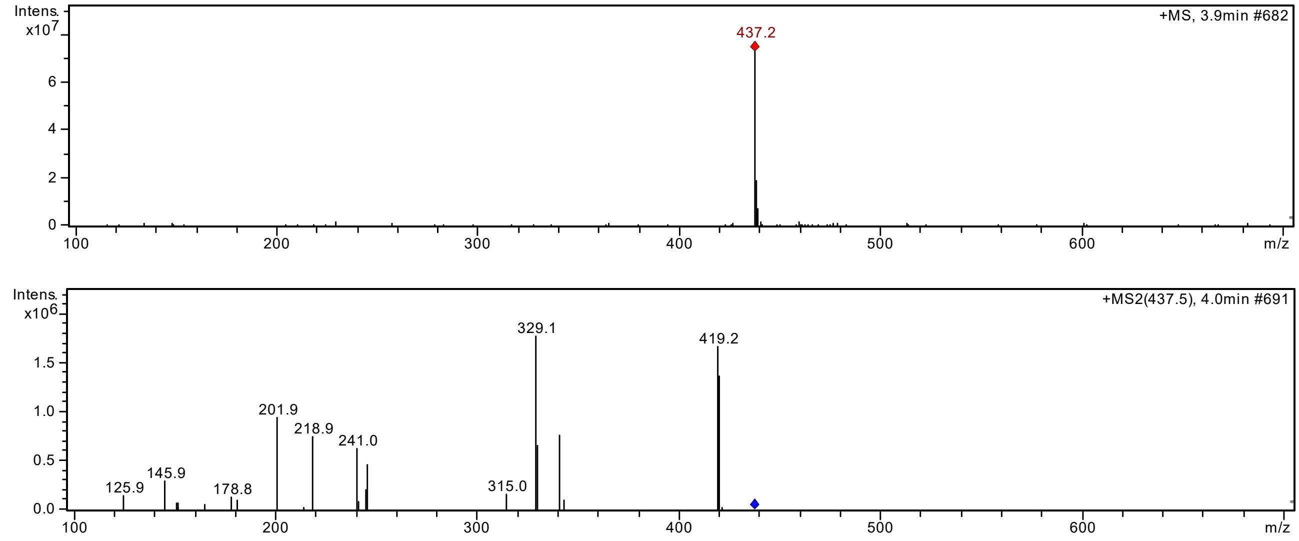

Figure S31. The MS spectrum for the compound **6b.**

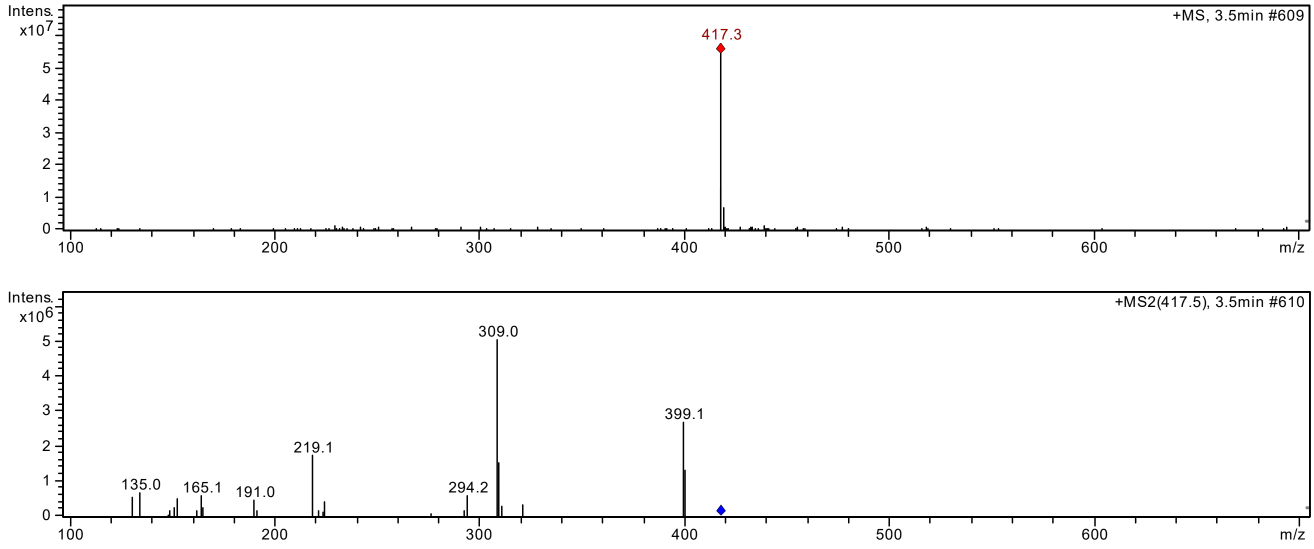

Figure S32. The MS spectrum for the compound **7b.**

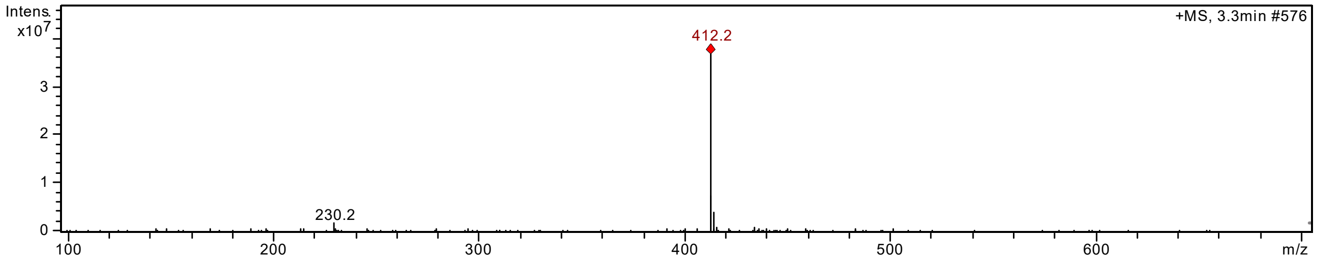

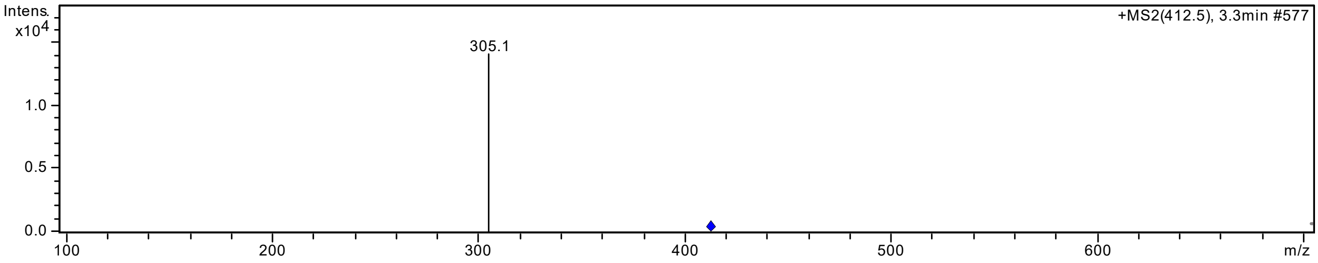

Figure S33. The MS spectrum for the compound **8b.**

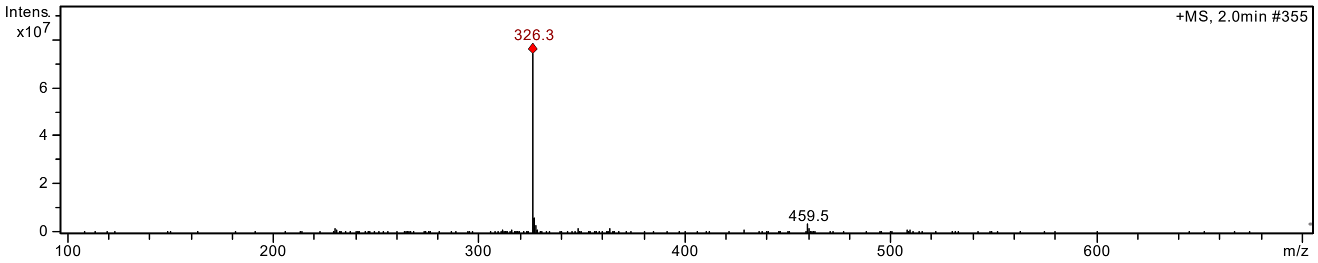

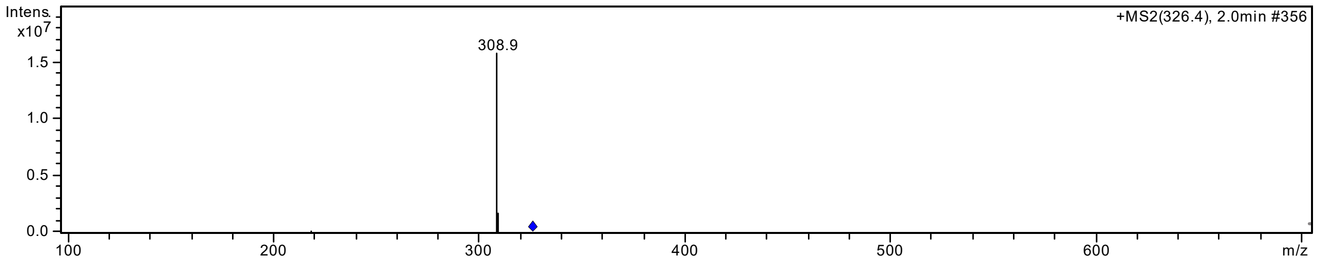

Figure S34. The MS spectrum for the compound **9b.**

1.2. The ¹H-NMR spectrum

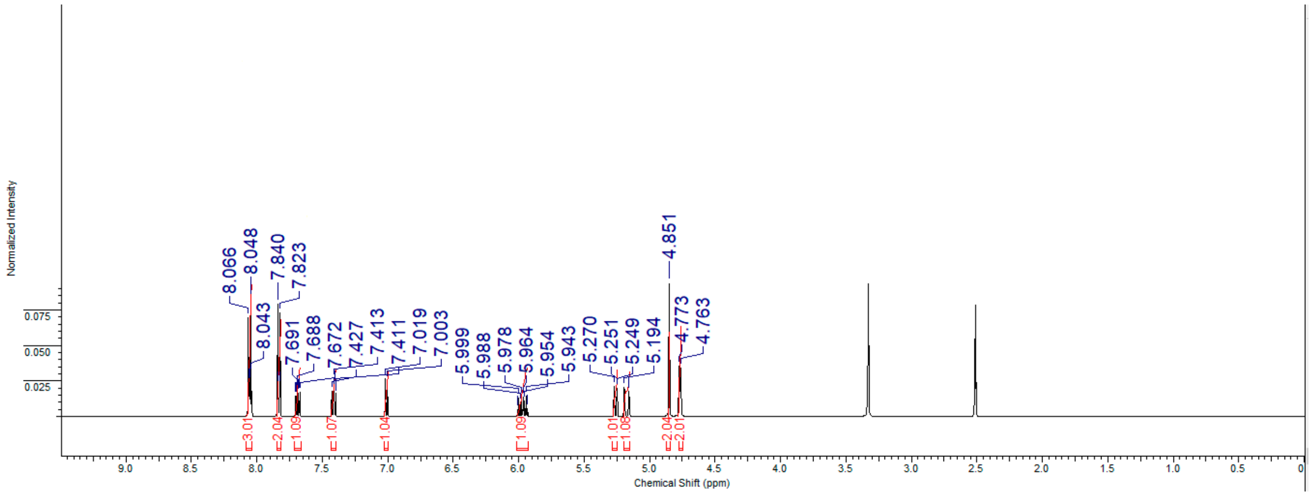

Figure S35. The ^{1}H-NMR spectrum for the compound **1a.**

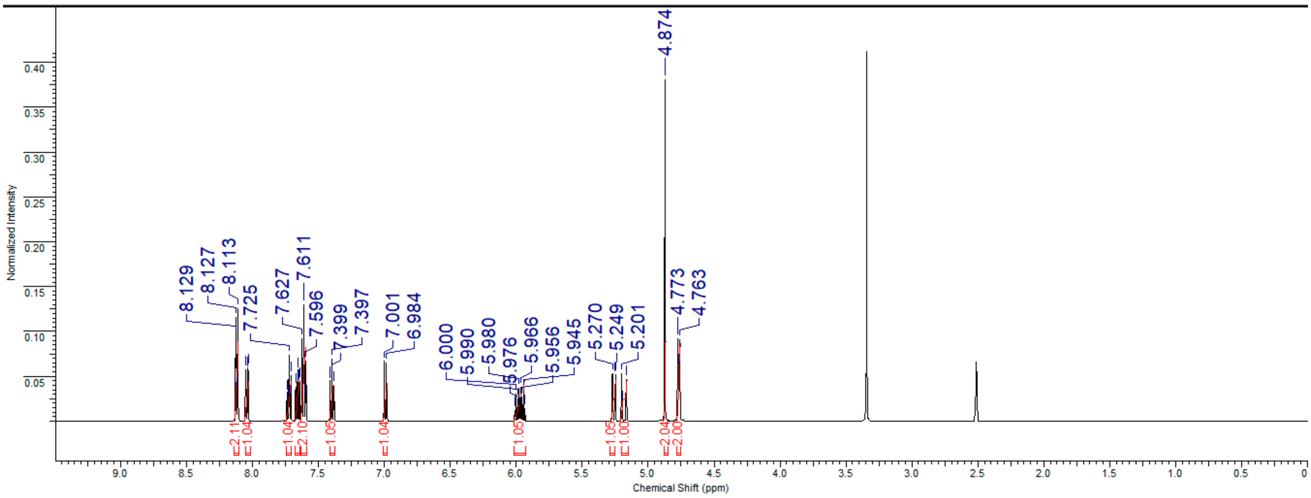

Figure S36. The ^{1}H-NMR spectrum for the compound **2a.**

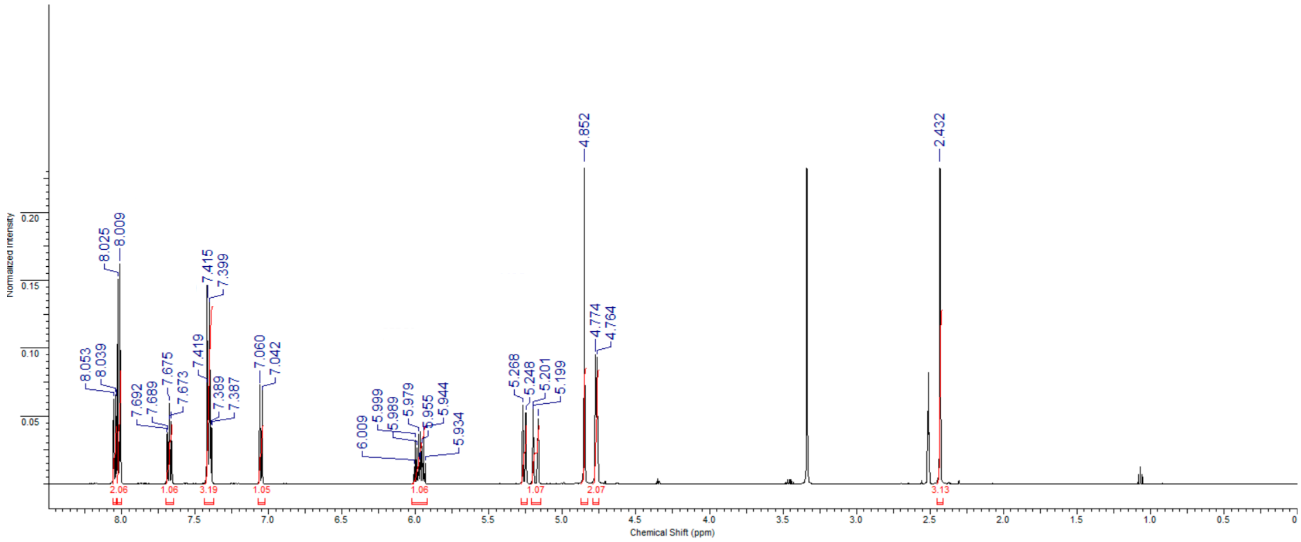

Figure S37. The ^{1}H-NMR spectrum for the compound **3a.**

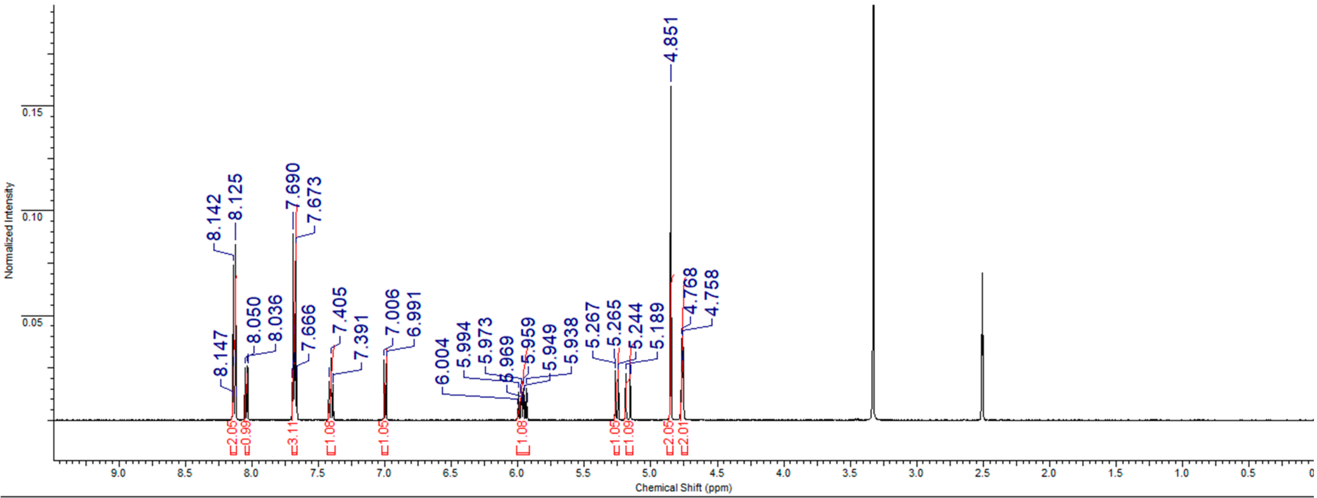

Figure S38. The ^{1}H-NMR spectrum for the compound **4a.**

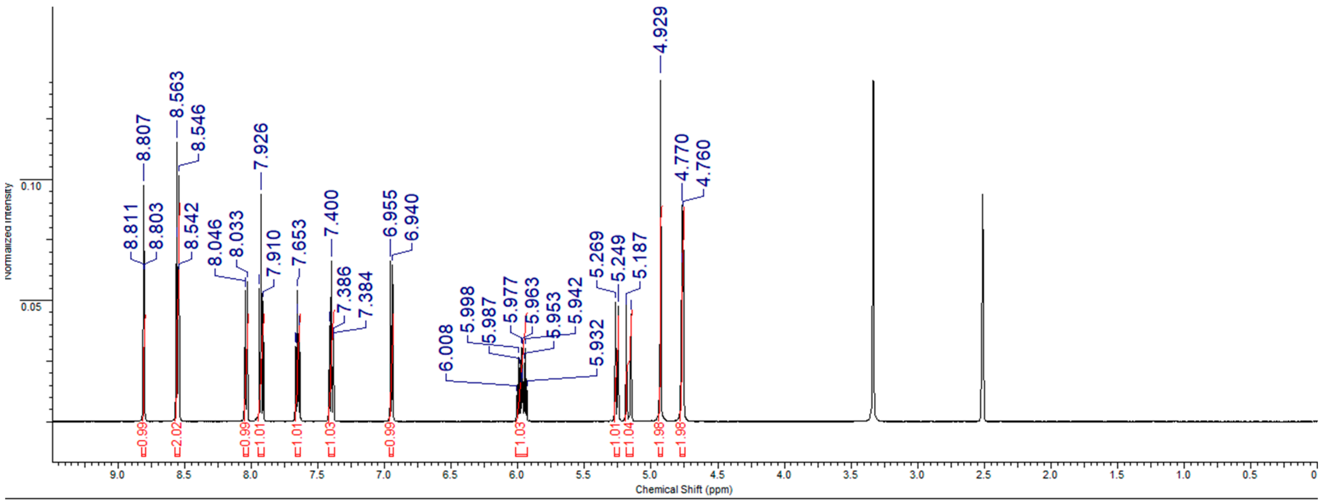

Figure S39. The ^{1}H-NMR spectrum for the compound **5a.**

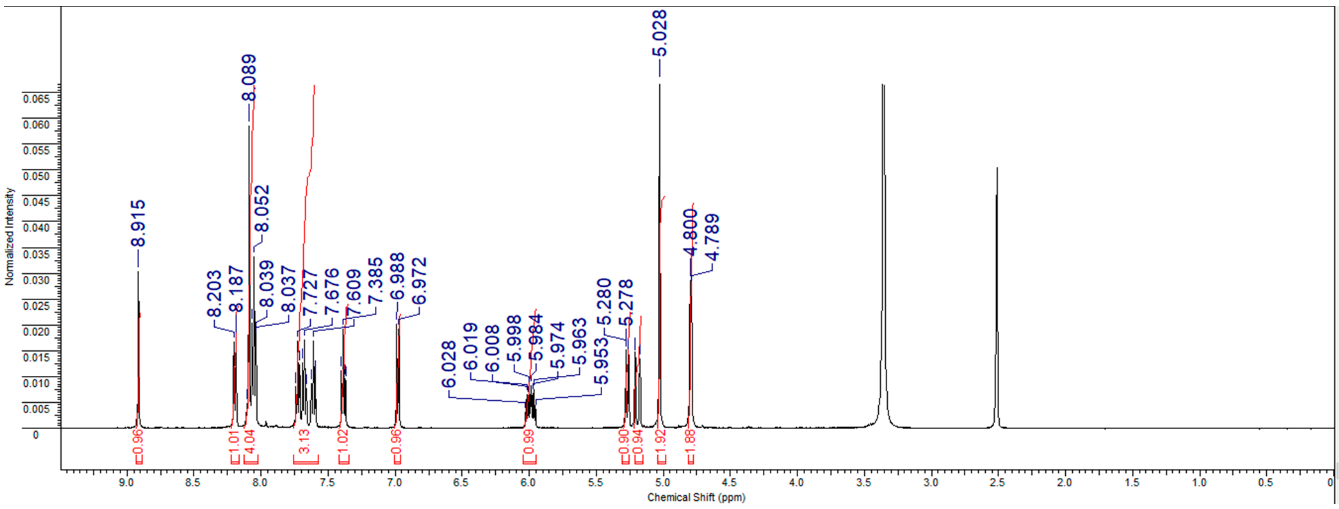

Figure S40. The ^{1}H-NMR spectrum for the compound **6a.**

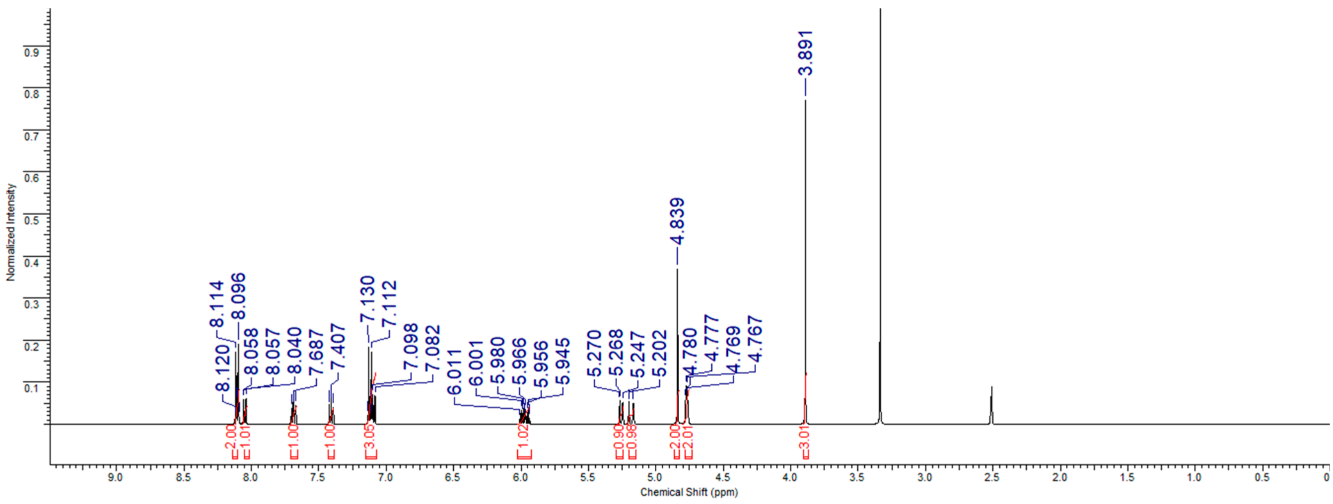

Figure S41. The ^{1}H-NMR spectrum for the compound **7a.**

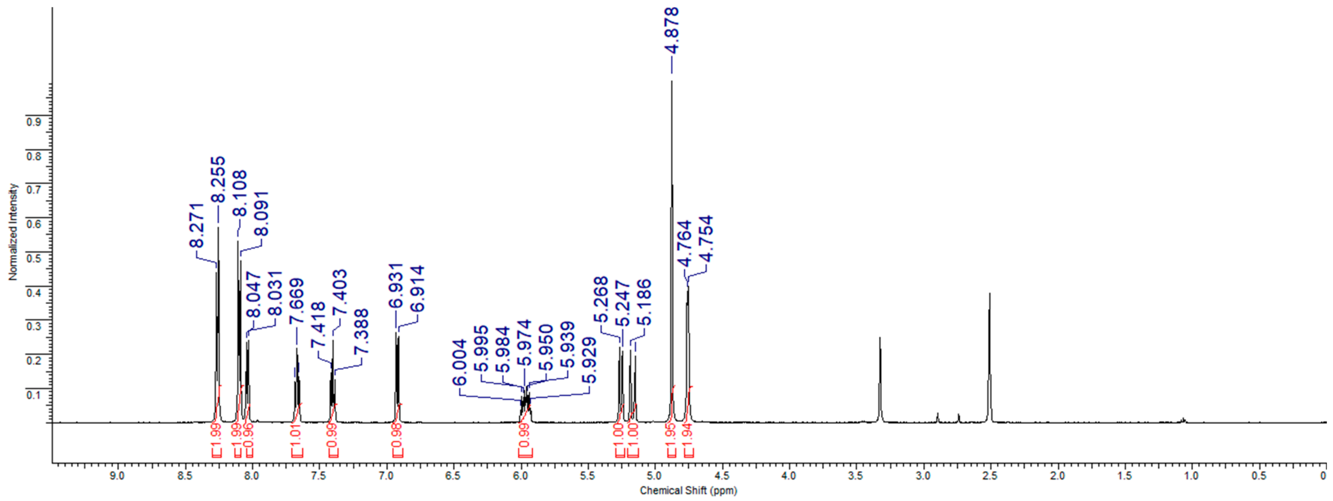

Figure S42. The ¹H-NMR spectrum for the compound **8a.**

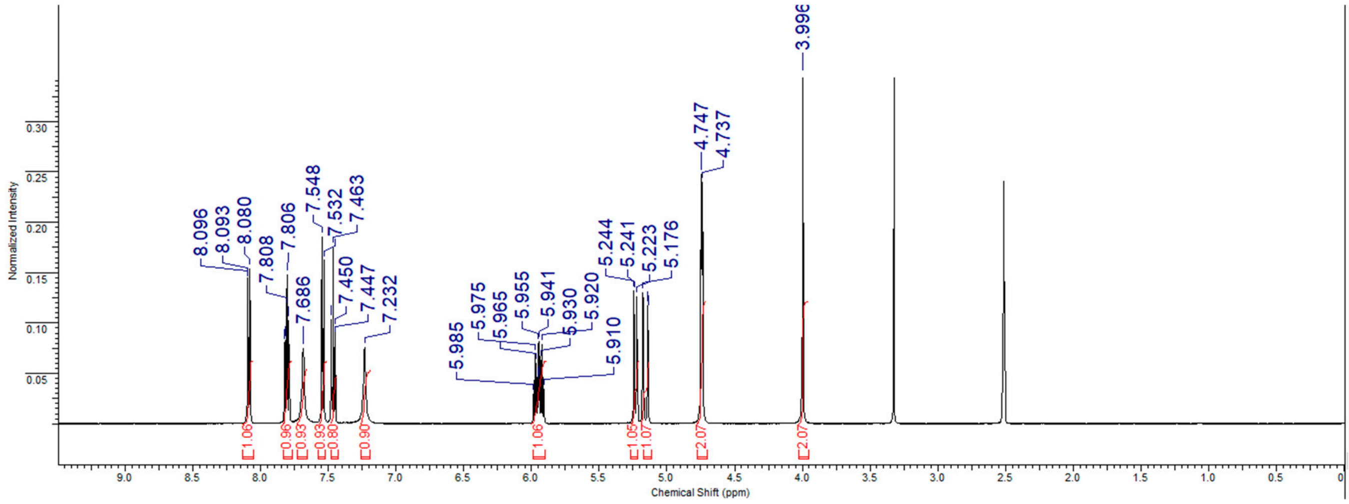

Figure S43. The ¹H-NMR spectrum for the compound **9a.**

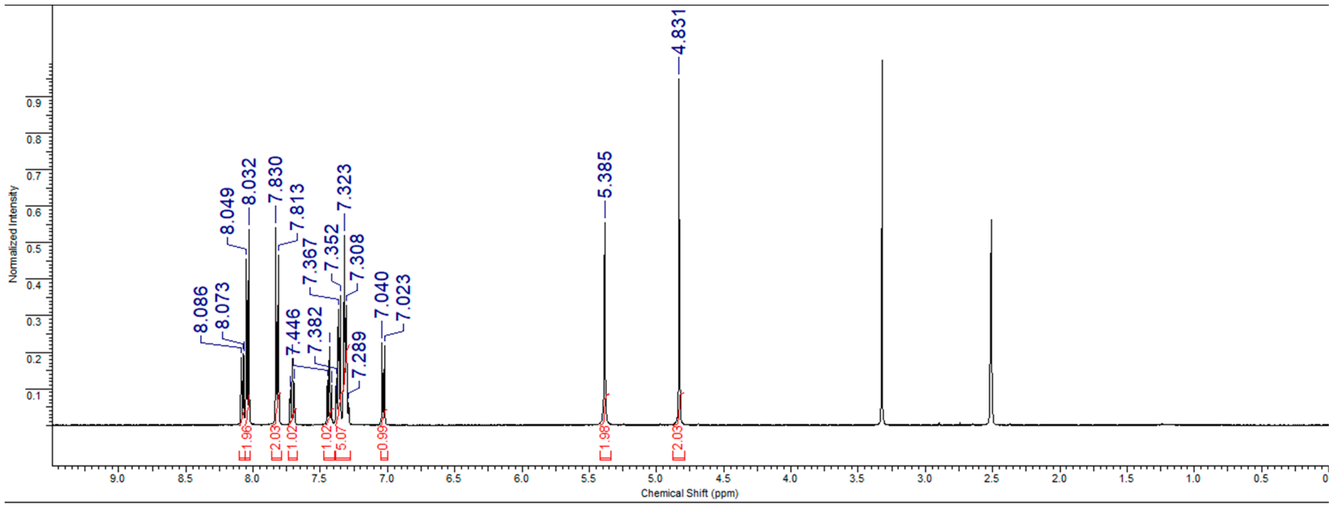

Figure S44. The ¹H-NMR spectrum for the compound **1b.**

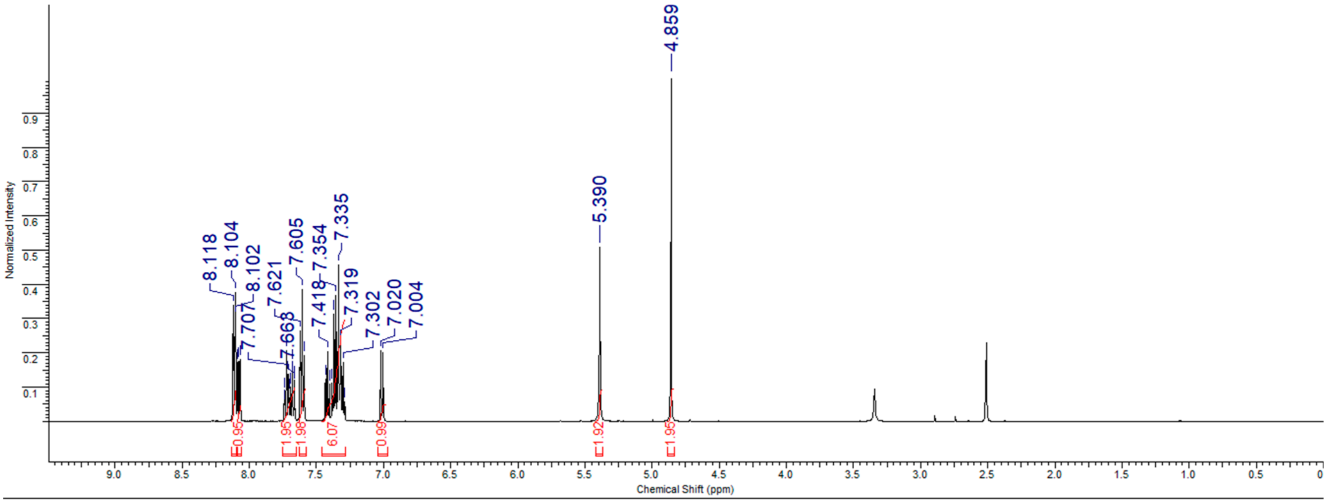

Figure S45. The ^{1}H-NMR spectrum for the compound **2b.**

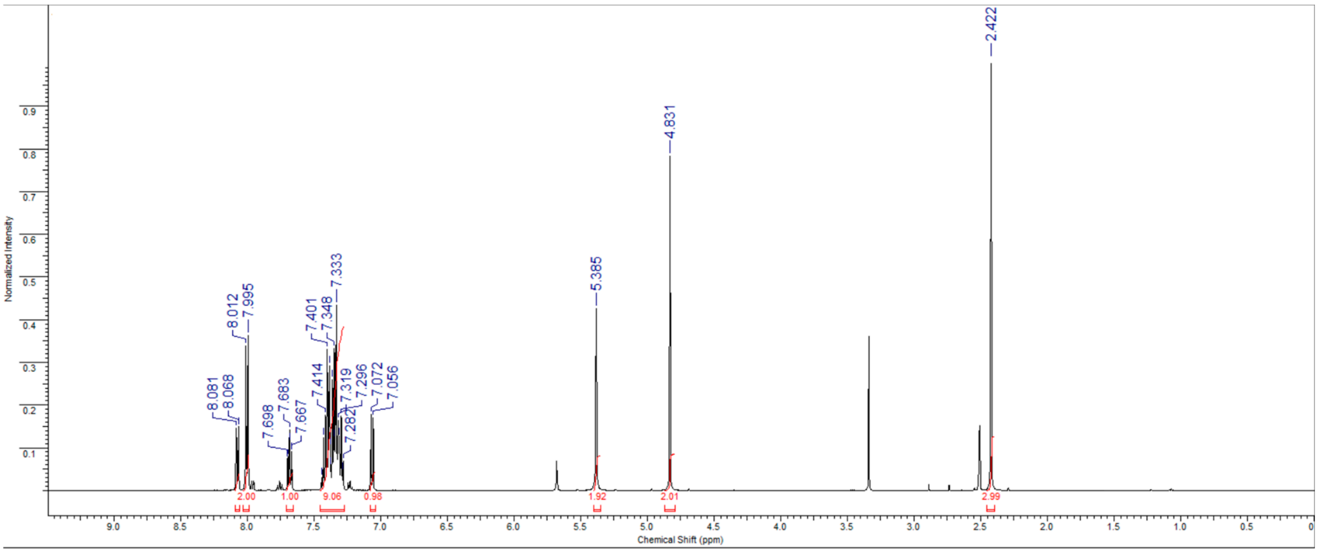

Figure S46. The ^{1}H-NMR spectrum for the compound **3b.**

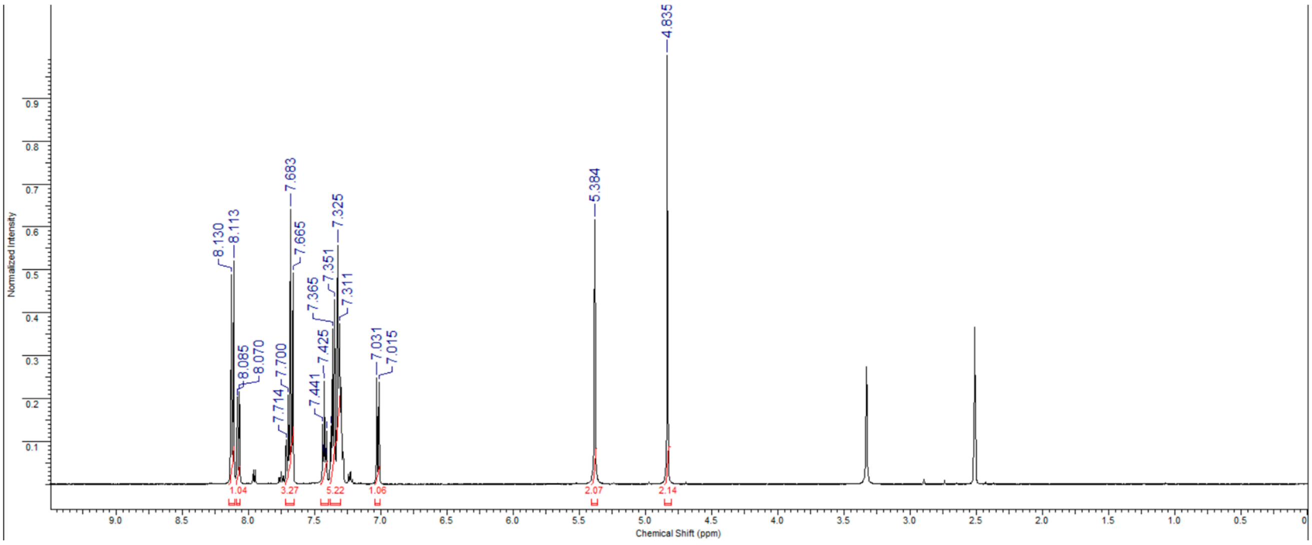

Figure S47. The ^{1}H-NMR spectrum for the compound **4b.**

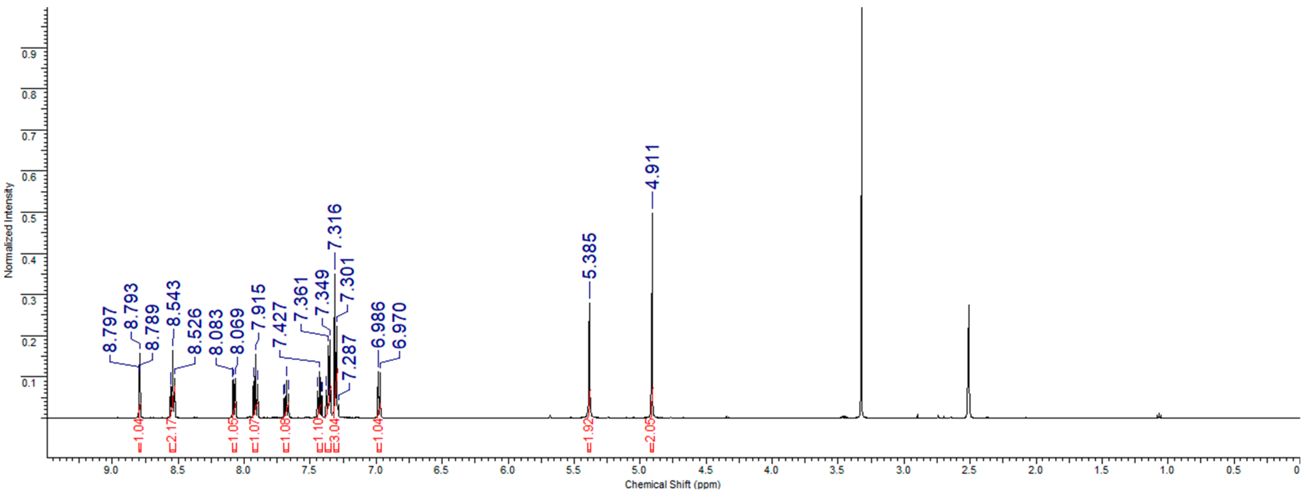

Figure S48. The ^{1}H-NMR spectrum for the compound **5b.**

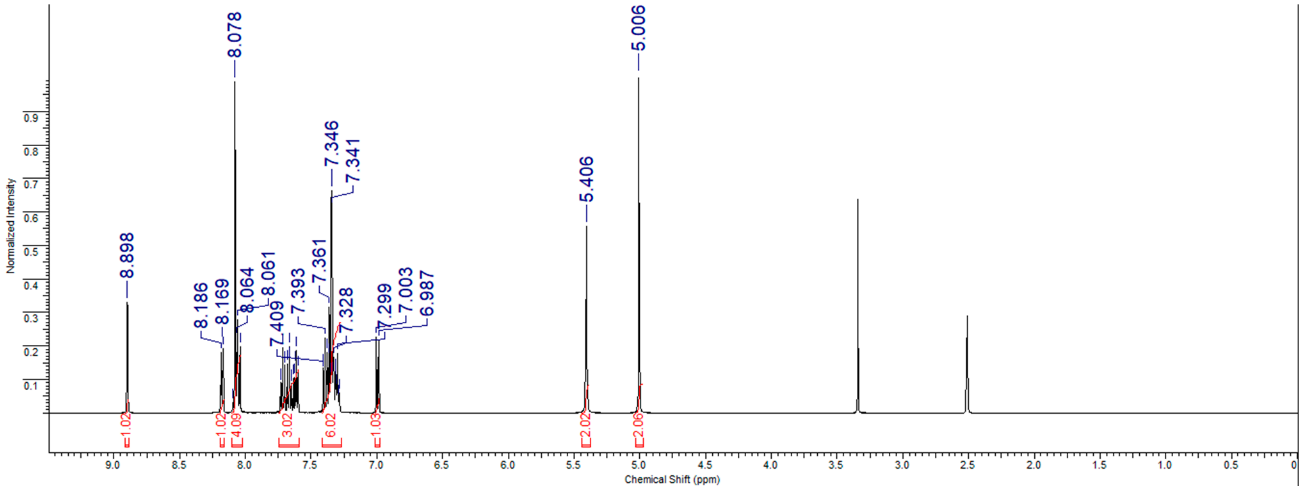

Figure S49. The ^{1}H-NMR spectrum for the compound **6b.**

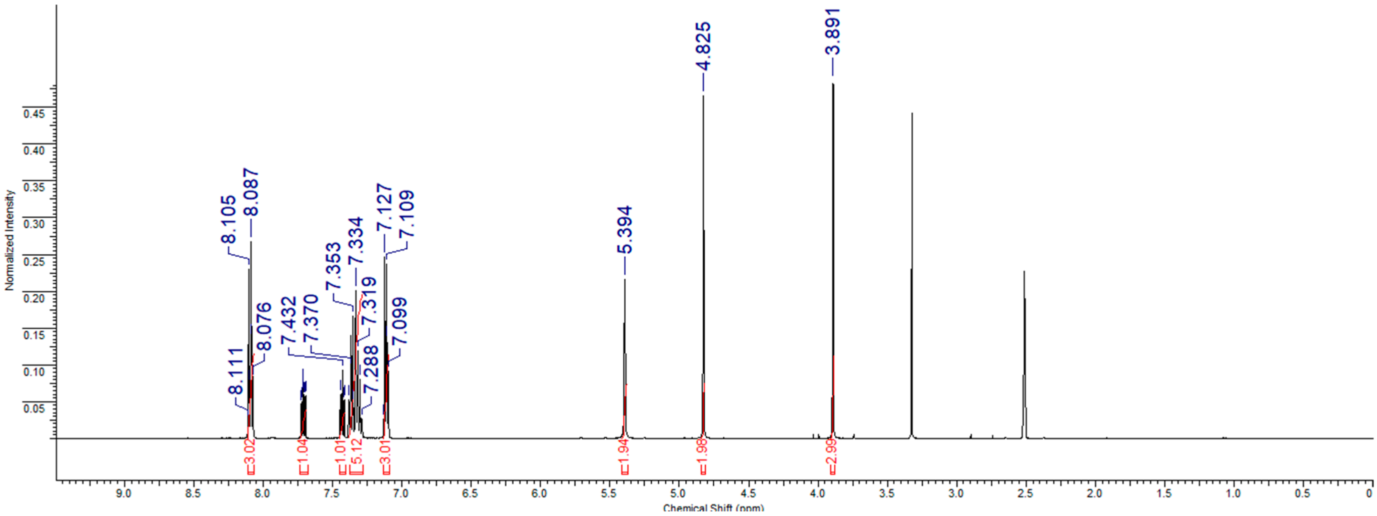

Figure S50. The ^{1}H-NMR spectrum for the compound **7b.**

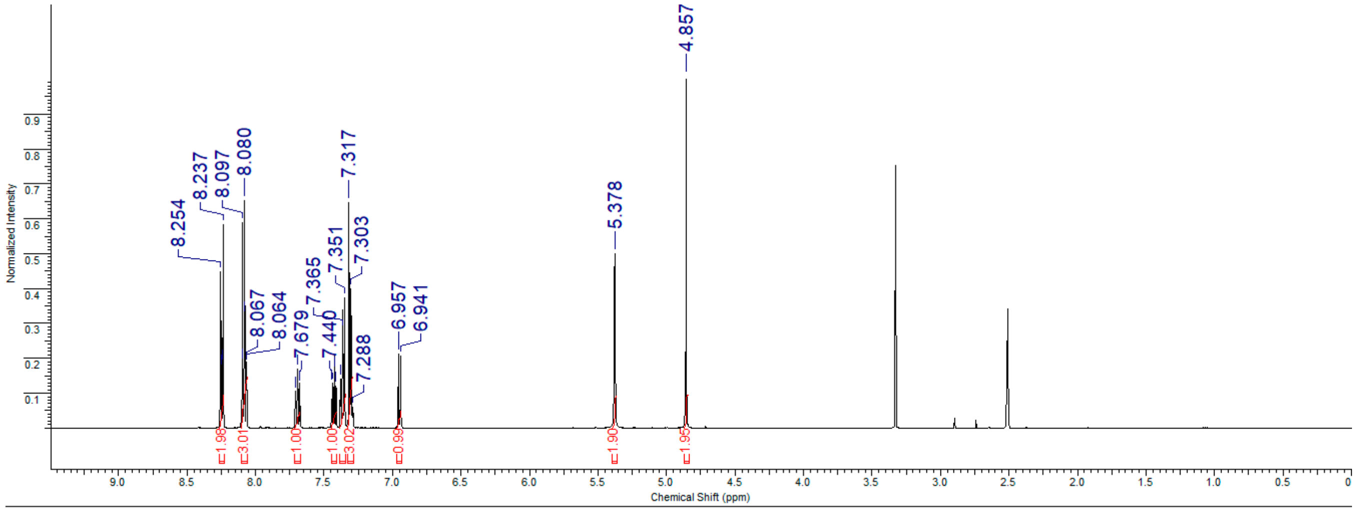

Figure S51. The ^{1}H-NMR spectrum for the compound **8b.**

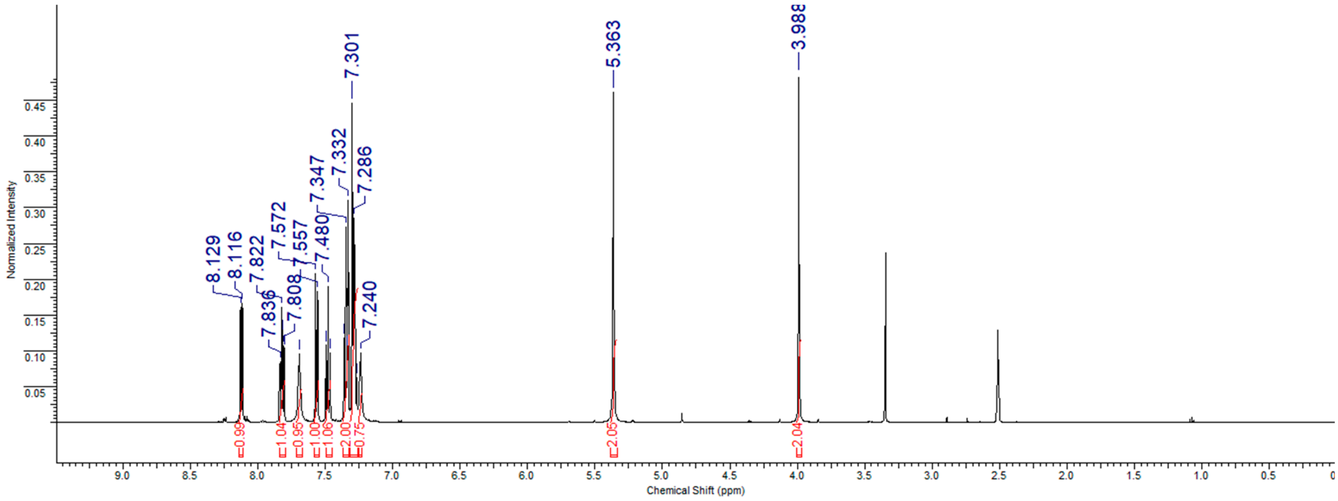

Figure S52. The ^{1}H-NMR spectrum for the compound **9b.**

1.3. The ^{13}C-NMR spectrum

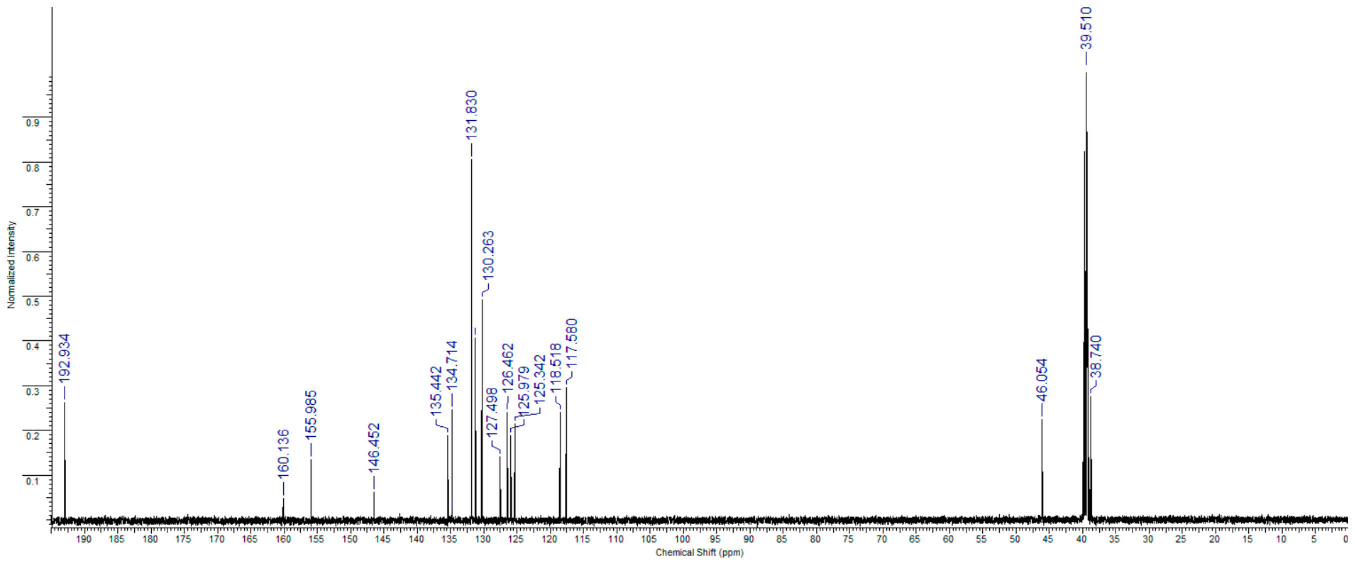

Figure S53. The ^{13}C-NMR spectrum for the compound **1a.**

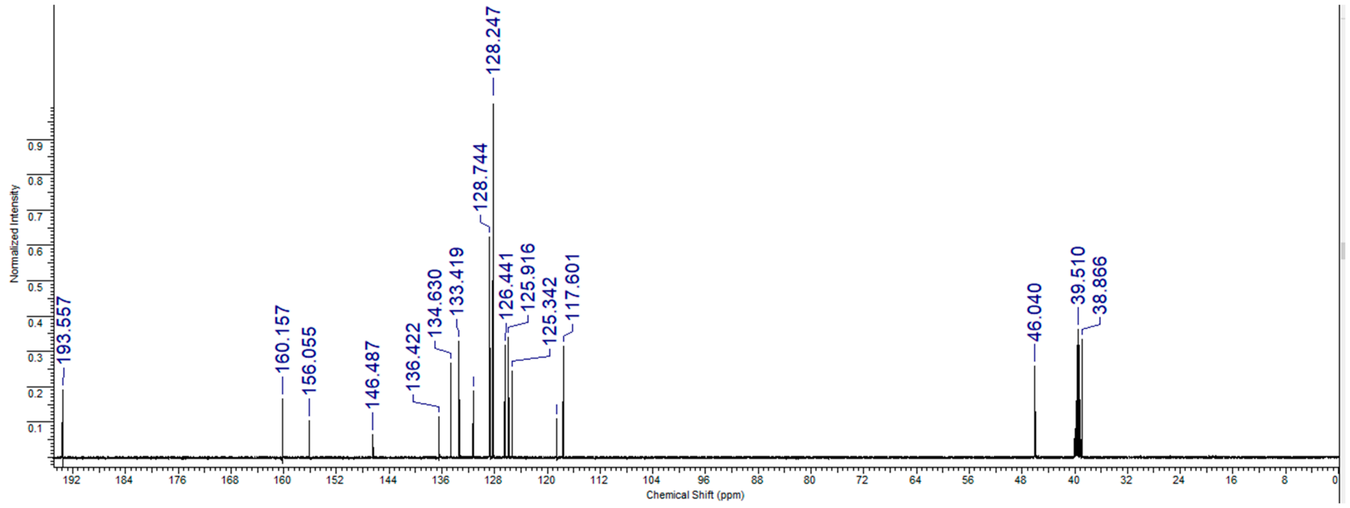

Figure S54. The ^{13}C-NMR spectrum for the compound **2a.**

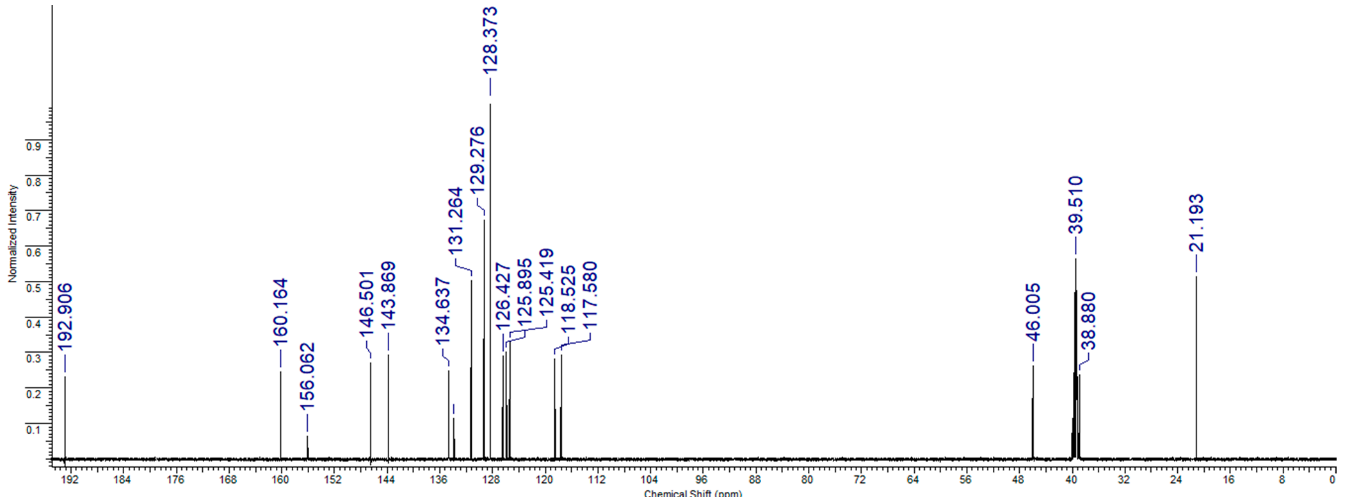

Figure S55. The ^{13}C-NMR spectrum for the compound **3a.**

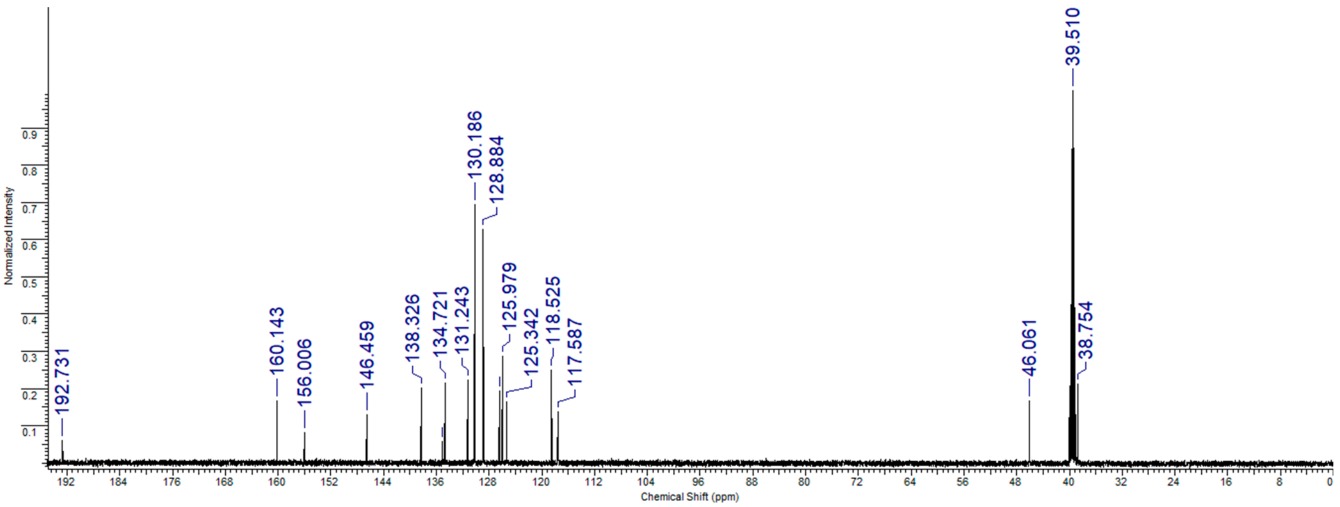

Figure S56. The ^{13}C-NMR spectrum for the compound **4a.**

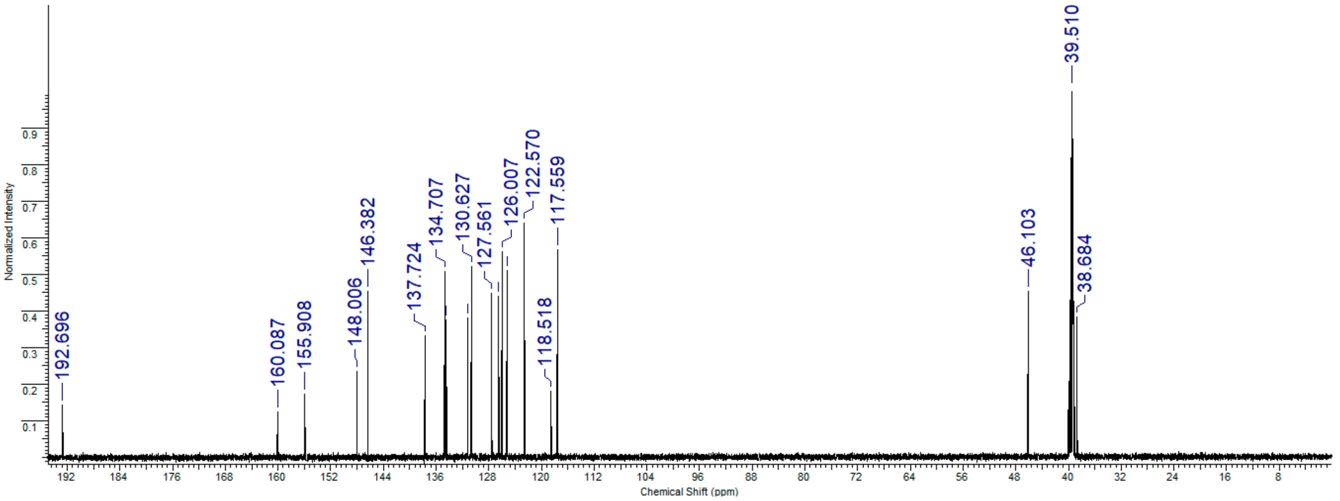

Figure S57. The ^{13}C-NMR spectrum for the compound **5a.**

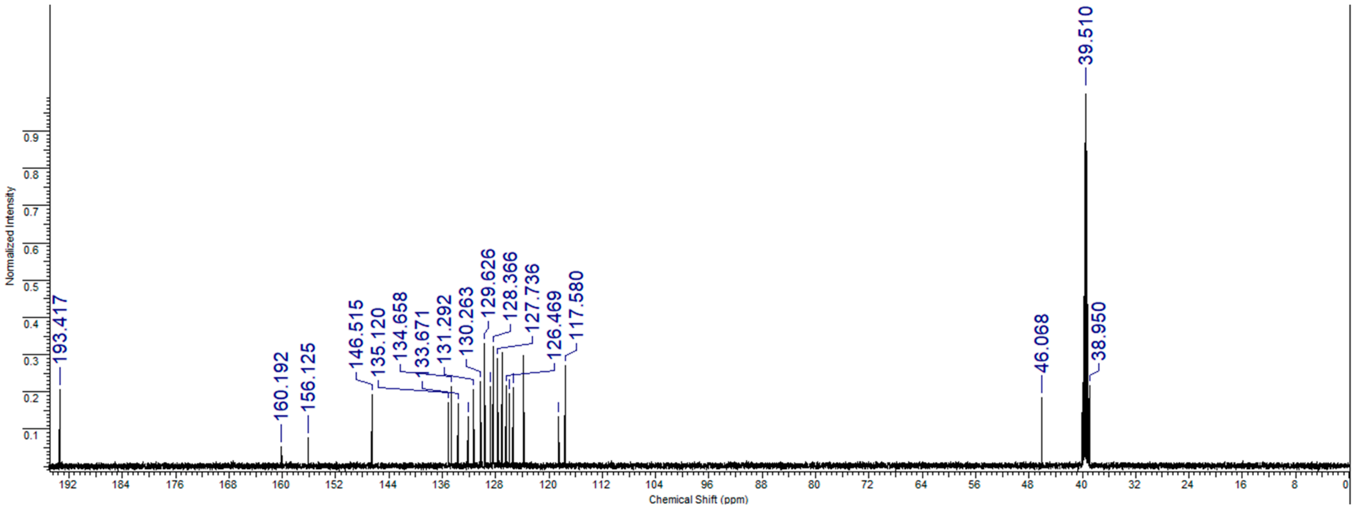

Figure S58. The ^{13}C-NMR spectrum for the compound **6a.**

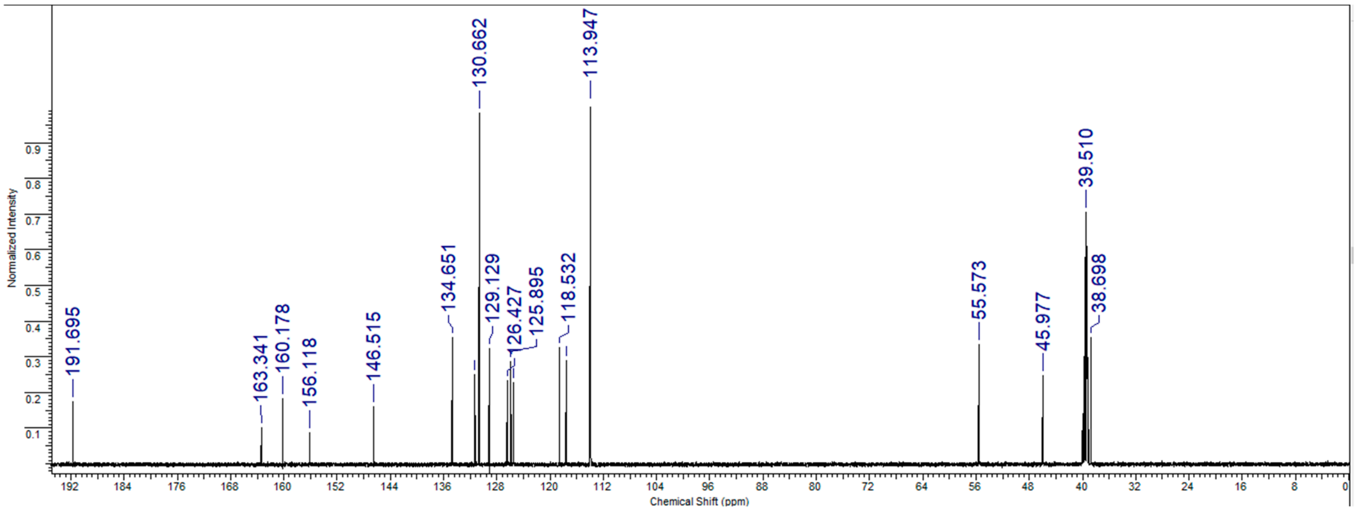

Figure S59. The ^{13}C-NMR spectrum for the compound **7a.**

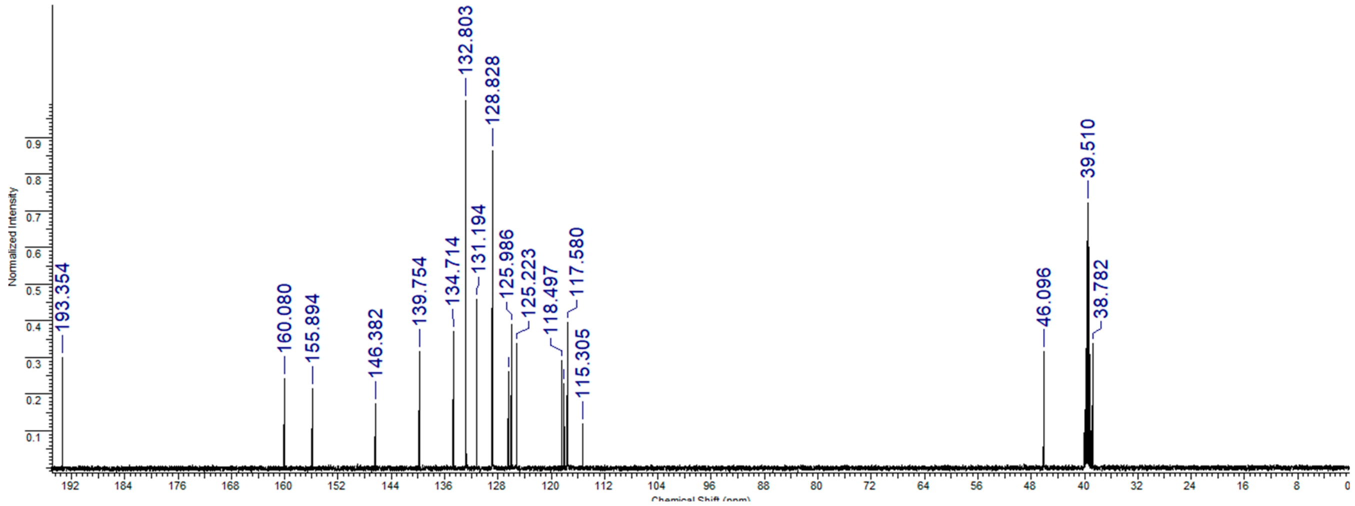

Figure S60. The ^{13}C-NMR spectrum for the compound **8a.**

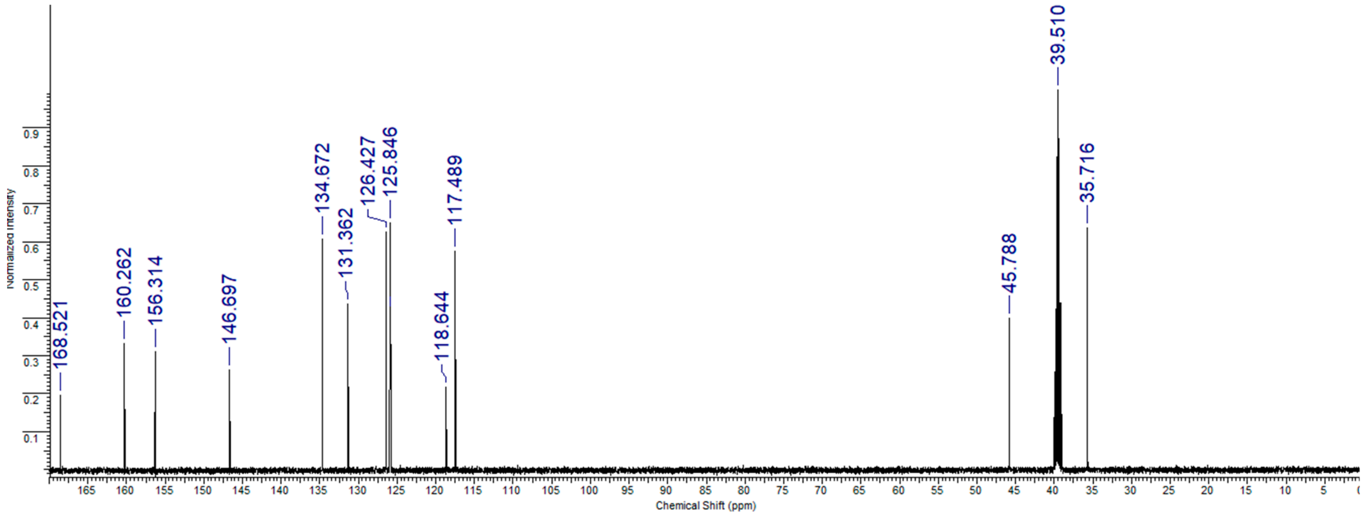

Figure S61. The ^{13}C-NMR spectrum for the compound **9a.**

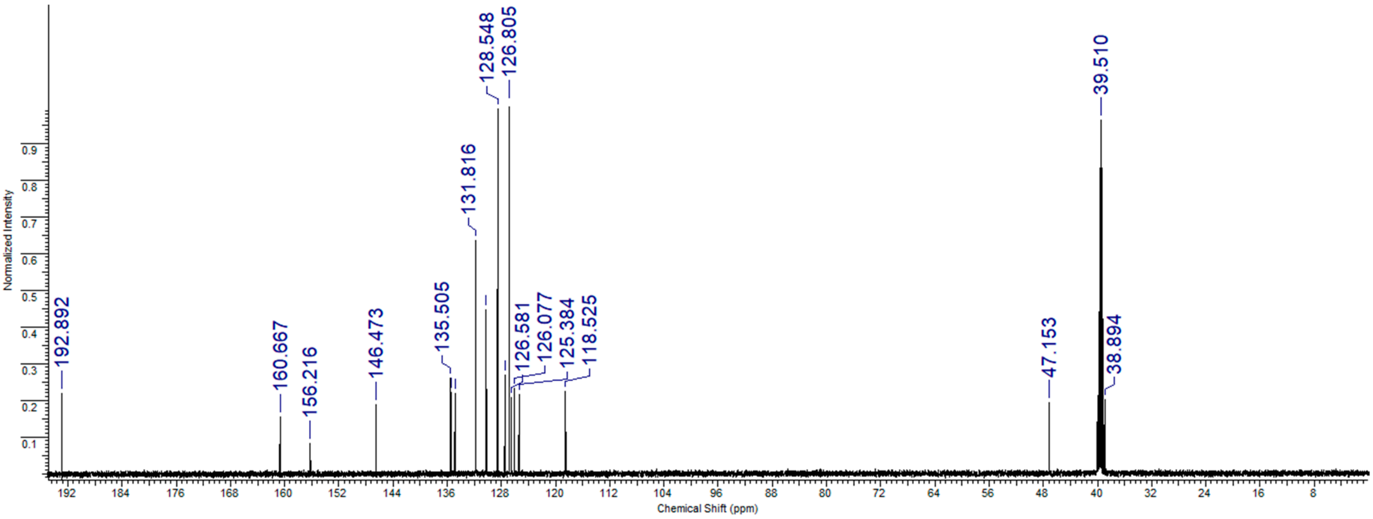

Figure S62. The ^{13}C-NMR spectrum for the compound **1b.**

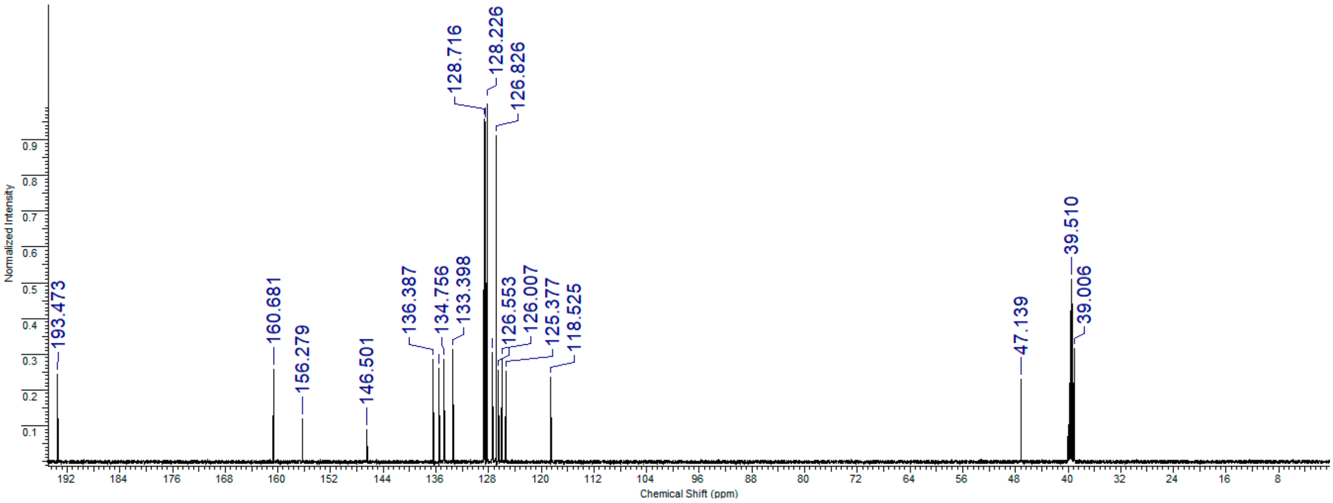

Figure S63. The ^{13}C-NMR spectrum for the compound **2b.**

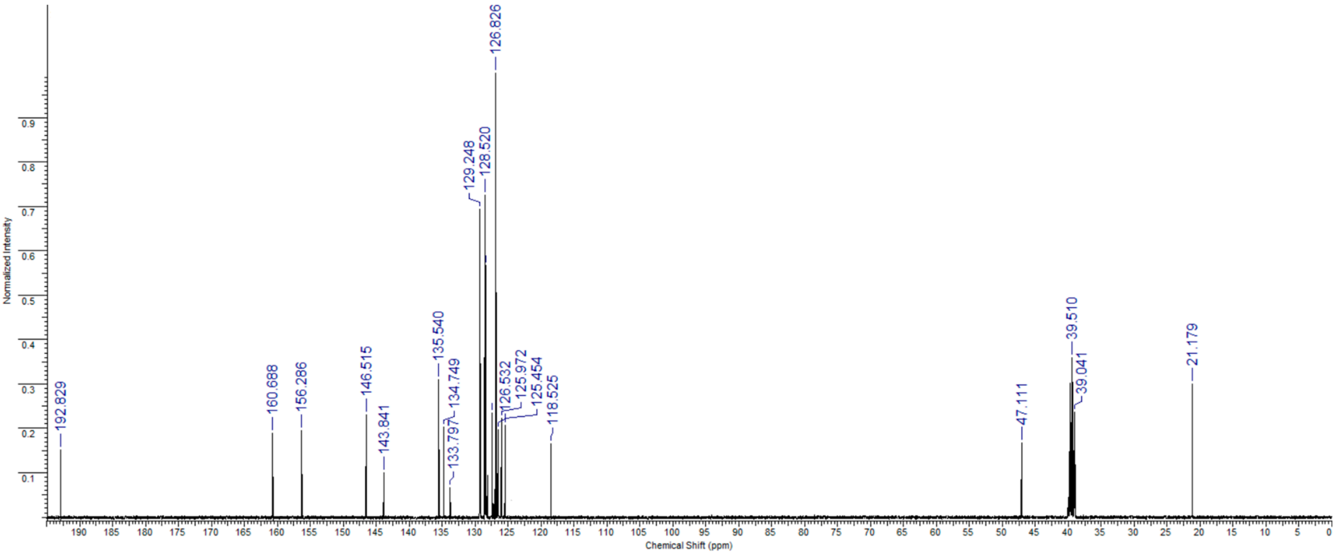

Figure S64. The ^{13}C-NMR spectrum for the compound **3b.**

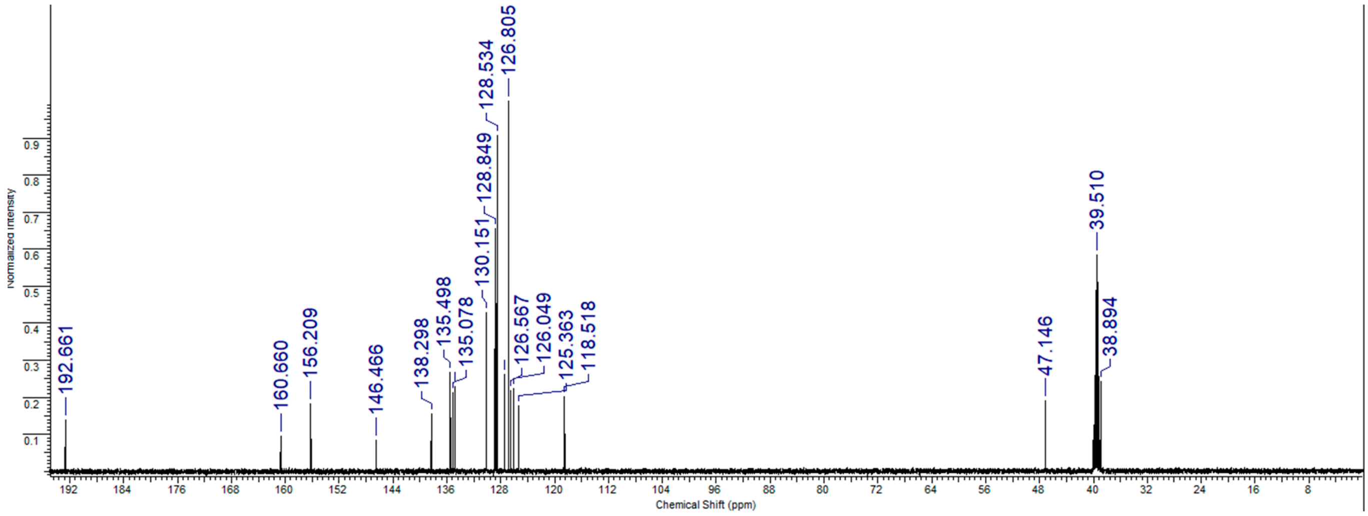

Figure S65. The ^{13}C-NMR spectrum for the compound **4b.**

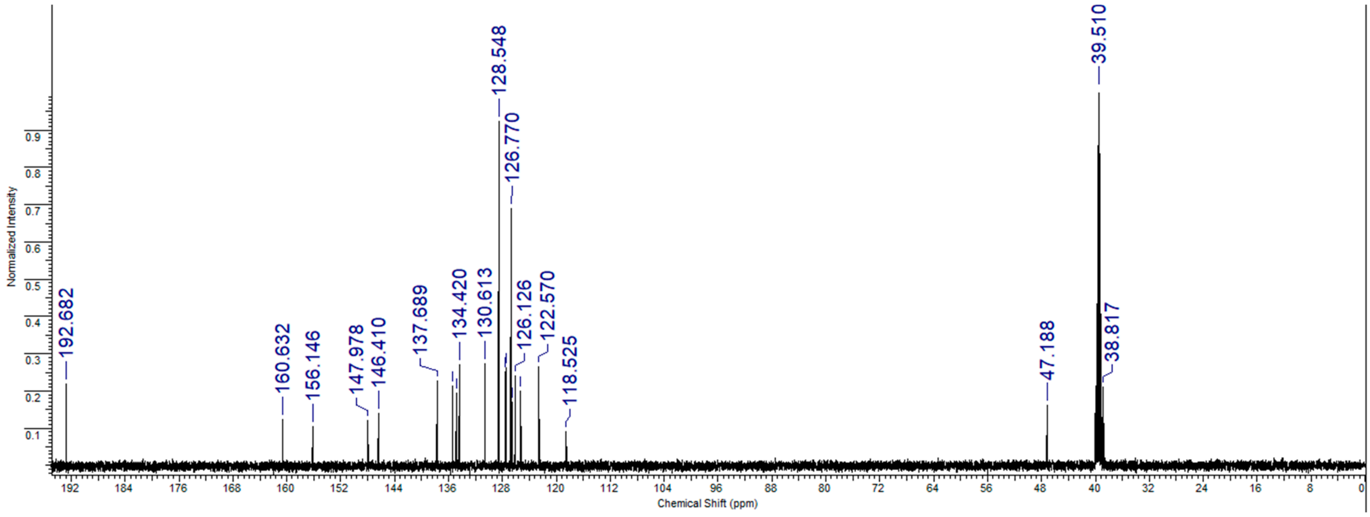

Figure S66. The ^{13}C-NMR spectrum for the compound **5b.**

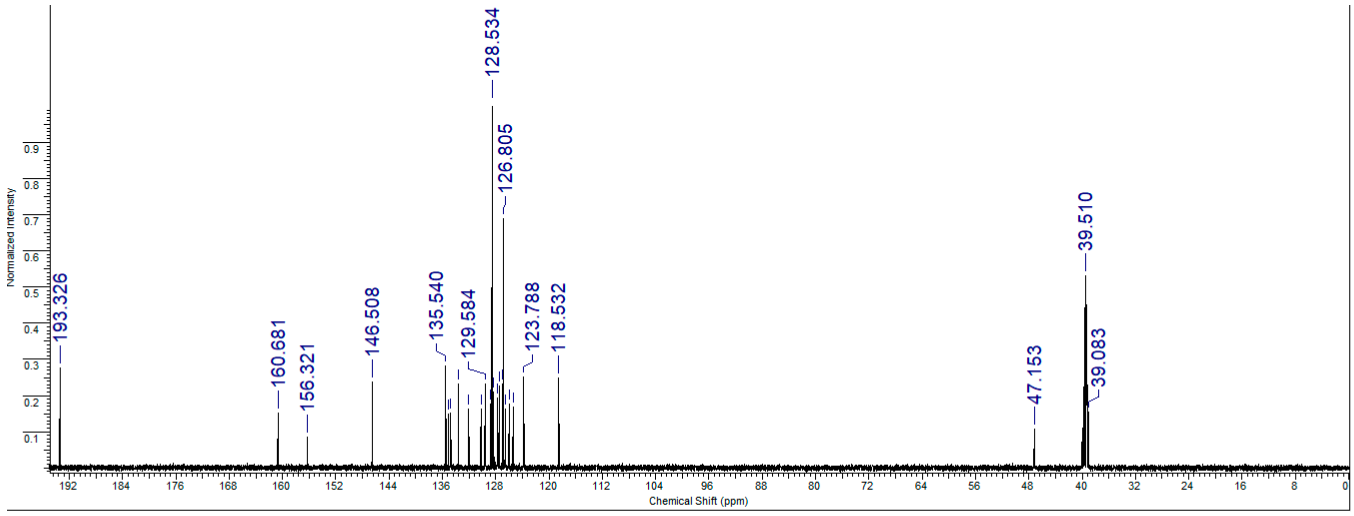

Figure S67. The ^{13}C-NMR spectrum for the compound **6b.**

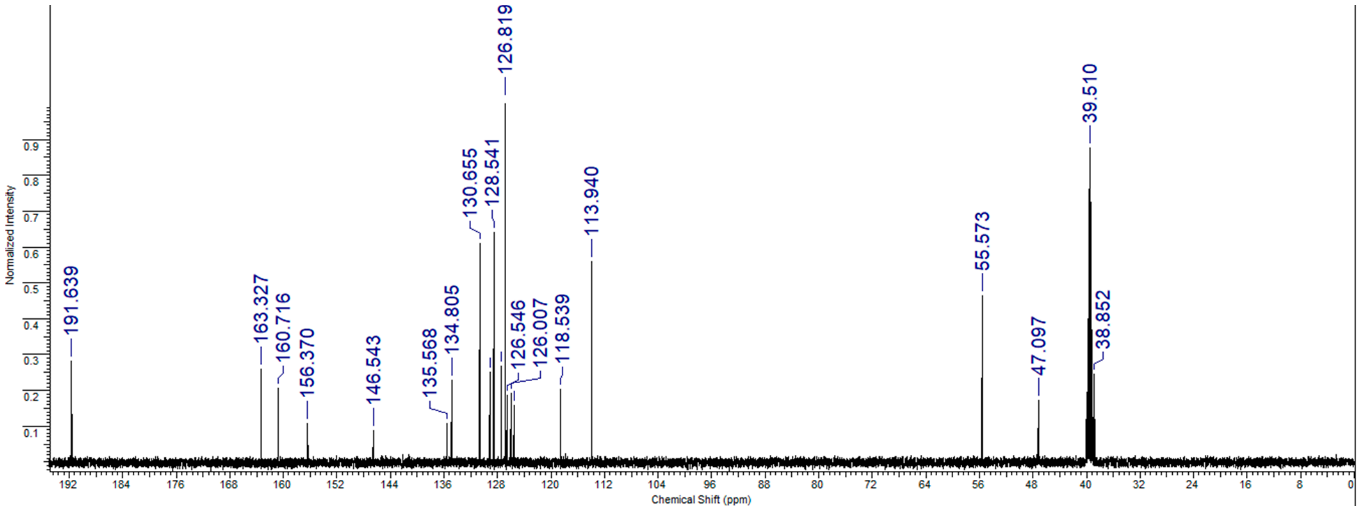

Figure S68. The ^{13}C-NMR spectrum for the compound **7b.**

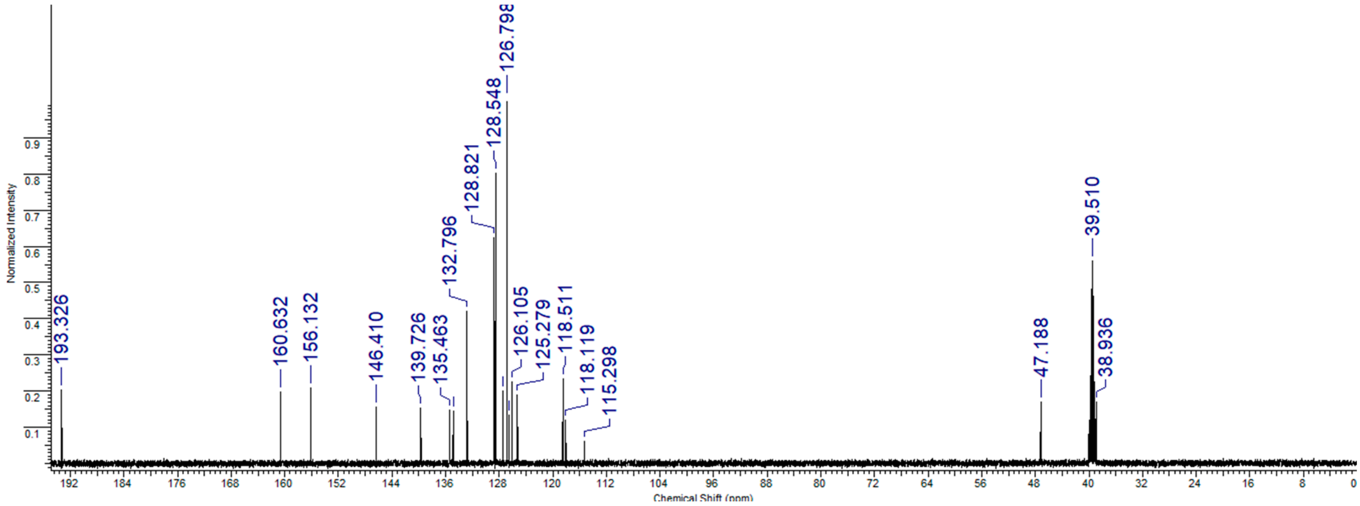

Figure S69. The ^{13}C-NMR spectrum for the compound **8b.**

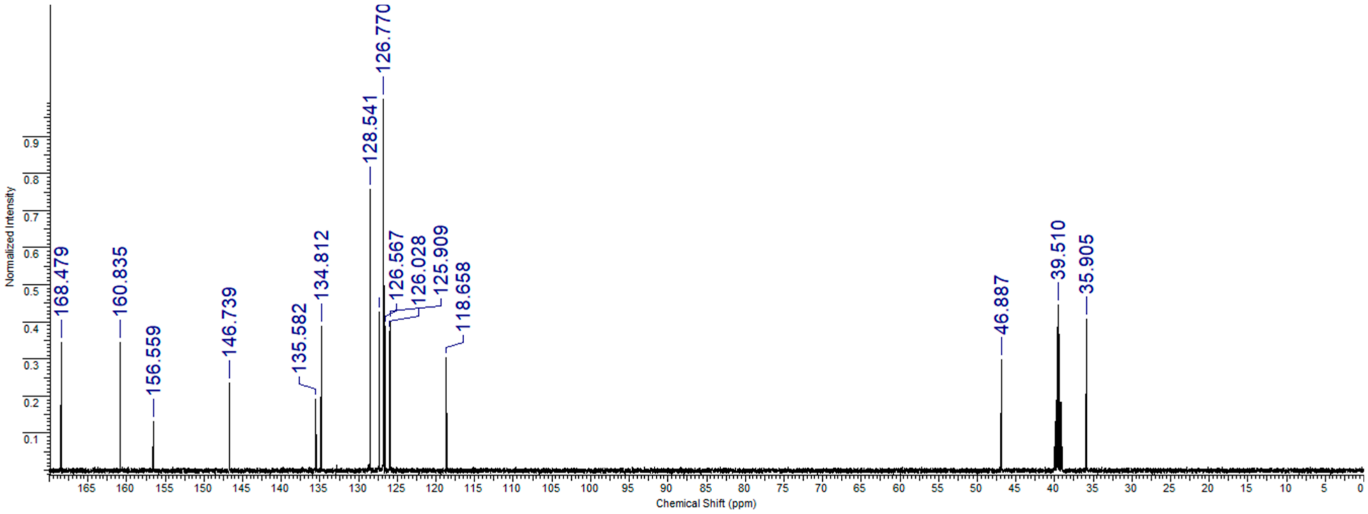

Figure S70. The ^{13}C-NMR spectrum for the compound **9b.**

1.4. Molecular docking

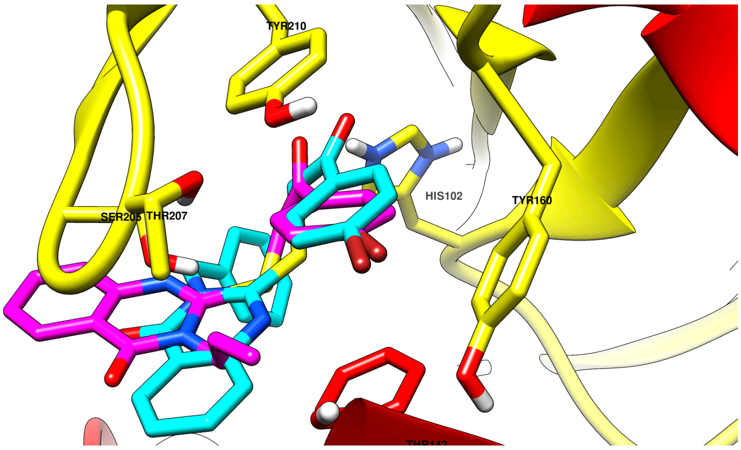

Figure S71. The best binding pose for the compounds **1a** and **1b** (carbon atoms in magenta) in the benzodiazepine binding site of GABA$_A$ receptor. The $\gamma 2$ subunit is depicted in red, while $\alpha 1$ subunit is depicted in yellow.

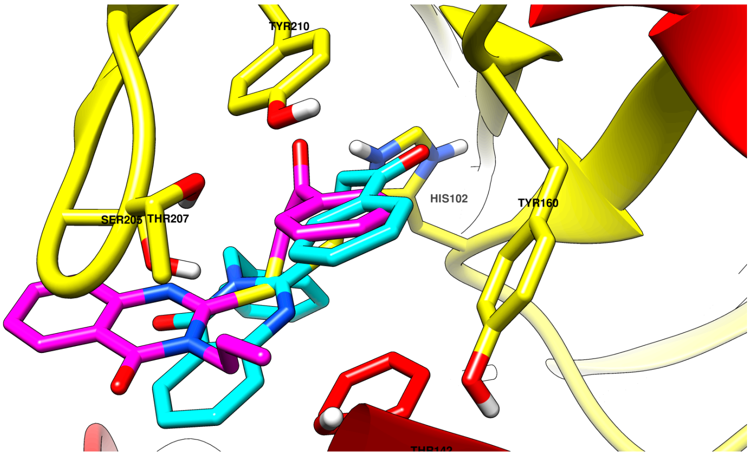

Figure S72. The best binding pose for the compounds **2a** and **2b** (carbon atoms in magenta) in the benzodiazepine binding site of GABA$_A$ receptor. The $\gamma 2$ subunit is depicted in red, while $\alpha 1$ subunit is depicted in yellow.

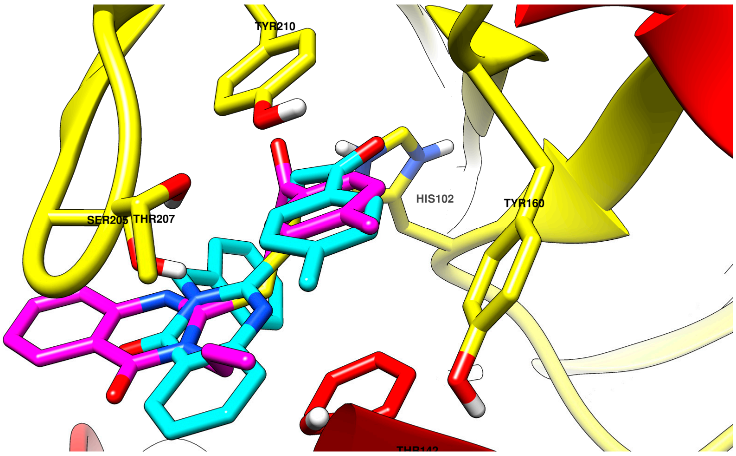

Figure S73. The best binding pose for the compounds 3**a** and **3b** (carbon atoms in magenta) in the benzodiazepine binding site of GABA$_A$ receptor. The $\gamma 2$ subunit is depicted in red, while $\alpha 1$ subunit is depicted in yellow.

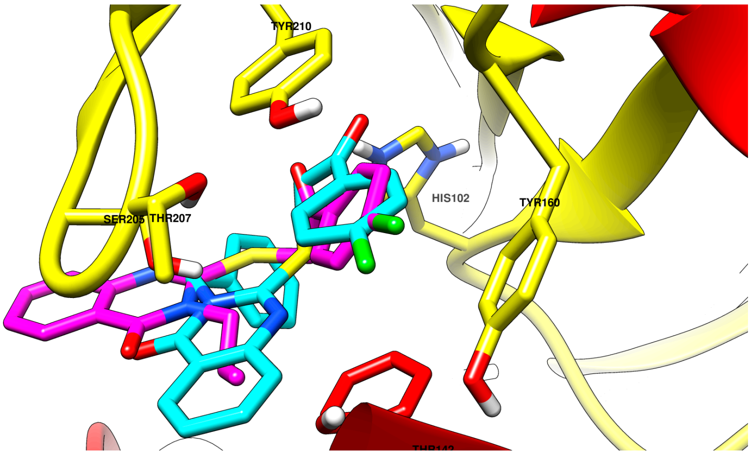

Figure S74. The best binding pose for the compounds **4a** and **4b** (carbon atoms in magenta) in the benzodiazepine binding site of GABAA receptor. The γ2 subunit is depicted in red, while α1 subunit is depicted in yellow.

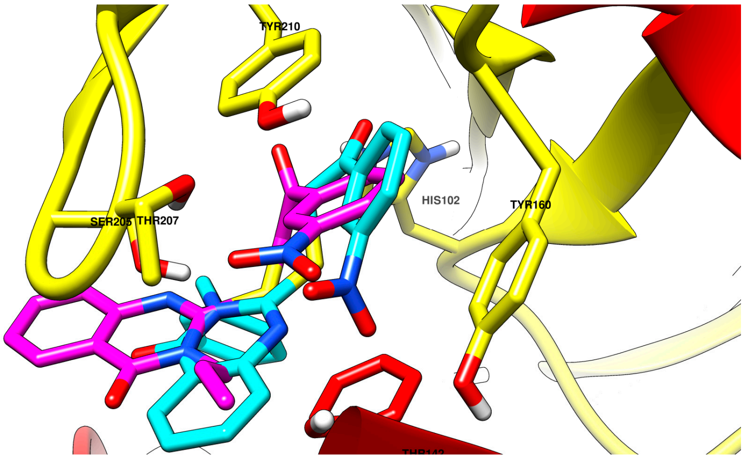

Figure S75. The best binding pose for the compounds **5a** and **5b** (carbon atoms in magenta) in the benzodiazepine binding site of GABAA receptor. The γ2 subunit is depicted in red, while α1 subunit is depicted in yellow.

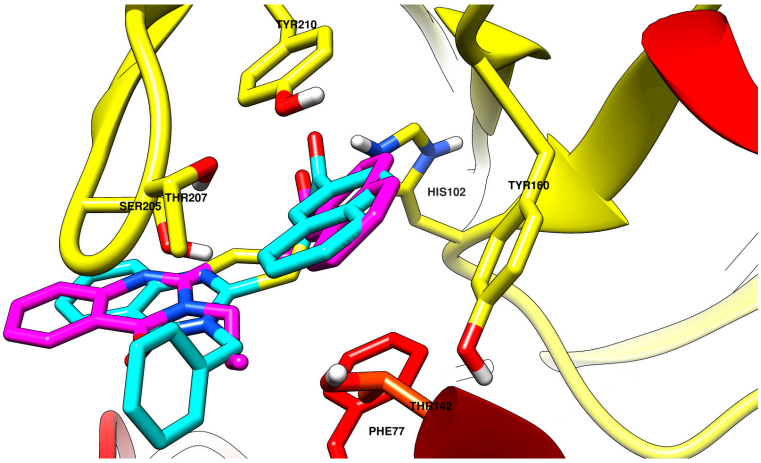

Figure S76. The best binding pose for the compounds **6a** and **6b** (carbon atoms in magenta) in the benzodiazepine binding site of GABAA receptor. The γ2 subunit is depicted in red, while α1 subunit is depicted in yellow.

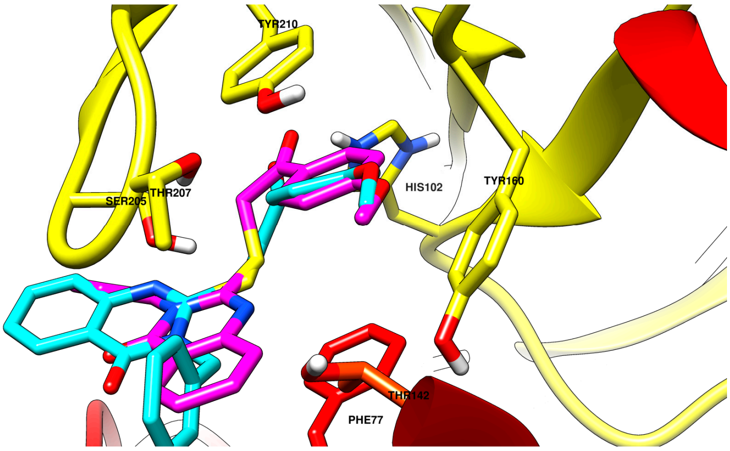

Figure S77. The best binding pose for the compounds **7a** and **7b** (carbon atoms in magenta) in the benzodiazepine binding site of GABAA receptor. The γ2 subunit is depicted in red, while α1 subunit is depicted in yellow.

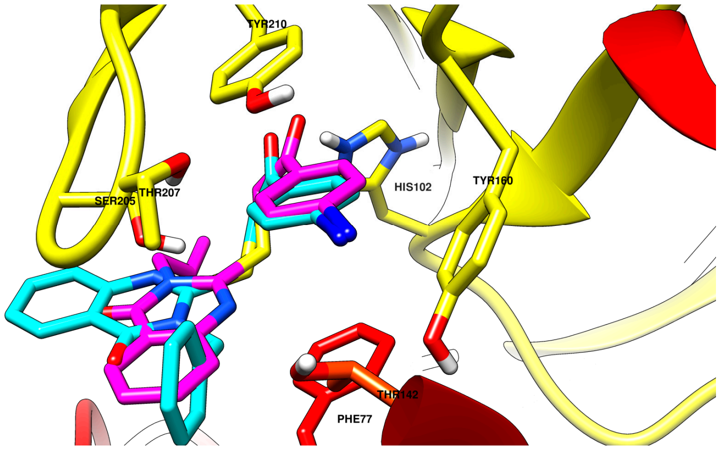

Figure S78. The best binding pose for the compounds **8a** and **8b** (carbon atoms in magenta) in the benzodiazepine binding site of GABAA receptor. The γ2 subunit is depicted in red, while α1 subunit is depicted in yellow.

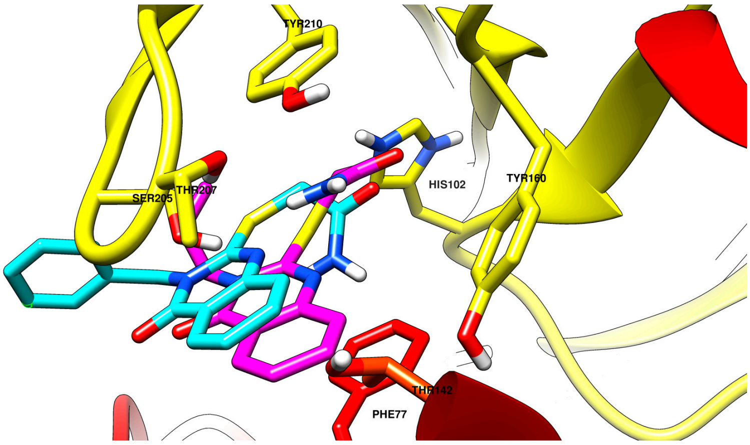

Figure S79. The best binding pose for the compounds **9a** and **9b** (carbon atoms in magenta) in the benzodiazepine binding site of GABAA receptor. The γ2 subunit is depicted in red, while α1 subunit is depicted in yellow.

1.5. Molecular dynamics simulation

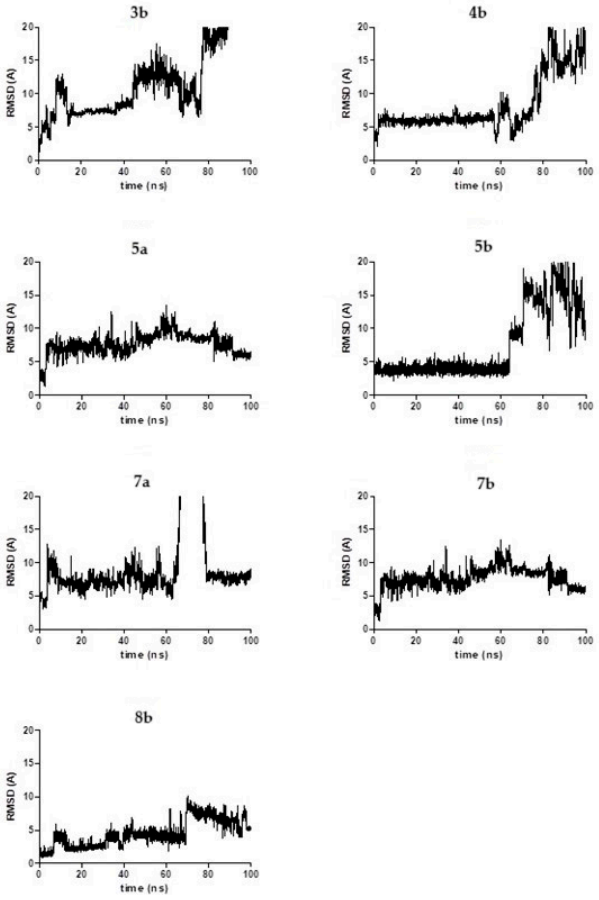

Figure S80. RMSD (A) of the ligands during the 100 ns simulation in complex with carbonic anhydrase II for the compounds **3b**, **4b**, **5a**, **5b**, **7a**, **7b**, **8a** and **8b**.

2. Tables

2.1. Pentylenetetrazole (PTZ)-induced seizures

Table S1. Protection against PTZ-induced seizure model, time until the first seizure, mean number of seizures and LogP, for the compounds of the "**a**" series, in the first 30 minutes after PTZ administration.

**Comp.**	**Doses (mg/kg)**	**PTZ P/T[a] (% protection)**	**T*c* (s)**	**No. seizures[b]**	**Log P**
**NC**	-	0/6 (0%)	111.67 ± 27.38	9.33 ± 1.65	-
**1a**	D_1	0/6 (0%)	398.33 ± 40.37	7.83 ± 2.09	3.56
	D_2	1/6 (16.67%)	660.00 ± 248.35	2.00 ± 0.68	
	D_3	3/6 (50%)	1600.00*** ± 143.39	2.00 ± 1.18	
**2a**	D_1	0/6 (0%)	630.00 ± 45.83	13.17 ± 3.75	3.16
	D_2	0/6 (0%)	1210.00*** ± 118.41	6.00 ± 1.46	
	D_3	3/6 (50%)	1130.00*** ± 307.66	4.83 ± 2.48	
**3a**	D_1	1/6 (16.67%)	590.00 ± 242.69	8.33 ± 2.26	3.21
	D_2	1/6 (16.67%)	600.00 ± 253.61	5.17 ± 1.05	
	D_3	0/6 (0%)	590.00 ± 170.70	9.67 ± 3.06	
**4a**	D_1	0/6 (0%)	671.67 ± 156.77	4.00 ± 0.52	3.46
	D_2	0/6 (0%)	153.33 ± 21.08	4.00 ± 1.06	
	D_3	0/6 (0%)	690.00 ± 33.76	7.83 ± 1.11	
**5a**	D_1	0/6 (0%)	1080.00** ± 63.87	9.83 ± 1.74	2.65

Comp.	Doses (mg/kg)	PTZ P/T[a] (% protection)	Tc (s)	No. seizures[b]	Log P
	D_2	0/6 (0%)	1030.00** ± 78.10	15.67 ± 3.52	
	D_3	3/6 (50%)	1800.00*** ± 0.00	0.83* ± 0.40	
**6a**	D_1	1/6 (16.67%)	540.00 ± 254.09	7.83 ± 2.33	3.60
	D_2	3/6 (50%)	1700.00*** ± 55.14	2.17 ± 1.05	
	D_3	1/6 (16.67%)	960.00** ± 174.59	4.83 ± 1.19	
**7a**	D_1	0/6 (0%)	710.00 ± 74.97	8.67 ± 2.56	3.16
	D_2	1/6 (16.67%)	740.00 ± 253.46	5.00 ± 1.39	
	D_3	6/6 (100%)	1800.00*** ± 0.00	0.00** ± 0.00	
**8a**	D_1	1/6 (16.67%)	830.00* ± 230.52	2.00 ± 0.58	3.00
	D_2	2/6 (33.33%)	853.33* ± 308.96	2.17 ± 0.87	
	D_3	3/6 (50%)	1220.00*** ± 259.54	8.33 ± 3.96	
**9a**	D_1	1/6 (16.67%)	940.00** ± 277.42	3.67 ± 1.76	1.91
	D_2	0/6 (0%)	510.00 ± 207.12	6.67 ± 1.15	
	D_3	0/6 (0%)	1180.00*** ± 131.15	12.17 ± 3.09	
**D**	D = 2 mg/kg	6/6 (100%)	1800.00*** ± 0.00	0.00** ± 0.00	2.68
**Phe**	D = 15 mg/kg	6/6 (100%)	1800.00*** ± 0.00	0.00** ± 0.00	1.06

Comp. – compound (″**a**″ series); **NC** – negative control (solvent).; **D** – diazepam.;
Phe – phenobarbital;
$\mathbf{D_1}$ = 50 mg/kg; $\mathbf{D_2}$ = 100 mg/kg; $\mathbf{D_3}$ = 150 mg/kg;
[a] – **PTZ** – pentylenetetrazole; **(P)** - Number of mice protected (no seizures)/(**T**) - number of mice tested;
Tc – Time (seconds) until the first seizure ± **SEM** (Standard error of mean);
[b] – mean number of seizures in the first 30 minutes/animal lot ± **SEM** (Standard error of mean);
***p<0.001; **p<0.01; *p<0.05; Statistically significant compared to negative control group.

Table S2. Protection against PTZ-induced seizure model, time until the first seizure, mean number of seizures and LogP, for the compounds of the ″**b**″ series, in the first 30 minutes after PTZ administration.

Comp.	Doses (mg/kg)	PTZ P/T[a] (% protection)	Tc (s)	No. seizures[b]	LogP
**NC**	-	0/6 (0%)	111.67 ± 27.38	9.33 ± 1.65	-
**1b**	D_1	1/6 (16.67%)	1450.00*** ± 124.34	2.33*** ± 0.71	3.28
	D_2	2/6 (33.33%)	1190.00*** ± 198.04	1.17*** ± 0.47	
	D_3	1/6 (16.67%)	850.00** ± 205.77	2.33*** ± 0.71	
**2b**	D_1	0/6 (0%)	1100.00*** ± 150.73	5.67 ± 0.71	2.99
	D_2	0/6 (0%)	820.00** ± 97.57	5.17 ± 0.83	
	D_3	0/6 (0%)	580.00 ± 115.58	3.83* ± 1.35	
**3b**	D_1	0/6 (0%)	990.00*** ± 73.89	4.83 ± 0.79	3.73
	D_2	1/6 (16.67%)	950.00*** ± 174.87	3.33** ± 1.05	
	D_3	4/6 (66.66%)	1390.00*** ± 259.42	1.17*** ± 0.83	
**4b**	D_1	0/6 (0%)	900.00** ± 26.83	8.17 ± 2.32	3.13
	D_2	1/6 (16.67%)	590.00 ± 242.69	4.67 ± 1.72	
	D_3	4/6 (66.66%)	1500.00*** ± 189.73	0.67*** ± 0.49	
**5b**	D_1	0/6 (0%)	1030.00*** ± 47.53	6.50 ± 1.38	2.74
	D_2	1/6 (16.67%)	1020.00*** ± 168.29	3.83* ± 1.07	
	D_3	3/6 (50%)	1650.00*** ± 150.00	0.17*** ± 0.16	
**6b**	D_1	0/6 (0%)	780.00* ± 63.87	5.33 ± 0.84	3.65
	D_2	0/6 (0%)	510.00 ± 59.49	3.00** ± 0.96	
	D_3	1/6 (16.67%)	620.00 ± 241.83	2.50** ± 0.76	
**7b**	D_1	0/6 (0%)	460.00 ± 85.79	7.50 ± 2.07	3.74

Comp.	Doses (mg/kg)	PTZ P/T[a] (% protection)	T*c* (s)	No. seizures[b]	LogP
	D_2	0/6 (0%)	1110.00*** ± 150.80	3.00** ± 1.15	
	D_3	3/6 (50%)	1340.00*** ± 212.79	2.33*** ± 1.30	
**8b**	D_1	1/6 (16.67%)	1330.00*** ± 167.87	3.83* ± 1.81	3.29
	D_2	3/6 (50%)	1480.00*** ± 176.41	4.33* ± 2.43	
	D_3	6/6 (100%)	1800.00*** ± 0.00	0.00*** ± 0.00	
**9b**	D_1	0/6 (0%)	470.00 ± 76.55	6.00 ± 0.89	2.15
	D_2	0/6 (0%)	350.00 ± 36.05	5.33 ± 0.88	
	D_3	0/6 (0%)	300.00 ± 40.98	7.83 ± 1.22	
**D**	D = 2 mg/kg	6/6 (100%)	1800.00*** ± 0.00	0.00*** ± 0.00	2.68
**Phe**	D = 15 mg/kg	6/6 (100%)	1800.00*** ± 0.00	0.00*** ± 0.00	1.06

Comp. – compound (”**b**” series); **NC** – negative control (solvent).; **D** – diazepam.;
Phe – phenobarbital;
$\mathbf{D_1}$ = 50 mg/kg; $\mathbf{D_2}$ = 100 mg/kg; $\mathbf{D_3}$ = 150 mg/kg;
[a] – **PTZ** – pentylenetetrazole; **(P)** - Number of mice protected (no seizures)/(**T**) - number of mice tested;
Tc – Time (seconds) until the first seizure ± **SEM** (Standard error of mean);
[b] – mean number of seizures in the first 30 minutes/animal lot ± **SEM** (Standard error of mean);
***p<0.001; **p<0.01; *p<0.05; Statistically significant compared to negative control group.

2.2. Flumazenil antagonism assay

Table S3. Results obtained in flumazenil antagonism assay for the compounds **7a** and **8b**.

Comp.	Dose (mg/kg)	PTZ P/T[a] (% protection)	T*c* (s)	No. seizures[b]
**7a**	$\mathbf{D_3}$	0/6 (0%)	580.00 ± 59.32	5.17 ± 0.60
**8b**	$\mathbf{D_3}$	0/6 (0%)	500.00 ± 59.32	4.00 ± 0.77
**D**	**D = 2 mg/kg**	0/6 (0%)	591.00 ± 243.69	4.70 ± 1.75

Comp. – compound; **D** – diazepam; $\mathbf{D_3}$ = 150 mg/kg;
Tc – Time (seconds) until the first seizure (mean) ± **SEM** (Standard error of mean);
[a] - **PTZ** – pentylenetetrazole; **(P)** - Number of mice protected (no seizures)/(**T**) - number of mice tested;
[b] - mean number of seizures in the first 30 minutes/animal lot ± **SEM** (Standard error of mean);
